# Efficacy of GV1001 with gemcitabine/capecitabine in previously untreated patients with advanced pancreatic ductal adenocarcinoma having high serum eotaxin levels (KG4/2015): an open-label, randomised, Phase 3 trial

**DOI:** 10.1038/s41416-023-02474-w

**Published:** 2023-10-30

**Authors:** Jung Hyun Jo, Yong-Tae Kim, Ho Soon Choi, Ho Gak Kim, Hong Sik Lee, Young Woo Choi, Dong Uk Kim, Kwang Hyuck Lee, Eui Joo Kim, Joung-Ho Han, Seung Ok Lee, Chang-Hwan Park, Eun Kwang Choi, Jae Woo Kim, Jae Yong Cho, Woo Jin Lee, Hyungsik Roger Moon, Mi-Suk Park, Sangjae Kim, Si Young Song

**Affiliations:** 1 Division of Gastroenterology, Department of Internal Medicine, Severance Hospital, Yonsei University College of Medicine, Seoul, Korea; 2 Department of Internal Medicine and Liver Research Institute, Seoul National University College of Medicine, Seoul, Korea; 3 Department of Internal Medicine, Hanyang University College of Medicine, Seoul, Korea; 4 Department of Internal Medicine, Daegu Catholic University School of Medicine, Daegu, Korea; 5 Department of Gastroenterology, Korea University College of Medicine, Seoul, Korea; 6 Department of Internal Medicine, Konyang University College of Medicine, Daejeon, Korea; 7 Division of Gastroenterology and Hepatology, Biomedical Research Institute, Pusan National University Hospital, Busan, Korea; 8 Department of Medicine, Samsung Medical Center, Sungkyunkwan University School of Medicine, Seoul, Korea; 9 Division of Gastroenterology, Department of Internal Medicine, Gil Medical Center, Gachon University College of Medicine, Incheon, Korea; 10 Department of Internal Medicine, Chungbuk National University College of Medicine & Chungbuk National University Hospital, Cheongju, South Korea; 11 Department of Internal Medicine, The Research Institute for Medical Science, Jeonbuk National University Medical School, Jeonju, Korea; 12 Department of Internal Medicine, Chonnam National University Medical School, Gwangju, Korea; 13 Division of Gastroenterology, Department of Internal Medicine, Jeju National University College of Medicine, Jeju, Korea; 14 Department of Internal Medicine, Yonsei University Wonju College of Medicine, Wonju, Republic of Korea; 15 Department of Internal Medicine, Gangnam Severance Hospital, Yonsei University College of Medicine, Seoul, Korea; 16 Center for Liver and Pancreatobiliary Cancer, National Cancer Center, Goyang, Korea; 17 Department of Economics, University of Southern California, Los Angeles, CA USA; 18 Department of Economics, Yonsei University, Seoul, Korea; 19 Department of Radiology, Yonsei University College of Medicine, Severance Hospital, Seoul, Korea; 20 GemVax & KAEL Co., Ltd. 58, Techno 11-ro, Yuseong-gu, Daejeon, Republic of Korea

**Keywords:** Pancreatic cancer, Randomized controlled trials

## Abstract

**Background:**

The TeloVac study indicated GV1001 did not improve the survival of advanced pancreatic ductal adenocarcinoma (PDAC). However, the cytokine examinations suggested that high serum eotaxin levels may predict responses to GV1001. This Phase III trial assessed the efficacy of GV1001 with gemcitabine/capecitabine for eotaxin-high patients with untreated advanced PDAC.

**Methods:**

Patients recruited from 16 hospitals received gemcitabine (1000 mg/m^2^, D 1, 8, and 15)/capecitabine (830 mg/m^2^ BID for 21 days) per month either with (GV1001 group) or without (control group) GV1001 (0.56 mg; D 1, 3, and 5, once on week 2–4, 6, then monthly thereafter) at random in a 1:1 ratio. The primary endpoint was overall survival (OS) and secondary end points included time to progression (TTP), objective response rate, and safety.

**Results:**

Total 148 patients were randomly assigned to the GV1001 (*n* = 75) and control groups (*n* = 73). The GV1001 group showed improved median OS (11.3 vs. 7.5 months, *P* = 0.021) and TTP (7.3 vs. 4.5 months, *P* = 0.021) compared to the control group. Grade >3 adverse events were reported in 77.3% and 73.1% in the GV1001 and control groups (*P* = 0.562), respectively.

**Conclusions:**

GV1001 plus gemcitabine/capecitabine improved OS and TTP compared to gemcitabine/capecitabine alone in eotaxin-high patients with advanced PDAC.

**Clinical trial registration:**

NCT02854072.

## Introduction

Pancreatic ductal adenocarcinoma (PDAC) is a leading cause of cancer mortality with poor overall survival (OS) [1]. The 5-year survival rate for advanced PDAC is ~3% despite the recent improvement in combination therapies of FOLFIRINOX and gemcitabine/nab-paclitaxel [2, 3]. Recently, immunotherapy has emerged as the most promising therapeutic option for various cancers. Still, immunotherapy exhibits limited efficacy against PDAC.

Peptide vaccine GV1001 comprises 16 amino acids derived from the catalytic subunit of human telomerase reverse transcriptase (hTERT) [4], which is a widely expressed tumour-associated antigen and potentially applicable target for anticancer immunotherapeutic strategies [5]. In the TeloVac trial in Europe, the combination of GV1001 with gemcitabine/capecitabine (GemCap) did not show increased survival compared to gemcitabine/capecitabine in advanced PDAC patients [6]. However, the cytokine examinations of the TeloVac trial suggested that high serum eotaxin level (>81.02 pg/mL) may predict improved survival in patients who received GV1001 with gemcitabine/capecitabine [7].

Still, there have been no further investigations to prove the role of eotaxin in PDAC under GV1001 treatment. Hence, we planned a repeated Phase III randomised controlled trial, named KG4/2015, to re-analyse the efficacy of GV1001 therapy in Korean patients with PDAC who have high serum eotaxin levels.

## Methods

### Study overview

The trial was conducted according to the principles of the International Conference on Harmonisation on Good Clinical Practice and was reviewed by the Institutional Review Board at each participating institution. The clinical trial was contracted to LSK Global PS, Ltd (Seoul, Korea) as a Contract Research Organization (CRO). All participants provided written informed consent before randomisation. On the basis of previous studies [6, 7], on September 15, 2014, a new drug approval, under the name of Riavax^®^, was obtained conditionally from the Ministry of Food and Drug Safety (MFDS) of South Korea as a treatment for advanced pancreatic cancer.

### Study design and treatment

**Fig. 1:**
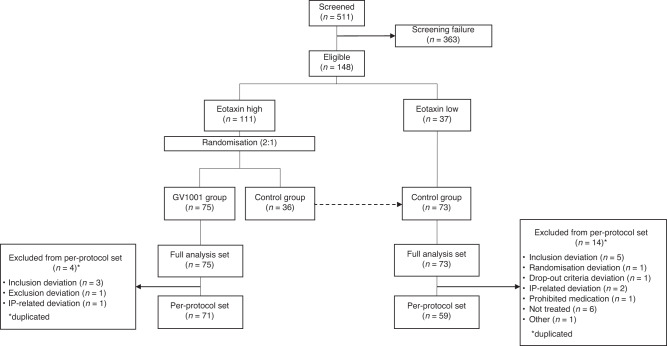
Flow diagram of patient disposition. A total of 511 pancreatic adenocarcinoma patients were screened, of 148 patients were enrolled. Patients with high serum eotaxin levels (>81.02 pg/mL) were randomly assigned in a 2:1 ratio to receive either Gemcitabine/Capecitabine with GV1001 (GV1001 group) or Gemcitabine/Capecitabine (control group). Finally, 148 patients were assigned to the GV1001 group (*n* = 75; all eotaxin-high) and control group (*n* = 73; 36 eotaxin-high and 37 eotaxin-low).

KG4/2015 was a randomised, prospective, controlled, open-labelled, multicenter, Phase III clinical trial performed at 16 Korean hospitals (ClinicalTrials.gov Identifier: NCT02854072) from November 2015 through April 2020. Figure 1 presents the scheme of study design and flow of patient disposition. The inclusion and exclusion criteria were similar to those in the TeloVac study. Briefly, eligible patients were treatment-naive, aged >18 years, with histologically or cytologically confirmed locally advanced or metastatic PDAC, previously untreated, had an Eastern Cooperative Oncology Group (ECOG) performance status of 0–2, and a life expectancy >3 months. We excluded patients if they had received radiotherapy within the last 8 weeks before the start of study, had peritoneal carcinomatosis leading to life expectancy of <3 months; intracerebral metastases or meningeal carcinomatosis; medication that might affect immunocompetence, such as long-term steroids or other immunosuppressants. Detailed inclusion and exclusion criteria are described in the study protocol (Supplementary Appendix 1).

Every eligible patient was checked for their serum eotaxin level at screening prior to randomisation: a total of 4 mL of blood was collected and shipped to the central laboratory (Seoul Clinical Laboratories, Seoul, Korea). The serum eotaxin level was quantified using the Bio-Plex Pro human cytokine singleplex (Eotaxin) assay kit with a Bio-Plex 200 System (Bio-Rad Laboratories, Inc, Hercules, CA, USA) in accordance with the manufacturer’s instructions.

Patients with high serum eotaxin levels (>81.02 pg/mL) were randomly assigned in a 2:1 ratio to receive either GemCap with GV1001 (GV1001 group) or GemCap (control group). Patients with low serum eotaxin levels were assigned only to the control group to ensure that the control group included eotaxin-high and eotaxin-low patients in a 1:1 ratio. Consequently, the GV1001 group consisted of patients with high serum eotaxin levels, and the control group consisted of eotaxin-high and eotaxin-low patients in a 1:1 ratio. GemCap treatment included gemcitabine (1000 mg/m^2^, 30 min intravenous infusion, on days 1, 8 and 15) and capecitabine (830 mg/m^2^ orally twice daily) for 21 days per a 28-day cycle. GV1001 group additionally received an intradermal injection of granulocyte–macrophage colony-stimulating factor (75 μg) and GV1001 (0.56 mg) on days 1, 3 and 5; followed by once a week during weeks 2, 3, 4 and 6; and monthly thereafter from the start of GemCap treatment.

Randomisation was stratified based on the stage of disease (locally advanced vs. metastatic). The allocation sequence was generated by the CRO and the patients were randomly assigned to the treatments by trained authorised staff. All patients and investigators were aware of the treatment allocation.

### Assessments

CT examinations of the chest, abdomen, and pelvis were performed in all treatment groups at screening, on week 8, and every 12 weeks thereafter until EOT. If CT scanning was not possible, magnetic resonance imaging (MRI) was performed. For those without documented disease progression at EOT, CT scans were obtained at follow-up visits scheduled every 12 weeks. The scanning parameters were at the discretion of each hospital. All images and case report forms were sent to the central review system and anonymized. In the central review system, two board-certified abdominal radiologists (with 21 and 12 years of experience, respectively) independently reviewed the CT or MRI images on a commercial workstation equipped with a 2000 × 2000 picture archiving and communication system (Centricity 3.0, General Electric Medical Systems, Milwaukee, WI, USA) monitor with the ability to adjust the optimal window setting for each patient. At baseline, the reviewers recorded the tumour location and size in the axial plane and evaluated the tumour’s relationship with the coeliac, superior mesenteric, and common hepatic arteries and superior mesenteric and portal veins as abutment (tumour-vessel contact less than or equal to 180°) or encasement (tumour-vessel contact more than 180°). The status of resectability was determined according to the National Comprehensive Cancer Network criteria. The presence, location, and size of metastases were also recorded and specific exclusion criteria including peritoneal carcinomatosis were reviewed. They compared the baseline and follow-up CT images and determined the anatomical response (complete response, partial response, stable disease, and progression) according to RECIST 1.1. Any discrepancy between the reviewers was resolved by a consensus review, and the consensus data were used for analysis.

### Study end points

For primary end points, OS was defined as the time from the date of the first randomisation to the date of death from any cause. For secondary end points, time to progression (TTP) was defined as the time from the date of the first randomisation to the date of tumour progression. Patients were followed up for survival until death, withdrawal, or study closure. The other secondary end points were objective tumour response rate (ORR), quality of life (QoL), changes in serum carbohydrate antigen 19-9 (CA 19-9) concentration over time, immunogenicity measured as T-cell proliferation, and safety.

Tumour outcome was evaluated at baseline, after 8 weeks, and every 12 weeks by CT or MRI until the end of the trial. Objective tumour response was defined as the ratio of patients confirmed with complete or partial responses. Tumour control rate was defined as the ratio of the patients confirmed with complete or partial responses or stable disease. Serum CA 19-9 was measured at baseline, at weeks 5, 8, 14 and 22 and every 4 weeks thereafter. Safety was monitored for treatment-related adverse events (AEs) and for serious AEs before each cycle of chemotherapy. Treatment-related AEs were graded according to the National Cancer Institute Common Terminology Criteria for Adverse Events (version 4.03).

### Statistical analysis

Full analysis set (FAS) population, per protocol set (PPS) population, and safety set analysis were defined according to the study protocol (Supplementary Appendix 1). All efficacy analyses were performed in the FAS population retaining all patients in their initially randomised groups irrespective of any protocol deviations. The safety set covered all patients who were administered the drug at least once.

The statistical details in sample size determination are described in the statistical analysis plan (SAP) in the study protocol (Supplementary Appendix 1). For the final analysis, the survival status of all patients was updated at the data-cutoff date (February 21, 2020). Data from patients who were alive were censored for the survival analysis. Data were analysed using the *χ*^*2*^ and Fisher exact for categorical data and the Student *t* test and Mann–Whitney *U* test for continuous variables. For comparison of survivals between the groups, the proportional hazard assumption was checked. If the assumption was satisfied, the Stratified Log-rank test was used. If not, the stratified generalised Wilcoxon test was used. Moreover, if the possibility of dependent censoring could not be excluded, the results of the dependent censoring model using a copula-graphic estimator method could be presented according to the SAP in the study protocol (Supplementary Appendix 1). The subgroup analyses comprised a Cox-proportional analysis and the Bonferroni test (to determine the family-wise error rate for multiple testing); these were performed using IBM SPSS Statistics for Windows, version 26.0 (IBM Corp, Armonk, NY).

### Correlative study

#### T-cell proliferation test and subpopulation analysis

We assessed T-cell proliferation in patients’ peripheral blood samples from the GV1001 group (*n* = 75). We thawed the peripheral blood mononuclear cells (PBMCs) at 1 × 10^6^ cells in RPMI 1640 (GIBCO, USA) with 10% human albumin serum (Sigma, USA) and penicillin-streptomycin (GIBCO, USA) with 2 µM CSFE (5, 6-carboxyfluorescein diacetate succinimidyl ester; BD Biosciences, USA). PBMCs were seeded in 96-well plates (BD Biosciences, USA) at 5 × 10^5^ cells per well. The cells were cultured with coated 1 µg/ml of Anti-CD3 (BD Biosciences, USA), 1 µg/ml of anti-CD28 (BD Biosciences, USA), and further incubated with 20 µg/ml of GV1001 for stimulation in 4 days at 37 °C. After 4 days, the cells were harvested, stained with fluorochrome-labelled anti-CD3 (BD Biosciences, USA), and analysed using flow cytometry (FACSVerse, BD Biosciences). We defined a positive proliferative response to GV1001 as a stimulation index >2 with a significant difference in CFSE fluorescence compared between days 0 and 4. For subpopulation analysis, we stained 1 × 10^6^ PBMC with anti-CD3, anti-CD4, and anti-CD8 (BD Biosciences, USA) and analysed them using flow cytometry (FACSVerse, BD Biosciences).

#### Protein array

The protein was extracted from plasma using a protein extraction buffer (Fullmoon biosystems, Sunnyvale, CA) and protein expression was analysed using antibody microarray analysis (Fullmoon biosystems) according to the manufacturer’s protocol. In brief, 50 μg of the protein sample was labelled and incubated with a coupling mixture on the antibody microarray slide (Fullmoon biosystems) and detected with Cy3-streptavidin (GE Healthcare, Chalfont St. Giles, UK). The slide was rinsed and scanned using GenePix 4100 A (Axon Instrument, USA) at a 10 μm resolution, optimal laser power, and PMT. After obtaining the scanned images, they were grided and quantified with GenePix 7.0 Software (Axon Instrument, USA). The data about protein information was annotated using UniProt DB.

## Results

### Patients

This clinical trial was conducted at 16 academic hospitals in Korea, from November 2015 through April 2020. A total of 511 patients underwent a screening test after providing written informed consent (Fig. 1). During the screening, every patient was checked for their serum eotaxin level, where 174 (34.1%) of them had elevated serum eotaxin level over the cutoff of 81.02 pg/mL. Upon not meeting the inclusion/exclusion criteria, 363 patients were eliminated from screening. Details of the screened patients are presented in Supplementary Table S1. Finally, 148 patients were randomly assigned to the GV1001 group (*n* = 75; all eotaxin-high) and control group (*n* = 73; 36 eotaxin-high and 37 eotaxin-low). The demographic information and other pretreatment characteristics of 148 patients are shown in Table 1.

**Table 1:** Demographic and baseline characteristics of the patients.

	Total (*N* = 148)	GV1001 (*N* = 75)	Control (*N* = 73)	*P* value
Age (years), mean (SD)				
Mean (SD)	63.1 (9.1)	64.2(8.7)	62.0 (9.4)	0.140
Sex, *n* (%)				
Male	80 (54.1)	34 (45.3)	46 (63.0)	0.031
Female	68 (45.9)	41 (54.7)	27 (37.0)	
Smoking, *n* (%)				
Current	19 (12.8)	12 (16.0)	7 (9.6)	0.236
Past	50 (33.8)	21 (28.0)	29 (39.7)	
No	79 (53.4)	42 (56.0)	37 (50.7)	
Alcohol, *n* (%)				
Current	27 (18.2)	12 (16.0)	15 (20.6)	0.193
Past	43 (29.1)	18 (24.0)	25 (34.3)	
No	78 (52.7)	45 (60.0)	33 (45.2)	
Primary tumour site within pancreas				
Head	71 (48.0)	39 (52.0)	32 (43.8)	0.778
Body	43 (29.1)	19 (25.3)	24 (32.9)	
Tail	23 (15.5)	12 (16.0)	11 (15.1)	
Overlapping	11 (7.5)	5 (6.7)	6 (8.2)	
Pancreatic cancer status, *n* (%)				
Locally advanced	36 (24.3)	20 (26.7)	16 (21.9)	0.501
Metastatic	112 (75.7)	55 (73.3)	57 (78.1)	
Metastasis*, *n* (%)				
Lymph Node	45 (30.4)	20 (26.7)	25 (34.2)	0.769
Bone	3 (2.0)	1 (1.3)	2 (2.7)	
Liver	88 (59.5)	42 (56.0)	46 (63.0)	
Lung	18 (12.2)	10 (13.3)	8 (11.0)	
Brain	0 (0)	0 (0)	0 (0)	
Others	15 (10.1)	4 (5.3)	11 (15.1)	
CA 19-9 concentration (IU/L)				
Mean (SD)	5229.0 (17,449.0)	4032.5 (12,500.7)	6458.2 (21,400.6)	0.400
ECOG performance status, *n* (%)				
0	89 (60.1)	45 (60.0)	44 (60.3)	0.769
1	55 (37.2)	27 (36.0)	28 (38.3)	
2	4 (2.7)	3 (4.0)	1 (1.4)	

*SD* standard deviation.

*Multiple organ metastases were counted separately.

### Efficacy

#### Survival

The median follow-up time of all patients was 7.9 months (95% confidence interval [CI], 6.6–9.2). The OS analysis was based on 80 deaths (54.1%), including 46 (61.3%) and 34 (46.6%) patients in the GV1001 and control groups, respectively. The proportion of censored patients for survival analysis showed a large difference between the GV1001 and control groups (38.7% vs. 53.4%, details in Supplementary Table S2). There were more dropouts due to withdrawal of consent in the control group than in the GV1001 group (43.8% vs. 24.0%, *P* = 0.011). Moreover, we checked the sample correlation coefficient between the survival and censoring time of those whose survival time and censoring time during disease progression were observed. The sample correlation coefficient between the logarithms of OS and TTP and between the logarithms of TTP and censoring time were as high as 0.8 and 0.94 in the GV1001 group and 0.9 and 0.9 in the control group. Therefore, we concluded that we could not exclude the possibility of dependent censoring. Following the SAP, we estimated the survival function under dependent censoring using the copula-graphic estimation method. Detailed statistical methods of survival analysis are presented in our previous report [8].

**Fig. 2:**
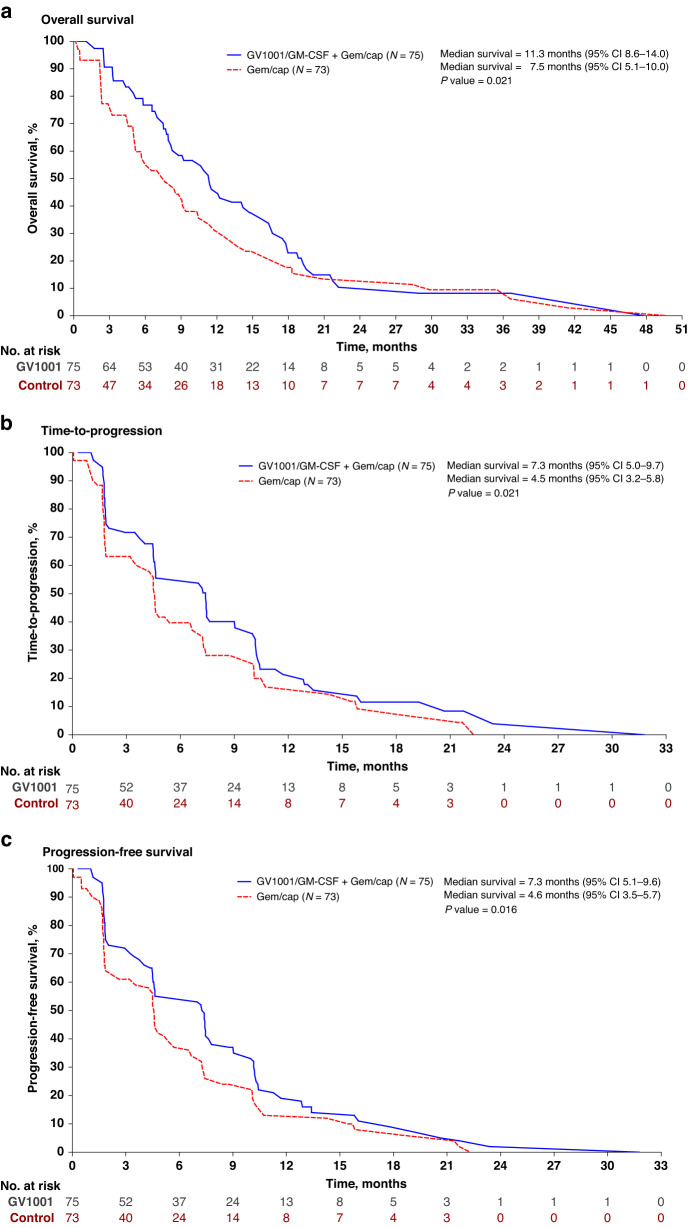
Survival estimates curve of overall survival, time-to-progression, and progression-free survival in the full assessment set population. **a** Overall survival, **b** time-to-progression and **c** progression-free survival.

In the FAS population, median OS was significantly improved in the GV1001 group at 11.3 months [95% CI, 8.6–14.0] than in the control group at 7.5 months [95% CI, 5.1–10.0] (*P* = 0.021) (Table 2 and Fig. 2a). In the PPS population, median OS was significantly improved in the GV1001 group at 11.3 months [95% CI, 8.3–14.3] than in the control group at 7.5 months [95% CI, 4.9–10.2] (*P* = 0.031). The TTP analysis was based on 91 events (61.5%), including 51 (68.0%) and 40 (54.8%) events in the GV1001 and control groups, respectively. In the FAS population, median TTP was significantly improved in the GV1001 group (7.3 months [95% CI, 5.0–9.7]) compared to the control group (4.5 months [95% CI, 3.2–5.8], *P* = 0.021) (Table 2 and Fig. 2b). In the PPS population, median TTP was significantly improved in the GV1001 group at 7.2 months [95% CI, 4.9–9.6] compared to the control group at 4.6 months [95% CI, 2.9–6.2] (*P* = 0.034). The progression-free survival (PFS) was additionally analysed in FAS population. The PFS analysis was based on 115 events (77.7%), including 63 in the GV1001 group (84.0%) and 52 in the control group (71.2%). The median PFS was significantly improved in the GV1001 group (7.3 months [95% CI, 5.1–9.6]) compared to the control group (4.6 months [95% CI, 3.5–5.7], *P* = 0.016) (Table 2 and Fig. 2c).

**Table 2:** Survival and treatment responses in the full analysis set population.

	GV1001 (*N* = 75)	Control (*N* = 73)
Overall survival, mo (95% CI)*	11.3	7.5
95% confidence interval	[8.6–14.0]	[5.1–10.0]
* P* value	0.021	
Time to progression, mo (95% CI)*	7.3	4.5
95% confidence interval	[5.0–9.7]	[3.2–5.8]
* P* value	0.021	
Progression-free survival, mo (95% CI)*	7.3	4.6
95% confidence interval	[5.1–9.6]	[3.5–5.7]
*P* value	0.016	
Objective response rate, *n* (%)	20 (26.7)	20 (27.4)
95% confidence interval	[16.7–36.7]	[17.2–37.6]
*P* value	0.920	
Disease control rate, *n* (%)	54 (72.0)	46 (63.0)
95% confidence interval	[61.8–82.2]	[51.9–74.1]
*P* value	0.243	
Best overall response, *n* (%)		
Complete response (CR)	0	0
Partial response (PR)	20 (26.7)	20 (27.4)
Stable disease (SD)	34 (45.3)	26 (35.6)
Progressive disease (PD)	16 (21.3)	15 (20.6)
Not evaluable (NE)	1 (1.3)	0
Others	4 (5.3)	12 (16.4)

*CR* complete response, *PR* partial response, *SD* stable disease.

^*^Survival analysis used the copula-graphic estimator method under dependent censoring.

Objective response rate (%) = proportion of participants with a response of CR or PR. Disease control rate (%) = proportion of participants with a response of CR, PR or SD.

Note: The denominator of the percentage is the number of participants in each group.

Furthermore, to evaluate the correlation between baseline eotaxin levels and treatment outcomes, a Cox-proportional analysis of the contribution of serum eotaxin level to the OS was performed in the GV1001 and control groups of the FAS population (Supplementary Table S3); the included variables were the serum eotaxin level, age, sex, and disease status. Multivariate analysis revealed that eotaxin level was not associated with OS in the GV1001 (hazard ratio [HR], 1.012 [95% CI, 0.999–1.024]; *P* = 0.065) and control groups (HR, 1.007 [95% CI, 0.997–1.017]; *P* = 0.160).

#### Response

According to the RECIST criteria, 20 participants demonstrated an ORR (26.7% [95% CI, 16.7–36.7%]) out of 75 patients in the GV1001 group and 20 patients (27.4% [95% CI, 17.2–37.6%]) in the control group, and all were partial response (PR) (Table 2). The difference between the two groups for the ORR was not statistically significant (*χ*^2^ test, *P* = 0.920). Fifty-four participants showed a disease control rate (72.0% [95% CI, 61.8–82.2%]) in the GV1001 group and 46 patients (63.0% [95% CI, 51.9–74.1%]) in the control group, without significant difference between the groups (*χ*^2^ test, *P* = 0.243).

#### Subgroup analyses

Subgroup analyses according to demographics were performed between the two groups (Supplementary Table 4). The GV1001 group showed increased median OS compared to the control group within the young age (≤65, *P* = 0.036), metastatic disease (*P* = 0.006), higher ECOG performance status (PS) (1 or 2, *P* = 0.010), and primary tumour location in body or tail (*P* = 0.045) subgroups. The GV1001 group showed increased median TTP compared to the control group within the male (*P* = 0.045) and higher ECOG PS (1 or 2, *P* = 0.019) subgroups.

### Safety

Of 142 safety set participants, 2051 AEs occurred in 140 patients (98.59%). A total of 1107 AEs were found in 75 patients (100%) in the GV1001 group, and 944 AEs were found in 65 patients (97.01%) in the control group, and there was no statistically significant difference in the incidence of AEs between the two groups (*P* = 0.221). AEs ≥grade 3 were reported in 58 cases (77.3%) and 49 cases (73.1%) in the GV1001 group and control group, respectively, without significant difference (*P* = 0.562). Treatment-related grade 3 or 4 AEs that occurred in two or more patients are summarised in Table 3 and more details are presented in Supplementary Table S5. Most common AEs ≥grade 3 were haematologic in nature. Haematologic AEs were reported as 57.3% vs. 50.8% of neutropenia, 16% vs. 13.4% of anaemia, 9.3% vs. 13.4% of thrombocytopenia, and 12.0% vs. 9.0% of leukopenia in the GV1001 group and control group, respectively. There were no significant differences in the occurrence of each AE between groups. AEs reported in clinical laboratory tests are mostly due to gemcitabine or capecitabine, and these AEs were considered to be controllable after reduction and intermittent cessation of gemcitabine or capecitabine.

**Table 3:** Summary of treatment-related adverse events occurring in the safety set population.

Event	GV1001 (*N* = 75)	Control (*N* = 67)
Adverse event led to death, no (%)	2 (2.7)	4 (6.0)
*P* value	0.421	
Adverse event led to discontinuation, no (%)	12 (16.0)	13 (19.4)
* P* value	0.595	
Adverse event with Grade ≥ 3, no (%)	58 (77.3)	49 (73.1)
*P* value	0.562	
Haematologic adverse event with Grade ≥ 3, no (%)		
Neutropenia	43 (57.3)	34 (50.8)
Febrile neutropenia	1(1.3)	1 (1.5)
Thrombocytopenia	7 (9.3)	9 (13.4)
Anaemia	12 (16.0)	9 (13.4)
Leukopenia	9 (12.0)	6 (9.0)
Non-haematologic adverse event with Grade ≥ 3, no (%)*		
Blood bilirubin increased	1 (1.33)	6 (8.96)
Gamma-glutamyltransferase increased	1 (1.33)	2 (2.99)
Nausea	2 (2.67)	0 (0.00)
Stomatitis	1 (1.33)	2 (2.99)
Duodenal obstruction	3 (4.00)	0 (0.00)
Asthenia	5 (6.67)	2 (2.99)
Palmar-plantar erythrodysesthesia syndrome	2 (2.67)	2 (2.99)
Decreased appetite	2 (2.67)	0 (0.00)
Hyperglycaemia	2 (2.67)	0 (0.00)
Pulmonary embolism	2 (2.67)	1 (1.49)
Hypotension	4 (5.33)	0 (0.00)
Acute kidney injury	3 (4.00)	0 (0.00)
Chronic kidney disease	0 (0.00)	2 (2.99)

^*^Occurred in ≥ 2 patients in either group.

### Quality of life scores

The results of the EORTC QLQ-C30 and EQ-5D in the FAS, including Global QoL, functional scales (physical, role, cognitive, emotional, and social), and symptom scales (fatigue, pain, nausea/vomiting, and appetite) from the EORTC QLQ-C30 questionnaire and the five items in the EQ-5D-5L (mobility, self-care, usual activities, pain/discomfort, and anxiety/depression) as well as today’s health status were similar between the treatment groups at baseline. There were items and time points that showed statistically significant differences between the groups, but the differences between the groups were probably because of the omission of results from the time point after 20 weeks, as the number of subjects that dropped out of both groups increased.

### Correlative study

#### Immunogenicity

The T-cell proliferation test was performed only in the GV1001 group, and the number and percentage of patients in FAS population with positive T-cell proliferation test was 19 patients (25.33%) at week 1, 14 patients (18.67%) at week 10, 14 (18.67%) patients at week 14, and 12 patients (16·00%) at week 18. A total of 35 patients with positive result in T-cell proliferation tests reported median OS as 12.3 months (95% CI, 7.1–17.5), which was not significantly increased than 40 patients with negative T-cell proliferation tests (10.6 months [95% CI, 5.7–15.5], *P* = 0.317).

In addition, CD8^+^/CD4^+^ T-cell subpopulation was analysed in available blood samples from 35 patients of the GV1001 group. Of the 35 patients, CD8^+^ T-cell subpopulation was upregulated in 21 patients (60%) with significantly increased median OS (15·3 months [95% CI, 10.5–20.0]) compared to 14 patients (40%) without upregulation of CD8^+^ T-cell subpopulation (8·4 months [95% CI, 4.6–12.1], *P* = 0.012). Next, to evaluate the relationship between baseline CD8^+^ T-cell count and survival, patients were divided into the CD8^+^-high (*n* = 17) and CD8^+^-low (*n* = 18) groups based on the baseline CD8^+^ T-cell subpopulation (in percentage); the median OS did not differ significantly between the two groups (11.7 months [95% CI, 7.7–15.6 months] vs. 11.8 months [95% CI, 5.9–17.7 months]; *P* = 0.512). These results suggest a response to GV1001 can be predicted by T-cell proliferation status of patients.

#### Protein array

To determine potential markers to predict chemoresponse to GV1001, patients’ samples at the time of pretreatment were analysed using antibody microarray analysis. According to survival, good responders and poor responders were selected from the GV1001 and control groups to compare the difference of marker expression by patients’ treatment response. A total of 73 patients’ samples were analysed; 49 samples from the GV1001 group (22 good responders and 27 poor responders) and 24 samples from the control group (13 good responders and 11 poor responders). We selected the protein markers with increased expression in good responders compared to poor responders from the GV1001 group. Moreover, to exclude response markers for gemcitabine/capecitabine, protein markers that showed a difference in expression between good and poor responders from the control group were excluded. As a result, we selected 10 protein marker candidates to predict the response for GV1001 (Supplementary Table S6). These included nerve growth factor (NGF) beta, vascular endothelial growth factor B (VEGFB), insulin-like growth factor II (IGF-II), matrix metalloproteinases ([MMPs], namely MMP-2 and MMP-10), Catenin-alpha1, Catenin-gamma, soluble tumour necrosis factor (sTNF)-receptor II, TNF-beta, and Heregulin. NGF beta, VEGFB, and IGF-II are growth factors related to pancreatic cancer progression [9–13]. MMPs are well-known factors associated with the tumour microenvironment (TME); MMP-2 and MMP-10 were identified as diagnostic indicators for PDAC and its progression in several studies [14, 15]. Catenin-alpha1 and catenin-gamma are members of the catenin family associated with cell adhesion in cancer progression. sTNF-receptor II and TNF-beta are related to the TNF pathway underlying TME inflammation in PDAC [16–18]. Finally, heregulin is known to drive PDAC development through ErbB receptor-mediated signalling [19, 20].

When comparing survival according to the expression of protein marker candidates, MMP-2 and Catenin-alpha1 presented significantly increased PFS in the high-expressed group compared to low low-expressed group (Table 4). Moreover, MMP-2 showed significantly increased OS in the high-expressed group compared to the low-expressed group. Additionally, multivariate Cox-proportional analyses were performed to evaluate the contribution of a high expression of the protein marker candidates to survival (Supplementary Table S7). A high expression of Catenin-alpha1 (HR, 0.37 [95% CI, 0.20–0.068]; *P* = 0.002) was associated with a prolonged PFS in the GV1001 group. A high expression of MMP-2 was associated with a prolonged OS in the GV1001 group. (HR, 0.37 [95% CI, 0.18–0.076]; *P* = 0.007); however, this association was not significant (i.e., *P* was not >0.005) following Bonferroni correction. These findings suggest that MMP-2 and Catenin-alpha1 can serve as biomarkers to predict GV1001 response in patients with PDAC.

**Table 4:** Comparison of survival according to the expression of protein marker candidates.

Gene	Antibody name	Overall survival, months (95% CI)			Progression-free survival, months (95% CI)		
		Low expressed	High expressed	*P* value^†^	Low expressed	High expressed	*P* value^†^
NGF	NGF beta	7.9 (4.2–11.7)	16.3 (12.9–19.7)	0.229	2.9 (1–4.9)	10.2 (8.3–12.1)	0.146
MMP-2	MMP-2	9.2 (6.1–12.4)	16.5 (13.6–19.4)	0.005	3.3 (1.2–5.3)	10.4 (10.1–10.7)	0.001
NRG1	Heregulin	11.5 (1–22.1)	14.7 (11.5–18)	0.108	3.3 (0.7–5.9)	10 (8.3–11.7)	0.088
VEGFB	VEGFB	11.5 (2.9–20.1)	14.7 (10.2–19.2)	0.214	3.5 (0.9–6.1)	10.3 (5–15.6)	0.006
MMP-10	MMP-10	12.1 (5.2–18.9)	14.7 (10.6–18.9)	0.335	1.9 (0–4.2)	9 (5.2–12.9)	0.026
CTNNA1	Catenin-alpha1	9.2 (5.1–13.3)	16.5 (12.5–20.5)	0.035	3.5 (2.2–4.8)	10.3 (7.1–13.5)	0.001
TNFRSF1B	sTNF-receptor II	9.2 (2.8–15.7)	15.6 (12.3–18.9)	0.848	1.8 (1.6–2.1)	10.2 (8.3–12.1)	0.221
IGF2	IGF-II	8.3 (5.9–10.6)	14.7 (11.2–18.2)	0.311	2.9 (0.6–5.2)	10.2 (9.8–10.6)	0.114
JUP	Catenin-gamma	10.6 (2.4–18.9)	15.6 (11.7–19.5)	0.894	2.9 (1–4.9)	9 (4.5–13.6)	0.351
LTA	TNF-beta	13.3 (0.5–26)	14.2 (9.6–18.8)	0.580	3.3 (0.5–6)	8.8 (2.1–15.5)	0.813

^†^Using Log-rank test with Kaplan–Meier survival analysis. A significant *P* value of 0.005 was determined by applying the Bonferroni Method to determine the family-wise error rate for multiple testing.

## Discussion

In the present clinical trial, the efficacy evaluation was conducted in 148 patients of the FAS population, and the median OS was 11.3 months in the GV1001 group and 7.5 months in the control group, with a significant difference (*P* = 0.021). The median TTP was also significantly longer in the GV1001 group at 7.3 months compared to the control group at 4.5 months (*P* = 0.021). These are similar results compared to those of the current standard chemotherapies including gemcitabine/nab-paclitaxel or FOLFIRINOX [2, 3]. The previous TeloVac study showed that the median OS was 7.9 months and 8.4 months in the chemotherapy group and the concurrent chemoimmunotherapy group (same as the control group and the GV1001 group of the present study), respectively [6]. Overall, the GV1001 combination treatment was confirmed as having better OS and TTP than the gemcitabine/capecitabine treatment without increasing toxicity in patients with advanced PDAC having high serum eotaxin levels.

Our study was conducted to confirm the clinical implication of serum eotaxin levels, suggested as predictive markers for improved survival in patients with PDAC who received GV1001 treatment according to the previous subgroup analysis of the TeloVac study [7]. Our study is the first to report serum eotaxin levels in a large population of Asian patients with pancreatic cancer. Herein, 34.1% of the 511 patients had elevated serum eotaxin levels over the cutoff. This means that GV1001 could be a treatment option in about one-third of patients with pancreatic cancer if GV1001 proves to be effective in patients with PDAC having high serum eotaxin levels. The final outcome of our study revealed that GV1001 treatment was effective in improving the survival of patients with high serum eotaxin levels. Further research is needed on the mechanism of eotaxin and its association with GV1001.

Our study substantiated the relationship between serum eotaxin level and the efficacy of GV1001 treatment in a prospective randomised study. However, not all patients with high serum eotaxin levels presented a favourable response to GV1001. To determine potential biomarkers to predict the response to GV1001 treatment, we conducted a correlative study with serum protein array before treatment. The correlative study suggested MMP-2 and Catenin-alpha1 as potential serological protein markers to predict response to the GV1001 based on patients’ survival. Pretreatment serum levels of MMP-2 and Catenin-alpha1 were significantly related to survivals of the GV1001 group. The activity of MMPs is critical for cancer cells to invade through extracellular matrices. PDAC is characterised by a strong tumour microenvironment and contains many proteases consisting of MMPs [21]. Experimental studies frequently use MMPs, including MMP-2, as indicators for the diagnosis and progression of PDAC [14]. However, there have been no reports about the correlation of MMP-2 and GV1001 in PDAC, while combined detections of telomerase activity and MMP-2 protein have been suggested as a risk factor of recurrence in gastric cancer [22]. Catenin-alpha1 is a member of the catenin family of proteins that play an important role in the cell adhesion process and are associated with the endothelial-mesenchymal transition (EMT) of cancer progression [16, 23, 24]. Several studies have reported that hTERT and telomerase activities regulate the EMT process in various cancers; however, there has been no definite evidence of EMT process related to GV1001 [25, 26]. Although the mode of action of GV1001 was considered as that similar to cancer immunotherapy in this study, other interactions between GV1001 and cancer progression have been reported [27, 28]. These results could be a clue to reveal another mode of action of GV1001 related to these markers. Considering all patients of the GV1001 group had high serum eotaxin levels, MMP-2 and Catenin-alpha1 may be markers that respond to GV1001 in association with eotaxin. Since it is not possible to draw a robust conclusion due to the limitation of the patient composition of this study, further studies on these markers and the therapeutic response of GV1001 are required.

Alteration of T-cell proliferation could be another predictive marker for GV1001. GV1001 can penetrate cell membranes and is endogenously processed by proteasome-mediated degradation followed by Major Histocompatibility Complex mediated surface expression of telomere peptide fragments [28–30]. Finally, GV1001 induced strong CD4^+^ and CD8^+^ response and recognition by antigen presenting cells [31, 32]. In previous Phase I/II studies, GV1001-specific T-cell responses have been seen in 50–80% of patients with PDAC and non-small cell lung cancer (NSCLC) [32, 33]. Moreover, an 8-year update of Phase I/II trial of NSCLC reported that GV1001 vaccination induced long-term T-cell memory against telomerase antigens, while not compromising bone marrow function [34]. In the TeloVac trial, T-cell proliferation was positive in 25 (37%) of 68 patients in concurrent chemoimmunotherapy group [6]. Phase I/II trial of PDAC presented immune responders defined by in vitro tests including T-cell proliferation that survived longer than the non-responders [32]. In our study, the T-cell proliferation test in the GV1001 group presented 46·7% (35/75 patients) of positive results, similar to previous reports. Patients with positive results in the T-cell proliferation test did not show prolonged OS compared to those with negative results. However, when the CD8^+^ T-cell subpopulation was analysed from patients with positive results, those in the upregulation of CD8^+^ T-cell subpopulation showed significantly increased median OS compared to others. Although our results were analysed in a small number of patients, they suggest that the CD8^+^ T-cell subpopulation can be used to predict the treatment response of GV1001. Furthermore, these data indicated that the benefit of GV1001 administration may be immune-mediated, suggesting that the treatment response may be transient if the immune memory response is insufficient. Further studies on lymphocytes and their phenotypic changes are needed to identify clues and potential targets for promoting immunological memory in GV1001 treatment.

In analysing survival data, the most widely used estimation methods of the survival function are the Kaplan–Meier estimation and Cox’s proportional hazard estimation. The log-rank test and Gehan’s generalised Wilcoxon test are often used to test for equality of the survival functions between the treatment and control groups. The outcomes of these estimations and tests are valid only if censoring is random, which is also called the uninformative censoring or independent censoring condition. Random censoring means that the time to censoring is not associated with the time to death. However, informative or dependent censoring can occur when participants are lost to follow-up due to reasons related to the study, such as drug toxicity, patient preference, or inadequate response [35]. As shown in Supplementary Table S2, the censoring that occurred in this study was not random and was context-dependent. If the random censoring assumption is violated, the statistical methods planned in the survival data analysis, the log-rank test, Gehan’s generalised Wilcoxon test, the Kaplan–Meier estimate, and the Cox-proportional hazard estimate do not provide a valid inference and estimation result. Therefore, in the survival analysis of this study, in consideration of the dependent censoring, we modelled the dependence using a parametric cupola [8]. Herein, first, we check the independence of censoring and survival times, and we found statistical evidence of the violation of assumption of the independence of censoring. It is probably because this study was an open-label study and pancreatic cancer has a relatively short survival period. Secondly, to estimate the survival function of the OS under dependent censoring following the SAP, we use the copula-graphic estimation method [36–38]. It is well-known that the copula-graphic estimator in dependent censoring corresponds to the Kaplan–Meier estimator in independent censoring. Our estimation results show that the median OS is 11.3 months in the GV1001 group, while it is 7.5 months in the control group. The difference of the median OS is between 3.8 months, and this difference is statistically significant at the 2.5% level with a p value 0.021. The relevant materials are detailed in the our previous report [8].

This study has some limitations. First, the treatment regimen in the control group was gemcitabine/capecitabine rather than gemcitabine/nab-paclitaxel, which is the current standard gemcitabine-based chemotherapy for PDAC, because of regulatory difficulties in nab-paclitaxel use at the time of initiation of the current clinical trial in Korea. Moreover, there is no evidence that serum eotaxin levels function as a predictive marker to GV1001 efficacy even when used in combination with therapies other than gemcitabine/capecitabine. Further clinical trials to verify the effect of GV1001 using standard chemotherapy as control should be considered. To overcome this limitation, we plan to evaluate the effect of GV1001 in combination with current standard chemotherapy, FOLFORINOX, or a gemcitabine/nab-paclitaxel regimen in a large-scale randomised controlled Phase III clinical trial. The second issue relates to the statistical method used in the survival function estimation under dependent censoring. As mentioned earlier in the main part of the paper, we could not exclude the possibility of dependent censoring, and following the SAP, we used the copula-graphic estimator method in estimating the survival function instead of the Kaplan–Meier method, which is the widely used standard estimation method that is valid only under random censoring situation. The external statisticians verified the specific estimation method used in the paper [8]. Non-random censoring issues in clinical trials have been discussed extensively in statistical and clinical literature [35, 39, 40]. However, the literature does not agree on a standard survival analysis method that provides a valid statistical result under dependent censoring in clinical trials. The copula-graphic estimator method used in the paper is one of the methods proposed in literatures [36–38]. Considering its clinical relevance and its consequential importance when it is ignored, survival analysis under dependent censoring should be considered more widely, for which it is necessary to further develop statistical methods that researchers can reach a consensus on.

In conclusion, GV1001 with GemCap treatment significantly extends the OS and TTP compared to GemCap in patients with advanced PDAC having high serum eotaxin levels, and specific safety-related issues have not been found. Therefore, GV1001 should be considered as one of the options in patients with advanced PDAC having high serum eotaxin levels.

### Supplementary information

Supplementary tables - revised

Supplementary appendix1_KG4-2014_Study protocol

## Data Availability

The raw protein microarray data generated in our study is available in the GEO database under accession number GSE212575. Data that support the findings of this study are available from the corresponding author upon request.

## Code availability

An R package of the copula estimator for survival analysis has been provided at https://github.com/zhan-gao/CopulaGraphic.
