# Correction: Efficacy of GV1001 with gemcitabine/capecitabine in previously untreated patients with advanced pancreatic ductal adenocarcinoma having high serum eotaxin levels (KG4/2015): an open-label, randomised, Phase 3 trial

**DOI:** 10.1038/s41416-023-02520-7

**Published:** 2023-12-04

**Authors:** Jung Hyun Jo, Yong-Tae Kim, Ho Soon Choi, Ho Gak Kim, Hong Sik Lee, Young Woo Choi, Dong Uk Kim, Kwang Hyuck Lee, Eui Joo Kim, Joung-Ho Han, Seung Ok Lee, Chang-Hwan Park, Eun Kwang Choi, Jae Woo Kim, Jae Yong Cho, Woo Jin Lee, Hyungsik Roger Moon, Mi-Suk Park, Sangjae Kim, Si Young Song

**Affiliations:** 1 Division of Gastroenterology, Department of Internal Medicine, Severance Hospital, Yonsei University College of Medicine, Seoul, Republic of Korea; 2 Department of Internal Medicine and Liver Research Institute, Seoul National University College of Medicine, Seoul, Republic of Korea; 3 Department of Internal Medicine, Hanyang University College of Medicine, Seoul, Republic of Korea; 4 Department of Internal Medicine, Daegu Catholic University School of Medicine, Daegu, Republic of Korea; 5 Department of Gastroenterology, Korea University College of Medicine, Seoul, Republic of Korea; 6 Department of Internal Medicine, Konyang University College of Medicine, Daejeon, Republic of Korea; 7 Division of Gastroenterology and Hepatology, Biomedical Research Institute, Pusan National University Hospital, Busan, Republic of Korea; 8 Department of Medicine, Samsung Medical Center, Sungkyunkwan University School of Medicine, Seoul, Republic of Korea; 9 Division of Gastroenterology, Department of Internal Medicine, Gil Medical Center, Gachon University College of Medicine, Incheon, Republic of Korea; 10 Department of Internal Medicine, Chungbuk National University College of Medicine & Chungbuk National University Hospital, Cheongju, South Korea; 11 Department of Internal Medicine, The Research Institute for Medical Science, Jeonbuk National University Medical School, Jeonju, Republic of Korea; 12 Department of Internal Medicine, Chonnam National University Medical School, Gwangju, Republic of Korea; 13 Division of Gastroenterology, Department of Internal Medicine, Jeju National University College of Medicine, Jeju, Republic of Korea; 14 Department of Internal Medicine, Yonsei University Wonju College of Medicine, Wonju, Republic of Korea; 15 Department of Internal Medicine, Gangnam Severance Hospital, Yonsei University College of Medicine, Seoul, Republic of Korea; 16 Center for Liver and Pancreatobiliary Cancer, National Cancer Center, Goyang, Republic of Korea; 17 Department of Economics, University of Southern California, Los Angeles, CA USA; 18 Department of Economics, Yonsei University, Seoul, Republic of Korea; 19 Department of Radiology, Yonsei University College of Medicine, Severance Hospital, Seoul, Republic of Korea; 20 GemVax & KAEL Co., Ltd. 58, Techno 11-ro, Yuseong-gu, Daejeon, Republic of Korea

**Keywords:** Pancreatic cancer, Randomized controlled trials

Correction to: *British Journal of Cancer* 10.1038/s41416-023-02474-w, published online 30 October 2023

In this article the unit of eotaxin blood concentration should be pg/mL, but there were two typos in ng/mL. This has been corrected.

In section “Methods”, Study design and treatment, 3rd paragraph: Patients with high serum eotaxin levels (>81.02 ng/mL) were randomly assigned in a 2:1 ratio to receive either GemCap with GV1001 (GV1001 group) or GemCap (control group).

It should read:

Patients with high serum eotaxin levels (>81.02 pg/mL) were randomly assigned in a 2:1 ratio to receive either GemCap with GV1001 (GV1001 group) or GemCap (control group).

Caption of figure 1:

Flow diagram of patient disposition. A total of 511 pancreatic adenocarcinoma patients were screened, of 148 patients were enrolled.

Patients with high serum eotaxin levels (>81.02 ng/mL) were randomly assigned in a 2:1 ratio to receive either Gemcitabine/Capecitabine with GV1001 (GV1001 group) or Gemcitabine/Capecitabine (control group). Finally, 148 patients were assigned to the GV1001 group (*n* = 75; all eotaxin-high) and control group (*n* = 73; 36 eotaxin-high and 37 eotaxin-low).

It should read:

Flow diagram of patient disposition. A total of 511 pancreatic adenocarcinoma patients were screened, of 148 patients were enrolled.

Patients with high serum eotaxin levels (>81.02 pg/mL) were randomly assigned in a 2:1 ratio to receive either Gemcitabine/Capecitabine with GV1001 (GV1001 group) or Gemcitabine/Capecitabine (control group). Finally, patients were assigned to the GV1001 group (n = 75; all eotaxin-high) and control group (n = 73; 36 eotaxin-high and 37 eotaxin-low).

The original article has been corrected.

