# Supplementary material for: Efficacy of GV1001 with gemcitabine/capecitabine in previously untreated patients with advanced pancreatic ductal adenocarcinoma having high serum eotaxin levels (KG4/2015): an open-label, randomised, Phase 3 trial
Source: Br J Cancer. 2023 Oct 30;130(1):43–52. doi: 10.1038/s41416-023-02474-w (PMC10781743; doi:10.1038/s41416-023-02474-w)
Supplement: Supplementary file 1 — Supplementary tables - revised [file 41416_2023_2474_MOESM1_ESM.docx]

**Supplementary Materials**

**Tables**

**Supplementary Table S1. The baseline characters of screened subjects by eotaxin groups**

|  | **Eotaxin High (N=174)** | **Eotaxin Low (N=328)** |
| --- | --- | --- |
| **Age (years)** |  |  |
| Mean | 64.9 | 63.4 |
| **Sex, n(%)** |  |  |
| Male | 94(54.02) | 185(56.40) |
| Female | 80(45.98) | 143(43.60) |
| **Smoking, n(%)** |  |  |
| Current | 24(13.80) | 47(14.33) |
| Past | 55(30.46) | 109(33.23) |
| No | 97(55.74) | 172(52.44) |
| **Alcohol, n(%)** |  |  |
| Current | 29(16.67) | 65(19.82) |
| Past | 45(25.86) | 98(29.88) |
| No | 100(57.47) | 165(50.30) |

Note: Total 511 subjects were screened, but among them 502 subjects checked their eotaxin level at baseline.

**Supplementary Table S2. Summary of events and censoring in survival analysis**

|  | **GV1001 group (n=75)** | **Control group (n=73)** | **P-value** |
| --- | --- | --- | --- |
| **Events (death) (%)** | 46/75 (61.3%) | 34/73 (46.6%) | 0.072 |
| **Censoring (%)** | 29/75 (38.7%) | 39/73 (53.4%) |  |
| **Cause of censoring (%)** |  |  |  |
| **Withdrawal** | 18 (24.0%) | 32 (43.8%) | 0.011 |
| **Follow-up loss** | 4 (5.3%) | 2 (2.7%) | 0.681 |
| **Survival** | 7 (9.3%) | 5 (6.8%) | 0.580 |

**Supplementary Table S3. Multivariate Cox proportional analysis of the contribution of serum eotaxin level to overall survival**

| **A. GV1001 group (n=75)** | |  |  |  |  |
| --- | --- | --- | --- | --- | --- |
|  | **Univariate** | |  | **Multivariate** | |
|  | **HR (95% CI)** | **P-value** |  | **HR (95% CI)** | **P-value** |
| **Eotaxin (ng/mL)** | 1.010 (0.998-1.023) | 0.106 |  | 1.012 (0.999-1.024) | 0.065 |
| **Age (yr)** | 1.001 (0.963-1.040) | 0.976 |  |  |  |
| **Male (vs. female)** | 1.021 (0.568-1.835) | 0.946 |  |  |  |
| **Metastatic disease  (vs. locally advanced)** | 2.468 (1.183-5.148) | 0.016 |  | 2.585 (1.235-5.410) | 0.012 |
| **B. Control group (n=73)** | |  |  |  |  |
|  | **Univariate** | |  | **Multivariate** | |
|  | **HR (95% CI)** | **P-value** |  | **HR (95% CI)** | **P-value** |
| **Eotaxin (ng/mL)** | 1.005 (0.995-1.015) | 0.293 |  | 1.007 (0.997-1.017) | 0.160 |
| **Age (yr)** | 1.010 (0.973-1.049) | 0.598 |  |  |  |
| **Male (vs. female)** | 1.058 (0.507-2.208) | 0.881 |  |  |  |
| **Metastatic disease  (vs. locally advanced)** | 2.202 (0.949-5.109) | 0.066 |  | 2.406 (1.022-5.664) | 0.044 |

CI, confidence interval

**Supplementary Table 4. Subgroup analyses for overall survival and time-to-progression**

1. **OS**

| **Subgroup** | **# of events / patients** | | **Survival, median (95%CI)** | | **p-value** |
| --- | --- | --- | --- | --- | --- |
|  | **GV1001** | **Control** | **GV1001** | **Control** |  |
| **Full** | 46/75 | 34/73 | 11.3 (8.6-14) | 7.5 (5.1-10) | 0.021 |
| **Sex** |  |  |  |  |  |
| **Male** | 21/34 | 22/46 | 11.7 (8.1-15.3) | 8.9 (4.9-12.9) | 0.149 |
| **Female** | 25/41 | 12/27 | 9.9 (5.7-14.2) | 6.1 (3.9-8.4) | 0.059 |
| **Age** |  |  |  |  |  |
| **≤65** | 25/42 | 22/46 | 11.4 (8-14.9) | 7.2 (3.9-10.4) | 0.036 |
| **>65** | 21/33 | 12/27 | 9.9 (5.7-14.2) | 8.1 (4.5-11.6) | 0.256 |
| **Tumor status** |  |  |  |  |  |
| **Locally Advanced** | 10/20 | 6/16 | 15.2 (7.1-23.3) | 10.9 (0.4-21.4) | 0.261 |
| **Metastasis** | 36/55 | 28/57 | 9.9 (7.4-12.5) | 5.8 (3.9-7.8) | 0.006 |
| **ECOG** |  |  |  |  |  |
| **0** | 25/45 | 19/44 | 11.3 (6.6-16) | 8 (5.1-11) | 0.127 |
| **1 or 2** | 21/30 | 15/29 | 11.1 (7.7-14.6) | 5.2 (1.6-8.7) | 0.010 |
| **Primary tumor location** |  |  |  |  |  |
| **Head** | 24/39 | 21/32 | 11.4 (8-14.7) | 8.5 (5.5-11.5) | 0.104 |
| **Others** | 22/36 | 13/41 | 11.1 (5.8-16.4) | 5.7 (2.4-9) | 0.045 |
| **CA 19-9** |  |  |  |  |  |
| **High ( > 379.9)** | 26/38 | 19/36 | 9.9 (7.2-12.7) | 7.2 (4.4-9.9) | 0.082 |
| **Low (≤ 379.9)** | 20/37 | 15/37 | 14.4 (7.8-21) | 8.9 (5.3-12.6) | 0.067 |

1. **TTP**

| **Subgroup** | **# of events / patients** | | **Survival, median (95%CI)** | | **p-value** |
| --- | --- | --- | --- | --- | --- |
|  | **GV1001** | **Control** | **GV1001** | **Control** |  |
| **Full** | 52/75 | 41/73 | 7.3 (5-9.7) | 4.5 (3.2-5.8) | 0.021 |
| **Sex** |  |  |  |  |  |
| **Male** | 26/34 | 26/46 | 7.4 (5.3-9.5) | 4.5 (2-7.1) | 0.045 |
| **Female** | 26/41 | 15/27 | 5.9 (2-9.8) | 4.5 (2.3-6.7) | 0.269 |
| **Age** |  |  |  |  |  |
| **≤65** | 28/42 | 26/46 | 7.4 (4.7-10.1) | 4.5 (2.2-6.8) | 0.055 |
| **>65** | 24/33 | 15/27 | 7.1 (3.1-11.1) | 4.6 (1.5-7.7) | 0.168 |
| **Tumor status** |  |  |  |  |  |
| **Locally Advanced** | 13/20 | 5/16 | 8.4 (5.6-11.2) | 9.4 (0.6-18.2) | 0.583 |
| **Metastasis** | 39/55 | 36/57 | 4.6 (2.2-7.1) | 4.5 (2.7-6.3) | 0.466 |
| **ECOG** |  |  |  |  |  |
| **0** | 29/45 | 21/44 | 7.4 (5-9.9) | 4.7 (2.3-7.1) | 0.058 |
| **1 or 2** | 23/30 | 20/29 | 5.8 (2.8-8.9) | 1.8 (0-4.1) | 0.019 |
| **Primary tumor location** |  |  |  |  |  |
| **Head** | 30/39 | 17/32 | 7.3 (4.9-9.8) | 4.7 (2-7.4) | 0.077 |
| **others** | 22/36 | 24/41 | 7.4 (3.9-10.9) | 4.5 (2.1-6.9) | 0.093 |
| **CA 19-9** |  |  |  |  |  |
| **High ( > 379.9)** | 26/38 | 22/36 | 4.6 (2-7.3) | 4.5 (3.1-5.9) | 0.470 |
| **Low (≤ 379.9)** | 26/37 | 19/37 | 7.4 (4.7-10.1) | 4.7 (1.3-8.1) | 0.097 |

**Supplementary Table S5. Frequent adverse events**

| **Most Frequent TEAE Occurred with > 2 Patients Each from Treatment and Control** | | | | |  |  |  |
| --- | --- | --- | --- | --- | --- | --- | --- |
|  |  | **Treatment** | | **Control** | | **Overall** | |
|  |  |  |  |  |  |  |  |
| **System Organ Class** | | **(n=75)** | | **(n=73)** | | **(n=148)** | |
|  | **Preferred Term** | **n (%)** | **events** | **n (%)** | **events** | **n (%)** | **events** |
| **Blood and lymphatic system disorders** | |  |  |  |  |  |  |
|  | Anemia | 20 (26.7) | 87 | 23 (31.5) | 83 | 43 (29.1) | 170 |
| **Gastrointestinal disorders** | |  |  |  |  |  |  |
|  | Nausea | 27 (36) | 39 | 17 (23.3) | 25 | 44 (29.7) | 64 |
|  | Abdominal pain | 16 (21.3) | 24 | 12 (16.4) | 16 | 28 (18.9) | 40 |
|  | Vomiting | 16 (21.3) | 22 | 10 (13.7) | 17 | 26 (17.6) | 39 |
|  | Constipation | 12 (16) | 15 | 15 (20.5) | 16 | 27 (18.2) | 31 |
|  | Diarrhoea | 11 (14.7) | 14 | 12 (16.4) | 15 | 23 (15.5) | 29 |
|  | Stomatitis | 7 (9.3) | 11 | 7 (9.6) | 9 | 14 (9.5) | 20 |
|  | Dyspepsia | 7 (9.3) | 7 | 10 (13.7) | 11 | 17 (11.5) | 18 |
|  | Abdominal pain upper | 6 (8) | 9 | 4 (5.5) | 5 | 10 (6.8) | 14 |
| **General disorders and administration site conditions** | |  |  |  |  |  |  |
|  | Asthenia | 17 (22.7) | 31 | 18 (24.7) | 33 | 35 (23.6) | 64 |
|  | Pyrexia | 21 (28) | 41 | 12 (16.4) | 18 | 33 (22.3) | 59 |
|  | Oedema peripheral | 7 (9.3) | 11 | 6 (8.2) | 6 | 13 (8.8) | 17 |
|  | Chills | 6 (8) | 10 | 3 (4.1) | 3 | 9 (6.1) | 13 |
|  | Oedema | 4 (5.3) | 4 | 3 (4.1) | 5 | 7 (4.7) | 9 |
|  | Chest pain | 4 (5.3) | 4 | 3 (4.1) | 4 | 7 (4.7) | 8 |
| **Investigations** | |  |  |  |  |  |  |
|  | Platelet count decreased | 43 (57.3) | 201 | 35 (47.9) | 161 | 78 (52.7) | 362 |
|  | Neutrophil count decreased | 44 (58.7) | 158 | 35 (47.9) | 159 | 79 (53.4) | 317 |
|  | White blood cell count decreased | 9 (12) | 19 | 11 (15.1) | 37 | 20 (13.5) | 56 |
|  | Weight decreased | 9 (12) | 17 | 7 (9.6) | 24 | 16 (10.8) | 41 |
|  | Weight increased | 5 (6.7) | 15 | 5 (6.8) | 12 | 10 (6.8) | 27 |
|  | Aspartate aminotransferase increased | 4 (5.3) | 4 | 7 (9.6) | 15 | 11 (7.4) | 19 |
|  | Creatinine renal clearance decreased | 6 (8) | 11 | 3 (4.1) | 4 | 9 (6.1) | 15 |
|  | Alanine aminotransferase increased | 4 (5.3) | 4 | 6 (8.2) | 9 | 10 (6.8) | 13 |
| **Metabolism and nutrition disorders** | |  |  |  |  |  |  |
|  | Decreased appetite | 13 (17.3) | 17 | 16 (21.9) | 18 | 29 (19.6) | 35 |
|  | Hypoglycaemia | 3 (4) | 5 | 3 (4.1) | 3 | 6 (4.1) | 8 |
| **Musculoskeletal and connective tissue disorders** | |  |  |  |  |  |  |
|  | Back pain | 6 (8) | 9 | 4 (5.5) | 4 | 10 (6.8) | 13 |
|  | Pain in extremity | 3 (4) | 3 | 3 (4.1) | 3 | 6 (4.1) | 6 |
| **Nervous system disorders** | |  |  |  |  |  |  |
|  | Headache | 6 (8) | 8 | 5 (6.8) | 7 | 11 (7.4) | 15 |
|  | Dizziness | 5 (6.7) | 6 | 6 (8.2) | 8 | 11 (7.4) | 14 |
| **Psychiatric disorders** | |  |  |  |  |  |  |
|  | Insomnia | 6 (8) | 6 | 5 (6.8) | 5 | 11 (7.4) | 11 |
| **Respiratory, thoracic and mediastinal disorders** | |  |  |  |  |  |  |
|  | Dyspnoea | 5 (6.7) | 6 | 5 (6.8) | 6 | 10 (6.8) | 12 |
| **Skin and subcutaneous tissue disorders** | |  |  |  |  |  |  |
|  | Palmar-plantar erythrodysaesthesia syndrome | 12 (16) | 19 | 15 (20.5) | 24 | 27 (18.2) | 43 |
|  | Pruritus | 23 (30.7) | 31 | 8 (11) | 11 | 31 (20.9) | 42 |
|  | Urticaria | 13 (17.3) | 15 | 4 (5.5) | 4 | 17 (11.5) | 19 |

**Supplementary Table S6. Protein marker candidates to predict the response for GV1001**

| Gene symbol | Antibody name | GV1001 group | | Control group | | Average of normalized data (log2) | | | |
| --- | --- | --- | --- | --- | --- | --- | --- | --- | --- |
|  |  | FC | P-value | FC | P-value | TP | TG | CP | CG |
| NGF | NGF beta | 2.836 | 0.000 | 0.577 | 0.077 | 10.112 | 11.616 | 12.729 | 11.936 |
| MMP2 | MMP-2 | 1.734 | 0.001 | 0.915 | 0.552 | 10.801 | 11.594 | 11.797 | 11.668 |
| NRG1 | Heregulin | 1.465 | 0.001 | 0.815 | 0.213 | 10.236 | 10.787 | 11.011 | 10.716 |
| VEGFB | VEGFB | 1.430 | 0.001 | 0.917 | 0.613 | 9.461 | 9.977 | 10.009 | 9.885 |
| MMP10 | MMP-10 | 1.372 | 0.007 | 1.022 | 0.912 | 11.057 | 11.514 | 11.638 | 11.669 |
| CTNNA1 | Catenin-alpha1 | 1.341 | 0.022 | 0.964 | 0.818 | 10.054 | 10.478 | 10.463 | 10.409 |
| TNFRSF1B | TNF-receptor II | 1.341 | 0.049 | 0.879 | 0.104 | 8.183 | 8.606 | 8.395 | 8.208 |
| IGF2 | IGF-II | 1.333 | 0.002 | 0.757 | 0.081 | 8.704 | 9.119 | 9.254 | 8.852 |
| JUP | Catenin-gamma | 1.305 | 0.012 | 0.986 | 0.923 | 9.194 | 9.577 | 9.623 | 9.602 |
| LTA | TNF-beta | 1.276 | 0.029 | 0.933 | 0.420 | 8.036 | 8.388 | 8.233 | 8.133 |

**Supplementary Table S7. Multivariate Cox proportional analysis of the contribution of an elevated expression of protein marker candidates to survival**

| Gene | Antibody name | Overall Survival | | Progression-Free Survival | |
| --- | --- | --- | --- | --- | --- |
|  |  | HR (95% CI) | P-value† | HR (95% CI) | P-value† |
| *NGF* | NGF beta | 0.61 (0.30–1.26) | 0.180 | 0.60 (0.29–1.26) | 0.177 |
| *MMP2* | MMP-2 | 0.37 (0.18–0.76) | 0.007 | 1.02 (0.28–3.65) | 0.981 |
| *NRG1* | Heregulin | 0.57 (0.24–1.33) | 0.194 | 0.76 (0.38–1.51) | 0.436 |
| *VEGFB* | VEGFB | 1.59 (0.62–4.09) | 0.341 | 0.97 (0.39–2.46) | 0.954 |
| *MMP10* | MMP-10 | 1.41 (0.51–3.91) | 0.504 | 0.80 (0.41–1.58) | 0.530 |
| *CTNNA1* | Catenin-alpha1 | 0.42 (0.10–1.74) | 0.230 | 0.37 (0.20–0.68) | 0.002* |
| *TNFRSF1B* | sTNF-receptor II | 0.69 (0.20–2.38) | 0.558 | 0.29 (0.09–0.91) | 0.034 |
| *IGF2* | IGF-II | 1.88 (0.71–4.98) | 0.208 | 1.13 (0.38–3.36) | 0.825 |
| *JUP* | Catenin-gamma | 1.83 (0.76–4.38) | 0.178 | 1.51 (0.67–3.40)) | 0.322 |
| *LTA* | TNF-beta | 1.26 (0.33–4.75) | 0.732 | 3.22 (1.03–10.04) | 0.044 |

†The Bonferroni Method was used to determine the family-wise error rate for multiple testing; p-value <0.005 was deemed to indicate statistical significance.
