# Supplementary material for: Efficacy of GV1001 with gemcitabine/capecitabine in previously untreated patients with advanced pancreatic ductal adenocarcinoma having high serum eotaxin levels (KG4/2015): an open-label, randomised, Phase 3 trial
Source: Br J Cancer. 2023 Oct 30;130(1):43–52. doi: 10.1038/s41416-023-02474-w (PMC10781743; doi:10.1038/s41416-023-02474-w)
Supplement: Supplementary file 2 — Supplementary appendix1_KG4-2014_Study protocol [file 41416_2023_2474_MOESM2_ESM.docx]

**[Supplemetary Appendix 1]**

| **P R O T O C O L** |
| --- |

**A prospective, randomized, open-label, multicenter, parallel design, phase III study to assess the efficacy and safety of GV1001 concurrent with Gemcitabine/Capecitabine versus Gemcitabine/Capecitabine alone in treating locally advanced and metastatic pancreatic cancer patients**

| **Protocol No.:** | **KG 4/2015** |
| --- | --- |
| **Version No.:** | **2.1** |
| **Issue Date:** | **2017.10.16** |
| **Coordinating Investigator** | **Professor Si-Young Song, Severance Hospital** |

| **Confidentiality Statement**  This document and the information contained therein are the exclusive property of Samsung Pharm. Ltd. and may not be changed or disclosed without the written consent of the company. |
| --- |

#

# SYNOPSIS

| Name of Sponsor | Samsung Pharm. Ltd. |
| --- | --- |
| Name of Finished Product | Riavax Ing. (GV1001) |
| Name of Active Ingredient | Tertomitide hydrochloride |
| Title of study | A prospective, randomized, open-label, multicenter, parallel design, phase III study to assess the efficacy and safety of GV1001 concurrent with Gemcitabine/Capecitabine versus Gemcitabine/Capecitabine alone in treating locally advanced and metastatic pancreatic cancer patients |
| Protocol No. | KG 4/2015 |
| Coordinating Investigator | Professor Si-Young Song, Division of Gastroenterology, Severance Hospital |
| Institutions | Severance Hospital and 15 other sites in South Korea (a total of 16 sites) |
| Study period | 54 months from IRB approval of the protocol (Enrollment period 42 months, Treatment/Follow-up 12 months) |
| Indication | Locally advanced and metastatic pancreatic cancer |
| Phase | Phase III |
| Study design | Randomized, open-label, multicenter, parallel design |
| Study objectives | **Primary objective:**  To compare overall survival (OS) between the two treatment groups (the GV1001+gemcitabine/capecitabine study group versus the gemcitabine/capecitabine control group) in patients with locally advanced and metastatic pancreatic cancer  **Secondary objectives:**  To compare the following efficacy and safety endpoints between the two treatment groups (the GV1001+gemcitabine/capecitabine study group versus the gemcitabine/capecitabine control group):   1. Time to tumor progression (TTP) 2. Objective response rate (ORR) 3. Clinical benefit response (CBR) 4. Assessment of correlation between eotaxin level and treatment response 5. Quality of life (QoL) 6. Change in CA19-9 over time 7. Safety and tolerability   **Exploratory objectives:**   1. Assessment of immune response  - T-cell proliferation - Delayed type hypersensitivity (DTH) |
| Dosing schedule, dose, and method of administration | **Dosing schedule**   1. GV1001 (0.56 mg)+GM-CSF (75 mcg)   One ID dose of GV1001 and GM-CSF was given on Days 1, 3, and 5 of Week 1 followed by one ID dose at Weeks 2, 3, 4, and 6. Afterwards, therapy was repeated every 4 weeks until it was discontinued due to the subject’s request, occurrence of intolerable toxicity, or progressive disease (PD).   1. Gemcitabine/capecitabine   One cycle of treatment was defined as follows. Therapy was repeated until the subject’s request, occurrence of intolerable toxicity, or PD.  - Gemcitabine: once weekly intravenous (IV) doses for 3 consecutive weeks (Days 1, 8, and 15) followed by 1 week off treatment  - Capecitabine: twice daily (morning/evening) oral doses for 21 days followed by 7 days off treatment  **Dose/method of administration**   1. GV1001 (0.56 mg)+GM-CSF (75 mcg) 2. The adjuvant GM-CSF (Leukine®) 0.15 mL (75 mcg, 1.4x106 IU/vial, 0.5 mg/mL) was intradermally (ID) administered to the lower abdomen, and between 10 and 15 minutes after dosing, GV1001 0.20 mL (0.56 mg, 2.8 mg/mL) was administered as an ID injection at the same site of administration. Dosing schedules of GV1001 and the adjuvant GM-CSF were not affected by dosing sequences and time intervals with gemcitabine/capecitabine therapy. 3. Gemcitabine 4. Gemcitabine 1,000 mg/m2 was intravenously administered over ≥30 minutes. 5. Capecitabine   Capecitabine 830 mg/m2 was orally administered twice daily (morning, evening) with water within 30 minutes after a meal (1,660 mg/m2 per day). |
| Number of Subjects | 148 subjects are planned to be enrolled  (1) Study group (GV1001 + gemcitabine/capecitabine): 74 subjects  (2) Control group (gemcitabine/capecitabine): 74 subjects |
| Inclusion criteriae | 1. Age of ≥ 19 years 2. Histopathologically or cytologically proven pancreatic ductal adenocarcinoma or undifferentiated carcinoma of the pancreas 3. Locally advanced or metastatic disease precluding curative surgical resection or patients who have relapsed following previously resected pancreatic cancer; the patient must have measurable lesions. 4. Contrast enhanced CT scans on the thorax, abdomen, and pelvis within 28 days (and up to a maximum of 32 days) prior to commencing IP treatment (patients who are unable to receive contrast due to allergies etc. may still be eligible for this trial if it is possible to perform tumor assessment based on non-contrast enhanced CT scans.)  * Those who cannot undergo CT scanning may be tested by MRI. ** Images (e.g., positron emission tomography-CT, etc.) obtained within the relevant period may be used if available. 5. Measurable lesions on the screening CT scan (or MRI) according to RECIST guideline (see Protocol Appendix 4. RECIST guideline v1.1) 6. Eastern Cooperative Oncology Group (ECOG) performance status 0, 1, or 2 (see Protocol Appendix 2. ECOG Performance Status) 7. Adequate organ function as determined by pre-enrollment clinical laboratory tests:  - Platelets ≥ 100 x 10^9^/L - WBC ≥ 3 x 10^9^/L - ANC ≥ 1.5 x 10^9^/L - Serum total bilirubin ≤ 2.0 mg/dL - CCr (Cockcroft&Gault) > 50 mL/min  1. Life expectancy ≥ 90 days 2. The subject or his/her legally acceptable representative must provide written informed consent prior to study initiation, and the subject must be capable of complying with study requirements. |
| Exclusion criteria | 1. Brain metastasis or meningeal carcinomatosis 2. Clinically significant serious disease or organ system disease not currently controlled on present therapy 3. Previous chemotherapy for locally advanced and metastatic disease. (However, previous adjuvant chemotherapy for resected pancreatic cancer are permitted providing chemotherapy was completed more than 12 months previously prior to enrollment.) 4. Radiotherapy within the last 8 weeks prior to enrollment 5. Concurrent malignancies or invasive cancer diagnosed within 5 years prior to enrollment (except for treated Basal Cell Carcinoma of the skin, in situ carcinoma of the uterine cervix or resected pancreatic cancer) 6. Medication which might affect immunocompetence e.g. chronic treatment with long term steroids or other immunosuppressant. Patients are eligible if they have been receiving short term steroids for palliation of cancer related symptoms. 7. Having participated in another clinical trial to be treated with another IP within 8 weeks prior to enrollment. 8. Pregnancy or breast feeding 9. Unstable angina pectoris 10. Known malabsorption syndromes 11. Patients with a known hypersensitivity reactions to the active ingredient and any of the excipients of the IP or patients with a dihydropyrimidine dehydrogenase deficiency 12. All men or women of reproductive potential, unless using at least two contraceptive precautions, one of which must be a condom 13. Patients considered by the investigator to be ineligible for this study |
| Precautions for concomitant use and prohibited drugs/treatment | **Drugs/treatment for concomitant use with precautions**   - Anticoagulants (e.g., warfarin): requires regular monitoring for blood coagulation factors (prothrombin time or international normalized ratio). - Phenytoin: requires regular monitoring for serum phenytoin concentration. - Metronidazole (caution should be exercised for potential interactions with capecitabine.)   **Prohibited drugs/treatment**   - Drugs and treatment that may affect the efficacy of the IP such as anticancer treatment and radiotherapy (immunosuppressants, other IPs, other anticancer chemotherapy/radiotherapy) - Dipyridamole, allopurinol - Continuous systemic corticosteroids (short-term treatment with low-dose systemic corticosteroids may be allowed at the discretion of the investigator.) |
| Concomitant treatment | During the treatment period, concomitant therapy for any disease other than pancreatic cancer was allowed, and concomitant drugs should be written in eCRF.  If surgery was inevitably needed, GV1001 treatment was temporarily interrupted until the outcome of the surgery was known. |
| Method | This study was designed as a randomized, open-label, multicenter, parallel-design Phase 3 clinical trial to evaluate the efficacy and safety of GV1001 in combination with gemcitabine/capecitabine versus gemcitabine/capecitabine in patients with locally advanced and metastatic pancreatic cancer.  Subjects eligible for study participation were classified into 2 groups, high eotaxin (>81.02 pg/mL) versus low eotaxin (≤81.02 pg/mL), based on the eotaxin test. Those in the high eotaxin group were randomized to either the study group (GV1001 plus gemcitabine/capecitabine) or the control group (gemcitabine/capecitabine) in a 2:1 ratio while those in the low eotaxin group were allocated to the control group in the same ratio as the control group of the high eotaxin group. In the high eotaxin group, subjects were stratified by progression of pancreatic cancer (locally advanced, metastatic) for randomization. Those in the low eotaxin group were not randomized; they were stratified by progression of pancreatic cancer (locally advanced, metastatic) and enrolled in the control group in the same ratio as the control group of the high eotaxin group.  **Screening (Eotaxin testing)**  Eotaxin Low group  (≤81.02 pg/mL)  Eotaxin High group  (>81.02 pg/mL)  **Randomization (2:1)**  **Study group**  **(Gem/Cap+GV1001/GM-CSF)**  **Control group (Gem/Cap)**  **[Eotaxin High : Low ratio =1:1**]  Eligibility for study participation was assessed within 21 days prior to investigational product (IP) dosing. Subjects who were determined eligible and enrolled in the study were treated with the IP and assessed for efficacy and safety as planned. All subjects continued to be treated with the IP until progressive disease (PD) was determined by computed tomography (CT) scan (or magnetic resonance imaging [MRI]) and were followed up until death or the end of study (EOS).  To determine PD of pancreatic cancer, CT scanning (or MRI) was performed every 12 weeks from Week 8, and tumor response was assessed according to Response Evaluation Criteria In Solid Tumors (RECIST) and Immune-Related Response Criteria (irRC). The safety and tolerability of the IP were assessed based on vital signs, clinical laboratory tests, adverse events (AEs), etc.  All subjects who completed IP dosing or who were prematurely withdrawn from the study (except for those who withdrew their informed consent) were followed up for survival every 12 weeks until death or EOS.  EOS was defined as any of the following 3 time points: 1) the last subject completed 52 weeks of treatment and/or follow-up, 2) the last on-going patient had been subject to treatment and/or follow-up for at least 52 weeks, or 3) all subjects were discontinued. Therefore, the last subject who was applicable to 1) or 2) underwent CT scanning (or MRI) for tumor assessment at Week 52. |
| Endpoints and statistical methods | **Primary efficacy endpoint:**  Overall Survival, OS  OS was defined as time from date of randomization to date of death from any cause. OS was assessed at EOS.  **Secondaary efficacy endpoints:**   1. Time to tumor progression (TTP)   TTP was defined as the time from the date of randomization to the date of progressive disease as measured by CT. Methods of analysis were the same as in the primary efficacy endpoint.   1. Objective response rate (by RECIST v1.1 and irRC)   The number of subjects with complete response (CR) or partial response (PR) based on CT (or MRI) according to RECIST and irRC   1. Clinical benefit response (CBR)   The number of subjects assessed as clinical benefit responders   1. Assessment of correlation between eotaxin level and treatment response 2. Change from baseline in QoL (EORTC QLQ-C30, EQ-5D) scores   Change from baseline in QoL (EORTC QLQ-C30 v3.0, EQ-5D-3L) score at every points (Week 3, 8, 20, 32, and every 12 weeks thereafter)   1. Change in CA19-9 over time 2. Safety   Adverse events (NCI CTCAE v4.03), vital sign, physical exam, lab test, weight, ECOG performance  **Exploratory efficacy endpoints:**   1. Assessment of immune response   - T-cell proliferation  *Determination of positive T-cell proliferation:  (Method 1) ^3^H-thymidine uptake assay  Using the measured T-cell (count per minutes [c.p.m.]) value, *Stimulatory Index (SI) was calculated; the test was determined positive if SI was ≥2.  *Stimulatory Index (SI) = c.p.m. (test result at each testing time point after initiation of GV1001 dosing) / c.p.m. (test result before GV1001 dosing at the baseline visit)  (Method 2) CFSE assay  In comparison with the value obtained before the first dose of GV1001 at the baseline visit (Visit 3), T cell proliferation measured at pre-defined post-dose time points was calculated as *SI; The test was determined positive if SI was ≥2 OR ** difference in the number of T cell divisions was ≥1.  *Stimulatory Index (SI) = ***T cell population at the testing time point after initiation of GV1001 dosing/***T cell population before GV1001 dosing at the baseline visit  ** Difference in the number of T cell divisions = number of T cell divisions at the testing time point after initiation of GV1001 dosing - number of T cell divisions before GV1001 dosing at the baseline visit  ***T cell population refers to the number of T cells left-shifted from the undifferentiated parent peak on the CFSE content analysis by flow cytometer; cell number can be represented as % by gating.  - Delayed-type hypersensitivity (DTH*) testing  * Positive DTH: The test was determined “positive” if a red spot occurring 48 hours post-dose was no smaller than 5 mm * 5 mm (mean ≥5 mm, 5+5/2).  If it was difficult to determine the size, a spot with the smallest diameter of ≥3 mm was determined positive (e.g., positive for 3 mm * 7 mm; negative for 2 mm * 8 mm).  **Subgroup analysis**   1. Survival and objective response rate by DTH or T-cell proliferation   Subjects were categorized as ‘responders’ for those with positive DTH or T-cell proliferation outcomes versus ‘non-responders’ for the rest of the subjects; by category, OS and ORR were assessed in the same manner as above.   1. Change in CA19-9 by tumor response   Subjects with complete response (CR) or partial response (PR) were classified as ‘responders’ and assessed for change in CA19-9. |
| Statistical Analysis Method | **Primary efficacy endpoint:**  To compare the study group and the control group, satisfaction of the proportional hazard assumption was examined: the stratified log-rank test was used if the model satisfied the assumption; and the stratified generalized Wilcoxon test (Gehan test) was used if not. By group, 25%, 50%, and 75% quantile estimates and their 95% confidence intervals (CIs) as well as Kaplan-Meier curves were presented. An analysis was performed using the Cox PH model with progression of pancreatic cancer as a covariate, and the estimated hazard ratio and its 95% CI were presented.  **Secondary efficacy endpoints:**   1. Time to tumor progression; TTP   Methods of analysis were the same as in the primary efficacy endpoint.   1. Objective response rate (by RECIST and irRC)   The number of subjects with complete response (CR) or partial response (PR) based on CT (or MRI) according to RECIST and irRC as well as percentage and its 95% CI were presented for each group, and Chi-square test or Fisher’s exact test was performed for the difference between treatment groups.   1. Clinical benefit response   The number of subjects assessed as clinical benefit responders as well as percentage and its 95% CI were presented for each group, and Chi-square test or Fisher’s exact test was performed for the difference between treatment groups.   1. Assessment of correlation between eotaxin level and treatment response   Comparison of OS by eotaxin level (high, low) within the control group was analyzed using the same statistical methods as in the primary efficacy assessment. Additionally, comparison of OS between the study group and the control group among those with high eotaxin level was analyzed using the same statistical methods as in the primary efficacy assessment.   1. Change from baseline in QoL (EORTC QLQ-C30, EQ-5D) scores   Descriptive statistics were provided for scores for each group. Two sample t-test or Wilcoxon rank sum test was performed for change from baseline in scores between treatment groups.   1. Change in CA19-9 over time   For change from baseline in CA19-9 between treatment groups was analyzed using Mixed model repeated measures (MMRM).   1. Safety   The analysis of adverse events was performed using treatment-emergent adverse events (TEAEs). AEs were coded by Medical Dictionary for Regulatory Activities 23.0, and severity of AEs was classified according to National Cancer Institute Common Toxicity Criteria Adverse Event (NCI CTCAE) 4.03. By treatment group, percentage of subjects with AEs and its 95% CI were presented. All AEs were summarized by severity. In addition, AEs related to the IP and serious AEs (SAEs) were separately summarized. The incidence of AEs between treatment groups was analyzed by Chi-square test or Fisher’s exact test. Percentage of subjects with Grade 3 or 4 AEs based on NCI CTCAE and its 95% CI were presented, and the difference between treatment groups was analyzed by Chi-square test or Fisher’s exact test.  **Exploratory efficacy endpoints:**  (1) Assessment of immune response:  The number of subjects with positive T-cell proliferation or DTH outcomes, percentage and its 95% CI were presented.   1. T-cell proliferation 2. Delayed-type hypersensitivity (DTH) test   **Subgroup analysis**   1. Survival and objective response rate by DTH test or T-cell proliferation   Subjects were categorized as ‘responders’ for those with positive DTH or T-cell proliferation results versus ‘non-responders’ for the rest of the subjects; by category, OS and ORR were assessed. A subgroup analysis was performed on OS and proportion of CR and PR only amongst those with positive T-cell proliferation or DTH results. The same analytical method used for the relevant variable was applied.   1. Change in CA19-9 by tumor response   Subjects with CR or PR were classified as ‘responders’ and assessed for change in CA19-9. Amongst those who had CR or PR in tumor assessment, change in CA19-9 since they had a response was compared between treatment groups using MMRM. |

# LIST OF ABBREVIATIONS AND DEFINITION OF TERMS

| Abbreviation | Description |
| --- | --- |
| 5-FU | 5-Fluorouracil |
| ALT | Alanine Amino Transferase |
| ANC | Absolute Neutrophil Count |
| anti-HCV | Antihepatitis C Virus |
| ARDS | [Adult Respiratory Distress Syndrome](http://terms.naver.com/ncrEntry.nhn?ncrDocId=AA000602&dicId=disease_medicine#TABLE_OF_CONTENT1) |
| AST | Aspartate Amino Transferase |
| ATC | Anatomical Therapeutic Chemical |
| BUN | Blood Urea Nitrogen |
| Ca | Calcium |
| CA19-9 | Carbohydrate antigen 19-9 |
| CBR | Clinical Benefit response |
| CCr | Creatinine clearance rate |
| CFR | Code of Federal Regulations |
| CNS | Central Nervous System |
| CR | Complete Response |
| CRA | Clinical Research Associate |
| CRC | Clinical Research Coordinator |
| CRP | C-Reactive Protein |
| CT | Computed Tomography |
| DNA | Deoxyribonucleic Acid |
| DTH | Delayed Type Hypersensitivity |
| ECG | Electrocardiography |
| ECOG | Eastern Cooperative Oncology Group Performance Status Scale |
| eCRF | Electronic Case Report Form |
| EDC | Electronic Data Capture system |
| EGFR | Epidermal Growth Factor Receptor |
| EORTC QLQ-C30 | European Oraganization for Research and Treatment of Cancer Quality of Life Questionnaire-C30 |
| EOS | End of Study |
| EOT | End of Treatment |
| EQ-5D | EuroQoL Five Dimensions Questionnaire-3L |
| ERCP | Endoscopic Retrograde CholangioPancreatography |
| FAS | Full Analysis Set |
| FDA | Food and Drug Administration |
| GCP | Good Clinical Practice |
| GM-CSF | Granulocyte Monocyte-Colony Stimulating Factor |
| Hb | Hemoglobin |
| HBsAg | Hepatitis B Surface Antigen |
| HCG | Human Chorionic Gonadotropin |
| Hct | Hematocrit |
| HIV | Human Immunodeficiency Virus |
| hTERT | human Telomerase Reverse Transcriptase |
| ICH | International Conference on Harmonisation |
| IDMC | Independent Data Monitoring Committee |
| INR | International Normalized Ratio |
| IRB | Institutional Review Board |
| irRC | Immune-Related Response Criteria |
| IWRS | Interactive Web Response System |
| K | Potassium |
| KGCP | Korea Good Clinical Practice |
| LOCF | Last Observation Carried Forward |
| MDSC | Myeloid-Derived Suppressor Cells |
| MedDRA | Medical Dictionary for Regulatory Activities |
| MRI | Magnetic Resonance Imaging |
| Na | Sodium |
| NCI CTCAE | National Cancer Institute Common Toxicity Criteria Adverse Event |
| NSCLC | Non-Small Cell Lung Cancer |
| OS | Overall Survival |
| P | Phosphorus |
| PBMC | Peripheral Blood Mononuclear Cell |
| PD | Progressive Disease |
| PET-CT scan | Positron Emission Tomography-computed Tomography scan |
| PPS | Per Protocol Set |
| PR | Partial Response |
| PT | Preferred Term |
| QoL | Quality of Life |
| RBC | Red Blood Cell |
| RECIST | Response Evaluation Criteria In Solid Tumors |
| SAE | Serious Adverse Event |
| SAS | Statistical Analysis System |
| SD | Stable Disease |
| SOC | System Organ Class |
| SOP | Standard Operating Procedure |
| SST | Serum Separator Tube |
| TEAE | Treatment Emergent Adverse Event |
| TTP | Time To tumor Progression |
| VAS | Visual Analogue Scale |
| WBC | White Blood Cell |
| WHO | World Health Organization |
| γ-GT | Gamma Glutamyl Transferase |

| **LIST OF CONTENTS** |
| --- |

[SYNOPSIS 2](#_Toc84601729)

[LIST OF ABBREVIATIONS AND DEFINITION OF TERMS 9](#_Toc84601730)

[1 TITLE AND PHASE OF STUDY 16](#_Toc84601731)

[1.1 Title 16](#_Toc84601732)

[1.2 Phase 16](#_Toc84601734)

[2 INSTITUTIONS AND ADDRESS 16](#_Toc84601735)

[3 PPRINCIPAL INVESTIGATORS AND INSTITUTIONS 16](#_Toc84601736)

[3.1 Sub-investigators and Pharmacists 16](#_Toc84601737)

[4 NAME AND ADDRESS OF SPONSOR 16](#_Toc84601738)

[4.1 Name and Address of Sponsor 16](#_Toc84601739)

[5 INTRODUCTION 17](#_Toc84601740)

[5.1 Locally Advanced and Metastatic Pancreatic Cancer 17](#_Toc84601741)

[5.2 Clinical Trials of GV1001 18](#_Toc84601743)

[5.3 Mechanism of Immune Vaccine GV1001 20](#_Toc84601744)

[5.4 Adjuvant GM-CSF 20](#_Toc84601746)

[5.5 Rationale for New Response Criteria for Immunotherapy 21](#_Toc84601747)

[6 STUDY OBJECTIVES 23](#_Toc84601748)

[6.1 Primary Objective 23](#_Toc84601749)

[6.2 Secondary Objective 23](#_Toc84601750)

[6.3 Exploratory Objectives 23](#_Toc84601759)

[7 INVESTIGATIONAL PRODUCT 23](#_Toc84601760)

[7.1 Identity of Investigational Product 23](#_Toc84601761)

[7.1.1 GV1001 23](#_Toc84601762)

[7.1.2 Adjuvant GM-CSH 24](#_Toc84601763)

[7.1.3 Gemcitabine 24](#_Toc84601792)

[7.1.4 Capecitabine 25](#_Toc84601793)

[7.2 Investigational Product Labeling 25](#_Toc84601809)

[7.3 Supply, Storage, and Management of the Investigational Product 25](#_Toc84601817)

[8 INDICATION 26](#_Toc84601820)

[9 INCLUSION/EXCLUSION CRITERIA, PLANNED NUMVER OF SUBJECTS AND THE RATIONALE 26](#_Toc84601821)

[9.1 Inclusion Criteria 26](#_Toc84601822)

[9.2 Exclusion Criteria 27](#_Toc84601840)

[9.3 Determination of Sample Size 27](#_Toc84601855)

[10 DURATION OF STUDY 28](#_Toc84601856)

[11 INVESTIGATIONAL PLAN 29](#_Toc84601858)

[11.1 Overall Study Design and Plan 29](#_Toc84601859)

[11.2 Treatment Breaks 33](#_Toc84601906)

[11.2.1 Simple Treatment Breaks 33](#_Toc84601907)

[11.2.2 Requiring Other Treatment 33](#_Toc84601915)

[11.3 Dose, Method and Duration of Administration 34](#_Toc84601916)

[11.3.1 GV1001 and the Adjuvant GM-CSF 34](#_Toc84601917)

[11.3.2 Capecitabine 35](#_Toc84601919)

[11.4 Selection of Doses in the Study 37](#_Toc84601922)

[11.5 Rational for the Duration of IP Treatment 37](#_Toc84601924)

[11.6 Concomitant Treatment 38](#_Toc84601926)

[11.6.1 Precautions for Concomitant Use and Prohibited Drugs/Treatment 38](#_Toc84601927)

[11.7 Method of Assigning Subjects to Treatment Groups 38](#_Toc84601928)

[11.8 Subject Enrollment Center 39](#_Toc84601941)

[11.9 Blinding 39](#_Toc84601942)

[12 EFFICACY AND SAFETY VARIABLES 40](#_Toc84601943)

[12.1 Schedules of Study Procedures 40](#_Toc84601944)

[12.2 Observations 50](#_Toc84601945)

[12.2.1 Obtain Informed Consent and Assign a Screening Number 50](#_Toc84601946)

[12.2.2 Demographic Data 50](#_Toc84601947)

[12.2.3 Histopathological/Cytological Diagnostic Information 50](#_Toc84601949)

[12.2.4 Medical, Surgical, and Treatment History of Pancreatic Cancer 50](#_Toc84601950)

[12.2.5 Drug Administration History 50](#_Toc84601952)

[12.2.6 Chest, Abdomen and Pelvis CT scan (or MRI) 51](#_Toc84601953)

[12.2.7 Immune Response Assessment 51](#_Toc84601955)

[12.2.8 QoL Assessment 52](#_Toc84601957)

[12.2.9 Pain Intensity Assessment 53](#_Toc84601958)

[12.2.10 Concomitant Drug 53](#_Toc84601960)

[12.2.11 Adverse Events 53](#_Toc84601961)

[12.2.12 Clinical Laboratory Tests 53](#_Toc84601962)

[12.2.13 Vital Signs 54](#_Toc84601975)

[12.2.14 Physical Examination 54](#_Toc84601976)

[12.2.15 Height and Weight 54](#_Toc84601979)

[12.2.16 ECOG Performance status 55](#_Toc84601980)

[12.2.17 ECG 55](#_Toc84601982)

[12.2.18 Pregnancy Test 55](#_Toc84601983)

[12.2.19 Randomization 55](#_Toc84601985)

[12.2.20 Investigational Product Prescription 55](#_Toc84601987)

[12.2.21 Compliance Check 55](#_Toc84601988)

[12.2.22 End of Treatment (EOT) and End of Study (EOS) 55](#_Toc84601989)

[12.2.23 Unscheduled visit 55](#_Toc84601990)

[12.2.24 Follow-up 56](#_Toc84601992)

[12.3 Detailed Schedule for the Trial 56](#_Toc84601993)

[13 EXPECTED ADVERSER EVENTS AND CAUTION FOR USE 56](#_Toc84601994)

[13.1 GV1001 56](#_Toc84601995)

[13.1.1 Expected Adverse Events 56](#_Toc84601996)

[13.1.2 Contradictions to Administration 59](#_Toc84601997)

[13.1.3 Dosing with Caution 59](#_Toc84601998)

[13.2 GM-CSF 60](#_Toc84601999)

[13.2.1 Expected Adverse Events 60](#_Toc84602000)

[13.2.2 Prohibition of Administration 60](#_Toc84602001)

[13.2.3 Careful Administration 60](#_Toc84602002)

[13.3 Gemcitabine 61](#_Toc84602003)

[13.4 Capecitabine 61](#_Toc84602004)

[14 CRITERIA FOR STUDY DISCONTINUATION AND SUBJECT WITHDRAWL 61](#_Toc84602005)

[14.1 Study Discontinuation 61](#_Toc84602006)

[14.2 Withdrawal Criteria 61](#_Toc84602007)

[14.3 Handling of Premature Withdrawal 62](#_Toc84602026)

[14.4 Replacement of Subjects 62](#_Toc84602030)

[14.5 Post-EOT/EOS Treatment 62](#_Toc84602032)

[15 STATISTICAL METHODS 62](#_Toc84602034)

[15.1 Criteria and Methods for Assessment 62](#_Toc84602035)

[15.1.1 Primary Efficacy Endpoint 62](#_Toc84602036)

[15.1.2 Secondary Efficacy Endpoint 62](#_Toc84602038)

[15.1.3 Exploratory Efficacy Endpoints 63](#_Toc84602039)

[15.2 Statistical Methods 64](#_Toc84602040)

[15.2.1 General Principles for Analysis of Results 64](#_Toc84602041)

[15.2.2 Efficacy Set 65](#_Toc84602042)

[15.2.3 Demographic Information and Other Baseline Characteristics 65](#_Toc84602045)

[15.2.4 Efficacy Analysis 65](#_Toc84602049)

[15.2.5 Safety Analysis 67](#_Toc84602050)

[15.2.6 Adverse Events 67](#_Toc84602051)

[15.2.7 Other Safety Endpoints 67](#_Toc84602052)

[15.2.8 Handling Missing Data 67](#_Toc84602053)

[15.3 Independent Data Monitoring Committee (IDMC) and Interim Analysis 68](#_Toc84602054)

[15.3.1 Independent Data Monitoring Committee 68](#_Toc84602055)

[15.3.2 Interim Analysis 68](#_Toc84602056)

[16 CRITEERIA, METHODS AND REPORTING OF SAFETY ASSESSMENT INCLUDING ADVERSE EVENTS 69](#_Toc84602057)

[16.1 Definition of Adverse Events 69](#_Toc84602058)

[16.2 Definition of Serious Adverse Event, SAE 69](#_Toc84602059)

[16.3 Assessment Criteria of Adverse Events 70](#_Toc84602072)

[16.3.1 Severity of Adverse Events 70](#_Toc84602073)

[16.3.2 Causal Relationship to the investigational product 71](#_Toc84602099)

[16.4 Reporting Adverse Events 72](#_Toc84602130)

[16.5 Follow-up of AEs 73](#_Toc84602141)

[17 INFORMED CONSENT FORM, COMPENSATION AGREEMENT AND TREATMENT FOR DROPOUTS 73](#_Toc84602142)

[17.1 Subject Statement and Consent Form 73](#_Toc84602143)

[17.2 Reimbursement for Patients 73](#_Toc84602144)

[17.3 Treatment Criteria for Subjects After Clinical Trial 73](#_Toc84602145)

[18 MEASURES FOR THE SAFETY PROTECTION OF SUBJECTS 73](#_Toc84602146)

[18.1 Record 73](#_Toc84602147)

[18.2 Tumor Progression during the Trial 73](#_Toc84602148)

[19 OTHER MATTERS FOR SAFE AND SCIENTIFIC TRIAL 73](#_Toc84602149)

[19.1 Institutional Review Board (IRB) 74](#_Toc84602150)

[19.2 Obligations and Delegation of the Principal Investigator 74](#_Toc84602151)

[19.3 Informed Consent 74](#_Toc84602152)

[19.4 Approval of Protocol 74](#_Toc84602154)

[19.5 Revision of Protocol 74](#_Toc84602155)

[19.6 Confidentiality 74](#_Toc84602156)

[19.7 Monitoring and Audit for Protocol Compliance 75](#_Toc84602157)

[19.8 Collection of Data, Recording and Use of Results 75](#_Toc84602158)

[19.8.1 Collection of Data 75](#_Toc84602159)

[19.8.2 Record of the Results 75](#_Toc84602160)

[19.8.3 Submission and Publication of Report 76](#_Toc84602161)

[20 REFERENCES 77](#_Toc84602162)

# TITLE AND PHASE OF STUDY

## Title

A prospective, randomized, open-label, multicenter, parallel design, phase III study to assess the efficacy and safety of GV1001 concurrent with Gemcitabine/Capecitabine versus Gemcitabine/Capecitabine alone in treating locally advanced and metastatic pancreatic cancer patients

## Phase

Phase III

# INSTITUTIONS AND ADDRESS

The list is presented in Appendix 3.

# PPRINCIPAL INVESTIGATORS AND INSTITUTIONS

The list is presented in Appendix 3.

## Sub-investigators and Pharmacists

The list is presented in Appendix 4.

# NAME AND ADDRESS OF SPONSOR

## Name and Address of Sponsor

Sponsor: Samsung Pharm. Ltd.

CEO: Sang-Jae Kim, Ki-Ho Kim

Address: 35, Jeyakgongdan 2-gil, Hyangnam-eup, Hwaseong-si, Gyeonggi-do

# INTRODUCTION

## Locally Advanced and Metastatic Pancreatic Cancer

Pancreatic cancer is known to be one of the digestive system cancers with a high mortality-to-incidence ratio, while its incidence varies from country to country. In South Korea, pancreatic cancer is the 5^th^ most common cancer that causes deaths. Although only 2% of all cancer patients have pancreatic cancer, it accounts for approximately 7% of all cancer deaths^[1],[2]^. Of particular note is pancreatic ductal adenocarcinoma, the 8^th^ most common malignant tumor characterized by poor prognosis with a 5-year survival rate of 8.8% (2008~2012)^[3]^.

The poor prognosis of pancreatic cancer is due to its biological characteristics and difficult surgical resection. Locally advanced or distant metastasis is diagnosed in about 80-90% of patients at the time of admission, and the 5-year survival rate is less than 10%, showing a high recurrence rate of about 70-80% even if it is detected early and radical surgery is performed^[4, 5].^ In particular, multiple liver metastases and recurrence of lymph nodes have been reported to be frequent within 2-3 months after surgery, and liver metastases that are not clinically detected are frequent, making treatment difficult ^[4]^. Although chemotherapy and radiation therapy, which are necessary for patients who cannot be surgically resected, are being developed in terms of improvement of survival rate and symptom improvement, the only way to expect long-term survival rate is to perform radical resection after early detection ^[6, 7]^ .

Locally advanced pancreatic cancer is a pancreatic cancer that is unresectable despite having only local lesions with no metastasis, which, together with metastatic pancreatic cancer, constitutes advanced pancreatic cancer. Approximately 5-25% of all patients with pancreatic cancer are surgically resectable at the time of diagnosis; in those patients, median duration of survival is 13-20 months. However, the majority of patients indicate metastasis at the time of diagnosis with a poor prognosis of around 6 months of survival^[4][8]^. According to the 2012 National Cancer Registry data, the 5-year survival rate of pancreatic cancer by stage was 27.4% for localized stage (cancer does not leave the organ in which it occured), but locally advanced (invasion of surrounding organs, adjacent tissues, or lymph nodes outside of the cancerous organ) or distant metastasis (metastasis to other sites far from the organ where the cancer occurred) was 12.5% ​​and 1.7%, respectively, indicating a drastic decrease in the survival rate depending on the degree of pancreatic cancer progression ^[3]^.

In the treatment of pancreatic cancer, the goal of anticancer therapy is to suppress cancer progression thereby alleviating the patients’ symptoms, improving their quality of life, and ultimately extending their survival^[8]^. For the treatment of pancreatic cancer, like other digestive cancers, 5-fluorouracil (5-FU), an anti-metabolite that inhibits thymidylate synthase, was used as the most basic drug before gemcitabine was released, but the survival rate in 5-FU-based chemotherapy did not show a clear effect on the improvement of clinical symptoms. Since the introduction of gemcitabine, 5-FU has not been used as a first-line treatment and has been used as a second-line treatment for patients who are ineffective in gemcitabine ^[8, 9]^. In the early 1990s, gemcitabine (Gemzar^®^), a pyrimidine anti-metabolite, was developed and reported to contribute to improvement in clinical symptoms, slight improvement in median survival, and improvement in annual survival in comparison with a conventional anticancer agent, 5-fluorouracil (5-FU). Based on these findings, gemcitabine was approved in 1997 by the United Sates Food and Drug Administration as first-line therapy for advanced pancreatic cancer^[6], [7]^. It is the first drug approved in terms of improvement of clinical symptoms, not in terms of anticancer effects, and no anticancer drugs with better results have been developed so far ^[9,10]^.

Continuous attempts have been made to further improve the therapeutic effect of gemcitabine by combining it with other anticancer agents. While co-administration of gemcitabine with 5-FU^[11]^, cisplatin^[12]^, bevacizumab^[13]^, erlotinib^[14]^, and TS-1^[15]^ etc might be feasible, no studies have indicated to date a significantly higher survival with combination therapy than with gemcitabine alone, with the exception of combination with erlotinib. When erlotinib and gemcitabine were administered in combination, which showed a significant increase in survival, the effect of prolonging the survival period was 0.33 months, and there was a limitation in that the incidence of side effects was high ^[14]^. In addition, when the oral 5-FU formulation, capecitabine and gemcitabine, was administered in combination, the survival period was slightly prolonged in the capecitabine group (Gemcitabine/capecitabine combination: 8.4 months, gemcitabine administration: 7.2 months), but there was no statistically significant difference. not seen (p-value=0.234). However, a statistically significant prolongation of survival (gemcitabine/capecitabine combination administration: 10.1 months, gemcitabine administration: 7.4 months, p-value=0.014) was shown when a subgroup analysis of patients with a Karnofsky performance score of 90 or higher was performed ^[16]^. Although co-administration of gemcitabine with other anticancer therapy did not result in significant improvement in survival, it did not lead to severe side effects either when used in patients with good general conditions, which is why gemcitabine in combination with other anticancer agents has been used as an option for first-line therapy in this population.

GV1001 is a peptide consisting of 16 important amino acids in the catalytic component of the telomerase. Recognizing this peptide as an antigen, T cells move toward and kill cancer cells with activated telomerase. As telomerase is proven to be overexpressed more than 90% with advanced pancreatic cancer, GV1001 is expected to be effective in the treatment of advanced pancreatic cancer when co-administered with gemcitabine/capecitabine, the conventional therapy for pancreatic cancer^[17]^.

The present study was designed to evaluate the efficacy and safety of GV1001, an anticancer immunotherapy targeting telomerase, in combination with the conventional therapy gemcitabine/capecitabine in patients with locally advanced and metastatic pancreatic cancer.

## Clinical Trials of GV1001

Since 2000, a total of 17 clinical trials with GV1001 have been completed or are ongoing in patients with locally advanced and metastatic pancreatic cancer, surgically unresectable Stage 3 non-small cell lung cancer (NSCLC), melanoma, liver cancer, and solid tumor, obtaining sufficient safety data of GV1001 from approximately 2,100 patients treated.

Of those, a previous clinical trial conducted in the United Kingdom (UK) in patients with locally advanced and metastatic pancreatic cancer (TeloVac)^[18]^ showed a median survival of 7.89, 6.94, and 8.36 months and time to tumor progression (TTP) of 6.35, 4.54, 6.58 months in the gemcitabine/capecitabine group, the gemcitabine/capecitabine plus GV1001 sequential treatment group, and the gemcitabine/capecitabine plus GV1001 concomitant treatment group, respectively, although there was no statistically significant difference between the gemcitabine/capecitabine group and the GV1001 sequential and/or concomitant treatment groups.

However, in the analysis of 27 cytokines in the serums of patients who participated in the TeloVac study, a subgroup analysis of eotaxin, a biomarker, classified as ‘high eotaxin (>81.02 pg/mL)’ versus ‘low eotaxin (≤81.02 pg/mL)’ in the GV1001 concomitant treatment group showed a median survival of 14.8 months in ‘high eotaxin’ patients versus 7.9 months in ‘low eotaxin’ patients; the difference between groups in median survival was significant ^[19]^. Based on these results, conditional approval was granted for GV1001 by the Ministry of Food and Drug Safety (MFDS) of South Korea on 15 Sep 2014 as a new drug under the brand name of Riavax Inj. for the treatment of pancreatic cancer^[20]^.

The typical trials of GV1001 is presented in Table 5.2.1 below (refer to ‘Investigator’s Brochoure).

Table 5.2.1 Clinical Trials of GV1001

| **Enrolled Subjects** | **GV1001 Dose** | **No. of Subjects (By Group)** | **Concomitant Therapy** | **Adjuvant** | **Immune Response** | **OS (median)** |
| --- | --- | --- | --- | --- | --- | --- |
| **Pancreatic cancer** | | | | | | |
| **CTN 1/2000(Norway)** | | | | | **Phase I/II** |  |
| 49  (48 exposed) | 0.11mg | 11 | None | 30μg Leucomax^®^ | 37.5%(3/8) | 4.0 months |
|  | 0.56mg | 17 | None | 30μg Leucomax^®^ | 75.0%(12/16) | 8.6 months |
|  | 1.87mg | 20 | None | 30μg Leucomax^®^ | 64.3%(9/14) | 5.1 months |
| **CTN 4/2003(Sweden)** | | | | | **Phase I/II** |  |
| 16 | 0.56mg | 7 | Gemcitabine | 150 μg Leukine^®^ | 50.0%(3/6) |  |
|  | 0.56mg | 9 | Gemcitabine | 150 μg Leukine^®^+additional adminstration | 50.0%(3/6) |  |
| **CTN 6/2003(Sweden)** | | | | | **Phase I/II** |  |
| 40(39 exposed) | 0.56mg | 15 | - | Imiquimod (Aldara) | 30.8%  (4/13) |  |
|  | 0.56mg | 10 | Radiation prior to GV1001 | Imiquimod (Aldara) + 75 μg Leukine^®^ | 50.0%  (5/10) |  |
|  | 0.56mg | 14 | Cyclophosphamide prior to GV1001 | Imiquimod(Aldara)+75 μg Leukine^®^ | 57.1%  (8/14) |  |
| **CTN 7/2003(Sweden)** | | | | | **Phase I/II** |  |
| 3 | 0.56mg | 3 | None | 150 μg Leukine^®^ | 100%(3/3) |  |
| **PX115.1.1-302 _ Primovax (Multicentre)** | | | | | **Phase III** |  |
| Planned 520 | 0.56mg | Planned 260 | At progression comb. With Gembitabine | 75 μg Leukine^®^ | Pending | 5.9 months |
|  | None | Planned 260 | Gemcitabine | None | Pending | 7.3 months |
| **PX115.1.1-301_Telovac (Multicentre, UK)** | | | | | **Phase III** |  |
| 1062 | 0.56mg | 350 | Sequential Gemcitabine Capecitabine | 75 μg Leukine^®^ | 38% (12/32) | 6.94  months |
|  | 0.56mg | 354 | Concomitant Gemcitabine Capecitabine | 75 μg Leukine^®^ | 32% (22/68) | 8.36  months |
|  | None | 358 | Gemcitabine Capecitabine | None |  | 7.89  months |
| **PHS IRB# 10-141B(Single centre, USA)** | | | | | **Phase I** |  |
| Planned 9 | 0.56mg | 10 | Tadalafil, Gemcitabine | Sargramostim 30mcg | Pending |  |
| **Liver cancer** | | | | | | |
| **PX115.1.1-201_Heptovax (Multicentre)** | | | | | **Phase II** |  |
| Planned 41 | 0.56mg | 40 | Cyclophosphamide prior to GV1001 | 75 μg Leukine^®^ | Pending | 358 days |
| **Non Small Cell Lung Cancer (NSCLC)** | | | | | | |
| **CTN 3/2000(Norway)** | | | | | **Phase I/II** |  |
| 27 (only 26 exposed) | 0.11mg | 12 | HR2822 (0.07mg) | 30μg Leucomax^®^ | 58.3%  (7/12-towards GV1001) |  |
|  | 0.56mg | 14 | HR2822  (0.07mg) first 10 weeks | 75 μg Leucomax^®^ | 33.3%  (4/12-towards GV1001) |  |
| **CTN 8/2006 (Norway)** | | | | | **Phase II** |  |
| 23 | 0.56mg | 23 | Chemo-radiotherapy prior to GV1001 | 75 μg Leukine^®^ |  |  |
| **Malignant Melanoma** | | | | | | |
| PO3700 (Norway) | | | | | **Phase I/II** |  |
| **27 (26 exposed)** | 0.56mg | 26 | Temozolomide | 75 μg Leucomax^®^ | 78%  (18/23) |  |
| **hTERT-1/01 (Switzerland)** | | | | | **Phase I/II** |  |
| 16 | 0.11mg | 3 | HR2822  (0.07mg) | 30 μg Leucomax^®^ | 0(0/3) |  |
|  | 0.56mg | 7 | HR2822  (0.34mg) | 30 μg Leucomax^®^ | 86%(6/7) |  |
|  | 0.11mg | 6 | HR2822  (0.07mg) | Tuberculin | Unknown |  |
| **Cancer Patients after Operation with Curative Intent** | | | | | | |
| **LTX-315 Study C09-315-02** | | | | | **Phase I** |  |
| 12 | GV1001 0.56mg (0.20mL, 2.8mg/mL) | 12 |  | LTX-315 (0.10mL)  in escalating concentrations, 0.5, 1.0, 1.5, and 2.0mg/mL | 25% (3/12) |  |

## Mechanism of Immune Vaccine GV1001

Telomerase is a human enzyme that is rarely observed in normal cells but is characterized by cancer cell-specific overexpression in the majority of cancers ^[21]^. To allow continuous cell divisions rather than cell aging leading to cell deaths, tumor cells protect telomeres located at the end of the chromosomes so that the cells divide more times than normal cell division count, leading to extended longevity of the tumor cells. GV1001 is a peptide consisting of 16 important amino acids in the catalytic component of the telomerase. Recognizing this peptide as an antigen, T cells move toward and kill cancer cells with activated telomerase.

## Adjuvant GM-CSF

Due to difficulty of effecting immune responses with anticancer immunotherapy alone, it is necessary to administer an adjuvant to increase T-helper type 1 or 2 immune responses^[22]^. Several adjuvants ^[23,24]^ for use with carbohydrate and protein/peptide-based vaccines are being studied. Among them, aluminum hydroxide ^[25]^, the only adjuvant approved as an adjuvant for human vaccines, has been shown to demonstrate the effectiveness of peptide-based vaccines. It was not used as an adjuvant for GV1001 because it did not sufficiently increase the immune response (type 1) required for purpose of peptide based vaccine. In several animal experiments, when GM-CSF and antigen were administered together as a single dose intradermally, it was shown to induce a cellular immune response compared to Freund's adjuvant and aluminum hydroxide ^[26]^.

Granulocyte monocyte-colony stimulating factor (GM-CSF), an adjuvant for anticancer immunotherapy, serves as an adjuvant for immunotherapy to enhance humoral and cellular immune responses. In addition, GM-CSF induces anti-tumor immune responses associated with the induction of T-cell cytokine responses (type 1 and type 2)^[27]^

Based on its effect as a potent adjuvant when co-administered with protein antigens, GM-CSF was selected as an adjuvant for the development of GV1001^[28-30]^. Over the past few years, GM-CSF has frequently been used in clinical trials as an adjuvant for peptide anticancer immunotherapy. Initially, Leucomax® (Molgramostim) was used in the development of GV1001 with GM-CSF, but it is not currently on the market, so in this trial it was changed into Leukine® (Sargramostim). GM-CSF can be used as a monotherapy [31] or as a combination therapy with other adjuvants [32], and 30-250 mcg is usually administered. The dose of GM-CSF investigated in this study is 75 mcg, the same dose as in a previous clinical trial in patients with locally advanced and metastatic pancreatic cancer (TeloVac).

## Rationale for New Response Criteria for Immunotherapy

Growing clinical experience suggests that traditional response criteria will not be sufficient to characterize the activity of targeted therapies and/or biologics in new era. For example, a stable lesion (SD) is determined if the degree of increase or decrease in tumor burden is insufficient to determine disease progression (PD) or partial response (PR), respectively. Stable lesions (SD) during chemotherapy can often be identified temporarily, but are not judged to exhibit actual antitumor activity. On the other hand, in the case of tyrosine kinase inhibitors (e.g., drugs targeting the epidermal growth factor receptor [EGFR] of NSCLC), SD is recognized as a potential surrogate endpoint for improving clinical outcomes ^[33]^. Therefore, the interpretation of the endpoints of the World Health Organization (WHO) and the Response Evaluation Criteria in Solid Tumors Version 1.1 (RECIST 1.1) criteria have been revised in recent years, and the criteria are designed to evaluate the response to cytotoxic drug treatment, so it is not suitable for application to all new drugs. This is because these criteria do not take into account sustained moderate tumor regression or delayed SD upon administration of immunological therapeutics. However, because SD is a mixture of tumor growth and drug effects, the effect of drugs should not be judged prematurely. In some cases, SD is judged as the basis for the expression of drug effect ^[34]^, and SD can also be judged as an index for a meaningful therapeutic effect in immunological therapeutics that increase the anticancer immune response ^[35]^. In addition, additional novel response patterns observed in immunotherapeutic agents raise concerns about the interpretation of activity using WHO or RECIST criteria. It has been shouwn to have occurred that complete response (CR), partial response (PR), or stable lesion (SD) was identified after PD was found by WHO or RECIST in clinical trials of cytokines, cancer vaccines, and monoclonal antibodies (e.g., ipilimumab) ^[36-39]^. For example, in patients with human immunodeficiency virus (HIV)-associated Kaposi's sarcoma being treated with stable antiviral therapy, the anticancer response to recombinant interleukin-12 varied from patient to patient over a wide time interval, with an objective response after apparent PD. Therefore, because PD (in initial radiological evaluation) does not necessarily mean treatment failure, existing response criteria may not be suitable for evaluating the activity of immunotherapeutic agents. Therefore, long-term effects on target diseases must be collected ^[40]^.

In immunotherapy in advanced melanoma, a systematic criterion, the Immune-Related Response Criteria (irRC), has been defined to collect additional patterns of observed responses not described by RECIST or WHO criteria ^[40, 41]^, and this irRC will be used in this clinical trial. In addition, central readers will evaluate disease response on patient scans using both RECIST 1.1 and irRC criteria.

# STUDY OBJECTIVES

## Primary Objective

To compare overall survival (OS) between the two treatment groups (the GV1001+gemcitabine/capecitabine study group versus the gemcitabine/capecitabine control group) in patients with locally advanced and metastatic pancreatic cancer.

.

## Secondary Objective

To compare the following efficacy and safety endpoints between the two treatment groups (the GV1001+gemcitabine/capecitabine study group versus the gemcitabine/capecitabine control group):

1. Time to tumor progression (TTP)
2. Objective response rate (ORR)
3. Clinical benefit response (CBR)
4. Assessment of correlation between eotaxin level and treatment response
5. Quality of life (QoL)
6. Change in CA19-9 over time
7. Safety and tolerability

## Exploratory Objectives

1. Assessment of immune response

- T-cell proliferation

- Delayed-type hypersensitivity (DTH)

# INVESTIGATIONAL PRODUCT

## Identity of Investigational Product

### GV1001

GV1001, a therapeutic telomerase peptide developed as anticancer immunotherapy, consists of a 16-mer peptide corresponding to amino acids 611–626 of human telomerase reverse transcriptase.

As an IP, GV1001 was supplied as white, lyophilized powder in sterile vials. Each vital contained 0.84 mg (0.45 μmole) of the peptide as an active ingredient. GV1001 was prepared by injecting 0.30 mL of saline for IV injection (0.9% w/v) into the vial and gently rotating the vial until the powder was completely dissolved and a clear solution was obtained. The solution contained 2.8 mg/mL tertomotide. The reconstituted substance was to be used within 6 hours after preparation. The IP was to be handled according to guidelines for handling of general sterile medicinal products for injection.

| IP | GV1001 |
| --- | --- |
| Formulation | Lyophilized powder for ID injection |
| Active ingredient/strength | Tertomotide/0.84 mg ± 0.084 mg |
| Excipient | None |
| Vial | Sterile, transparent vial (type I) with a rubber stopper for a single dose of 3.5 mL |
| Manufacturer | Samsung Pharm. Ltd., South Korea. |
| Supplier | Samsung Pharm. Ltd., South Korea. |
| Storage temperature/ duration | - Store in a sealed container at -25 ~ -15°C (frozen) /36 months from the date of manufacture - Store in a sealed container at 2 ~ 8°C (refrigerated) /12 months from the date of manufacture; 12 months after 43 months of storage in a freezer (-25 ~ -15°C) |
| Storage after dilution | Administer the solution as soon as possible after dilution with saline, but within 6 hours after dilution.  Store under refrigeration at 2-8 °C until before dosing. |
| Batch No. (expiry date) | IRA 401(2017.11.24), IRA 701 (2020.01.06) |

### Adjuvant GM-CSH

A growth and differentiation factor for Langerhans cells in the epidermis, GM-CSF is proven to be effective as an adjuvant for anticancer immunotherapy in the induction of immune responses. GM-CSF by itself does not have any activity including anticancer activity.

GM-CSF was supplied as white, lyophilized powder in sterile vials. Each vial contained 250 mcg of sargramostim. GM-CSF was prepared by injecting 0.5 mL of sterile water for injection into the vial and gently rotating the vial until the powder was completely dissolved and a clear solution was obtained. The solution contained 0.5 mg/mL GM-CSF (sargramostim). The reconstituted substance with sterile water for injection was to be used within 6 hours after preparation. The IP was to be handled according to guidelines for handling of general sterile medicinal products for injection.

| Brand name | Leukine^®^ (active ingredient : Sargramostim) |
| --- | --- |
| Formulation | White, lyophilized powder for injection or infusion |
| Active ingredient/strength | Granulocyte-macrophage colony-stimulating factor (GM-CSF), 1.4 x 106 IU/vial (250 mcg) |
| Excipient | Mannitol, sucrose, tromethamine |
| Vial | Sterile vial containing 250 mcg of lyophilized sargramostim |
| Manufacturer | Sanofi-Aventis U.S. LLC |
| Supplier | Samsung Pharm. Ltd., South Korea |
| Storage temperature/ duration | Store under refrigeration at 2-8°C/shelf life was to be specified on the label. |
| Storage after dilution | - Use within 6 hours after dilution with sterile water for solution - Store under refrigeration at 2-8 °C until before dosing |
| Batch No. (expiry date) | B21187 (2016.11.30), E6010E (2020.02.29), E6041E (2020.12.31), E7017E (2021.05.31) |

### Gemcitabine

Gemcitabine is a nucleoside analogue anticancer agent that inhibits deoxyribonucleic acid (DNA) synthesis.

| Active ingredient | Gemcitabine HCl |
| --- | --- |
| Formulation | White to off-white lyophilized cake or powder which, when dissolved, appears as a clear, colorless to off-white solution |
| Active ingredient/strength | Gemcitabine HCl 1,140mg (1,000 mg as gemcitabine base), 228 mg (200 mg as gemcitabine base) |
| Excipient | Mannitol, sodium acetate, hydrochloric acid, sodium hydroxide |
| Vial | Sterile vial containing 1,000 mg or 200 mg of lyophilized gemcitabine |
| Supplier | Samsung Pharm. Ltd., South Korea |
| Storage temperature/ duration | Store at ambient temperature of 15-30°C/3 years |
| Storage after dilution | Use within 24 hours after dilution with saline. |
| Batch No. (expiry date) | S001 (2018.02.09), GERAVS002 (2018.07.23), AGS501 (2018.11.10), AGS701 (2020.04.23), AGS801 (2021.08.15) |

| Active ingredient | Gemcitabine HCl |
| --- | --- |
| Formulation | White to off-white lyophilized cake or powder which, when dissolved, appears as a clear, colorless to off-white solution |
| Active ingredient/strength | Gemcitabine HCl 1,140mg (1,000 mg as gemcitabine base), 228 mg (200 mg as gemcitabine base) |
| Excipient | Mannitol, sodium acetate, hydrochloric acid, sodium hydroxide |
| Vial | Sterile vial containing 1,000 mg or 200 mg of lyophilized gemcitabine |
| Supplier | Samsung Pharm. Ltd., South Korea |
| Storage temperature/ duration | Store at ambient temperature of 15-30°C/3 years |
| Storage after dilution | Use within 24 hours after dilution with saline. |
| Batch No. (expiry date) | S001 (2018.02.09), GERAVS002 (2018.07.23), AGS501 (2018.11.10), AGS701 (2020.04.23), AGS801 (2021.08.15) |

The drug product was to be diluted with saline within 24 hours prior to dosing. The IP was to be handled according to guidelines for handling of general sterile medicinal products for injection.

### Capecitabine

Capecitabine, a fluoropyrimidine carbamate anticancer agent, is a prodrug of 5´-deoxy-5-fluorouridine which itself is a prodrug of 5-FU. Upon oral administration, capecitabine is converted into an active form of 5-FU at the site of tumor to exert anticancer activity.

| Active ingredient | Capecitabine |
| --- | --- |
| Formulation | Light pink-coloured, oval, film-coated tablet |
| Active ingredient/strength | Capecitabine 150 mg, 500 mg |
| Supplier | Samsung Pharm. Ltd., South Korea |
| Storage temperature/ duration | Store at ambient temperature of 1-30°C/3 years |
| Batch No. (expiry date) | - 150 mg Tab.: 3001 (2016.08.08), XELCTT001 (2019.01.03), XELCTX001 (2021.03.11) - 500 mg Tab.: 3001 (2016.08.08), 3002 (2016.08.08), 3003 (2016.08.08), XELTTS001 (2018.07.26), XELTTS002 (2018.07.26), XELTTS003 (2018.07.26), XELTTX001 (2021.03.11) |

## Investigational Product Labeling

According to a partial provisory clause other than the subparagraphs of Paragraph 1, Article 56 of Pharmaceutical Affairs Act, the container or package of the IP was to be labeled with the following information:

- Statement of “for investigational use only”
- Product code or generic name of active ingredient
- Lot number and expiry date (shelf-life) or re-test date
- Storage conditions
- Name and address of the Investigational New Drug (IND) holder
- Statement of “not permitted for any purpose other than investigational use”

## Supply, Storage, and Management of the Investigational Product

GV1001 was manufactured and supplied by Samsung Pharm. Ltd. GM-CSF was imported from Sanofi-Aventis and supplied to each of the study sites by Samsung Pharm. Ltd. Gemcitabine and capecitabine were purchased and supplied to each of the study sites by Samsung Pharm. Ltd.

By signing applicable documents, the PI confirmed the receipt and quantity of IPs supplied by the sponsor. The IPs were dispensed as prescribed by the investigators participating in this study and documented in investigational product accountability logs. Under no circumstances the IPs were used for any purpose other than the protocol-defined use.

# INDICATION

Locally advanced or metastatic pancreatic cancer

# INCLUSION/EXCLUSION CRITERIA, PLANNED NUMVER OF SUBJECTS AND THE RATIONALE

## Inclusion Criteria

Eligible subjects met **all** of the following criteria:

1. Age of ≥ 19 years
2. Histopathologically or cytologically proven pancreatic ductal adenocarcinoma or undifferentiated carcinoma of the pancreas
3. Locally advanced or metastatic disease precluding curative surgical resection or patients who have relapsed following previously resected pancreatic cancer; the patient must have measurable lesions.
4. Contrast enhanced CT scans on the thorax, abdomen, and pelvis within 28 days (and up to a maximum of 32 days) prior to commencing IP treatment (patients who are unable to receive contrast due to allergies etc. may still be eligible for this trial if it is possible to perform tumor assessment based on non-contrast enhanced CT scans.)

* Those who cannot undergo CT scanning may be tested by MRI.

** Images (e.g., positron emission tomography [PET]-CT, etc.) obtained within the relevant period may be used if available.

1. Measurable lesions on the screening CT scan (or MRI) according to RECIST guideline (see Protocol Appendix 4. RECIST guideline v1.1)
2. Eastern Cooperative Oncology Group (ECOG) performance status 0, 1, or 2 (see Protocol Appendix 2. ECOG Performance Status)
3. Adequate organ function as determined by pre-enrollment clinical laboratory tests:

- Platelets ≥ 100 x 10^9^/L
- WBC ≥ 3 x 10^9^/L
- ANC ≥ 1.5 x 10^9^/L
- Serum total bilirubin ≤ 2.0 mg/dL
- CCr (Cockcroft&Gault) > 50 mL/min

1. Life expectancy ≥ 90 days
2. The subject or his/her legally acceptable representative must provide written informed consent prior to study initiation, and the subject must be capable of complying with study requirements.

## Exclusion Criteria

Patients who met any of the following criteria could not be enrolled in this study:

1. Brain metastasis or meningeal carcinomatosis
2. Clinically significant serious disease or organ system disease not currently controlled on present therapy
3. Previous chemotherapy for locally advanced and metastatic disease. (However, previous adjuvant chemotherapy for resected pancreatic cancer are permitted providing chemotherapy was completed more than 12 months previously prior to enrollment.)
4. Radiotherapy within the last 8 weeks prior to enrollment
5. Concurrent malignancies or invasive cancer diagnosed within 5 years prior to enrollment (except for treated Basal Cell Carcinoma of the skin, in situ carcinoma of the uterine cervix or resected pancreatic cancer)
6. Medication which might affect immunocompetence e.g. chronic treatment with long term steroids or other immunosuppressant. Patients are eligible if they have been receiving short term steroids for palliation of cancer related symptoms.
7. Having participated in another clinical trial to be treated with another IP within 8 weeks prior to enrollment.
8. Pregnancy or breast feeding
9. Unstable angina pectoris
10. Known malabsorption syndromes
11. Patients with a known hypersensitivity reactions to the active ingredient and any of the excipients of the IP or patients with a dihydropyrimidine dehydrogenase deficiency
12. All men or women of reproductive potential, unless using at least two contraceptive precautions, one of which must be a condom
13. Patients considered by the investigator to be ineligible for this study

## Determination of Sample Size

Rationale for sample size calculation is as follows.

1. **Hypothesis**

Null hypothesis: (Median survival time in the study group will be the same or shorter than that in the control group.)


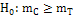

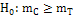


Alternative hypothesis: (Median survival time in the study group will be longer than that in the control group.)


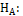

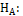

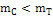

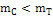


- : Median survival time in the control group


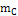

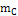


- : Median survival time in the study group


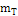

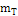


1. **Considerations for sample size calculation**

Literature on overall survival with gemcitabine+capecitabine is summarized below. The weighted average survival time of the 4 reports was approximately 7.9 months, which was assumed as median survival time for the control group.

| **Author** | **The number of subjects** | **Median overall survival time (months)** |
| --- | --- | --- |
| Hermann (2006)^[22]^ | 160 | 8.4 |
| Cunningham (2009)^[25]^ | 267 | 7.1 |
| Choi (2012)^[26]^ | 50 | 10 |
| Middleton (2014)^[19]^ | 358 | 7.9 |

In terms of median survival time for the study group, a subgroup analysis in the TeloVac phase 3 clinical trial published by Neoptolemose et al. (2014) ^[19]^, reported a median survival time of 14.8 months in subjects with high eotaxin level in the Gem/Cap+GV1001 combination therapy group, which was assumed as the effect of the study group in the present study.

Using the above estimates, sample size was calculated as follows.

1. Significance level: one-sided 0.025
2. Power: 0.8
3. Randomization ratio 1:1
4. Median survival time: control group (7.9 months), study group (14.8 months)
5. Interim analysis: once
6. Group sequential logrank test based on O’Brien-Fleming type alpha spending function
7. Criteria for premature termination
8. Termination due to significance
9. Termination due to futility: non-binding futility boundary
10. Total duration of study: 54 months (duration of enrollment: 42 months, duration of follow-up: 12 months)
11. Uniform accrual rate
12. Software
13. SAS^®^ 9.3
14. Seqdesign procedure
15. **Results of sample size calculation**

Assuming 7.9 months and 14.8 months of median survival time for the control group and the study group, respectively, with 80% power at 2.5% one-sided significance level (hazard ratio 0.53), it was required to enroll 118 subjects to have 85 events (deaths). With 20% of drop-outs and exclusions from analyses taken into account, the final sample size was set to be 148 subjects (74 subjects each in the study group and the control group).

# DURATION OF STUDY

This study will be completed within 54 months from IRB approval. Enrollment period is 42 months and 12 months of follow-up period will be conducted.

# INVESTIGATIONAL PLAN

## Overall Study Design and Plan

This study is designed as a randomized, open-label, multicenter, parallel-design Phase 3 clinical trial to evaluate the efficacy and safety of GV1001 in combination with gemcitabine/capecitabine versus gemcitabine/capecitabine in patients with locally advanced and metastatic pancreatic cancer.

Subjects eligible for study participation are classified into 2 groups, high eotaxin (>81.02 pg/mL) versus low eotaxin (≤81.02 pg/mL), based on the eotaxin test. Those in the high eotaxin group are randomized to either the study group (GV1001 plus gemcitabine/capecitabine) or the control group (gemcitabine/capecitabine) in a 2:1 ratio while those in the low eotaxin group are allocated to the control group in the same ratio as the control group of the high eotaxin group. In the high eotaxin group, subjects are stratified by progression of pancreatic cancer (locally advanced, metastatic) for randomization. Those in the low eotaxin group are not randomized; they are stratified by progression of pancreatic cancer (locally advanced, metastatic) and enrolled in the control group in the same ratio as the control group of the high eotaxin group.

Eligibility for study participation is assessed within 21 days prior to IP dosing.

Subjects who are determined eligible and enrolled in the study are treated with the IP and assessed for efficacy and safety as planned. All subjects continued to be treated with the IP until progressive disease (PD) is determined by computed tomography (CT) scan (or magnetic resonance imaging [MRI]) and are followed up until death or the end of study (EOS).

To determine PD of pancreatic cancer, CT scanning (or MRI) is performed every 12 weeks from Week 8, and tumor response is assessed according to Response Evaluation Criteria In Solid Tumors (RECIST) and Immune-Related Response Criteria (irRC). The safety and tolerability of the IP are assessed based on vital signs, clinical laboratory tests, adverse events (AEs), etc.

All subjects who completed IP dosing or who are prematurely withdrawn from the study (except for those who withdrew their informed consent) are followed up for survival every 12 weeks until death or EOS.

EOS is defined as any of the following 3 time points: 1) the last subject completed 52 weeks of treatment and/or follow-up, 2) the last on-going patient had been subject to treatment and/or follow-up for at least 52 weeks, or 3) all subjects are discontinued. Therefore, the last subject who is applicable to 1) or 2) undergoes CT scanning (or MRI) for tumor assessment at Week 52.

**1) Study group (GV1001 plus gemcitabine/capecitabine)**

For gemcitabine/capecitabine therapy, one cycle is defined as follows:

- Gemcitabine: once weekly intravenous (IV) doses for 3 consecutive weeks (Days 1, 8, and 15) followed by 1 week off treatment
- Capecitabine: twice daily (morning/evening) oral doses for 21 days followed by 7 days off treatment

Gemcitabine/capecitabine therapy is repeated every 4 weeks until therapy is discontinued due to the subject’s request, occurrence of intolerable toxicity, or PD (see 11.3 ‘Dose, method, and duration of administration).

GV1001 is intradermally (ID) administered on Days 1, 3, and 5 of Week 1 followed by an ID dose at Weeks 2, 3, 4, and 6. Afterwards, GV1001 therapy is repeated every 4 weeks until therapy is discontinued due to the subject’s request, occurrence of intolerable toxicity, or PD. Between 10 and 15 minutes after an ID dose of the adjuvant GM-CSF in the lower abdomen, an ID dose of GV1001 is given at the same site of administration.

Dosing schedules of GV1001 and the adjuvant GM-CSF are not affected by dosing sequences and time intervals with gemcitabine/capecitabine therapy.

All subjects treated with GV1001 are to remain in hospital for at least 1 hour for AE monitoring, and any AEs are to be reported to the PI or the sub-investigator.

CT scanning (or MRI) for efficacy assessment is performed at the screening and Week 8 visits and every 12 weeks thereafter until the subject’s withdrawal. IP treatment is continued until the subject is withdrawn from the study. Upon withdrawal, conventional therapy can be initiated at the discretion of the investigator, and afterwards, the subject is to be followed up every 12 weeks until death or EOS.

As a target lesion might appear to be bigger in the study group due to temporary tumoral inflammation as a result of immune responses activated by GV1001/GM-CSF, additional treatment for 12 weeks is to be allowed at the discretion of the investigator for those whose Week 8 CT scan indicated PD. After the additional treatment, another CT scan is to be obtained to confirm PD.


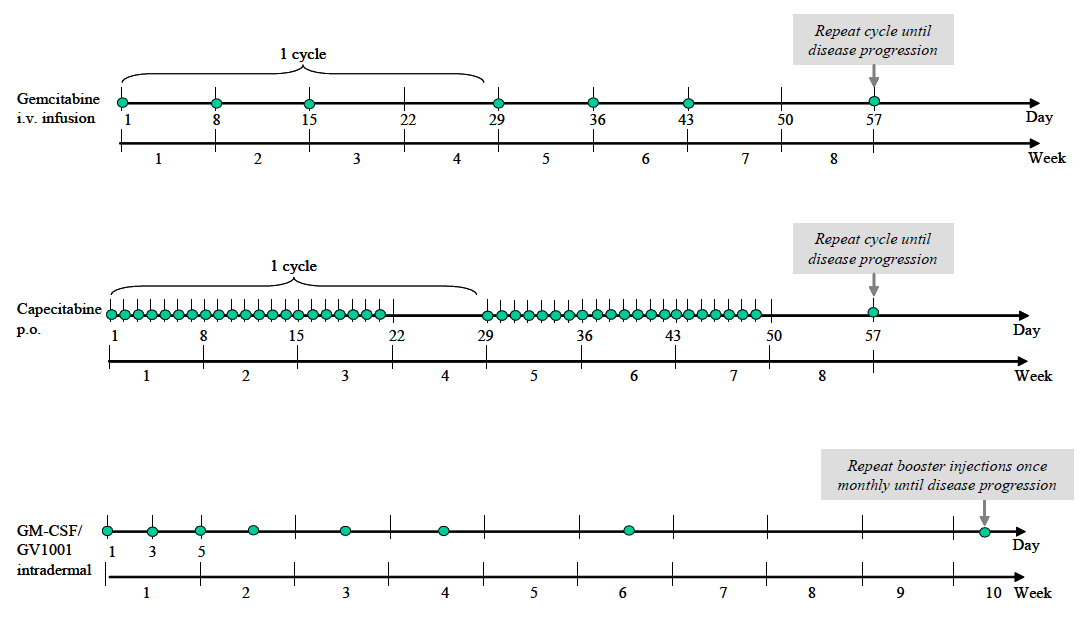


**2) Control group (Gemcitabine/capecitabine)**

For gemcitabine/capecitabine therapy, one cycle was defined as follows:

- Gemcitabine: once weekly IV doses for 3 consecutive weeks (Days 1, 8, and 15) followed by 1 week off treatment
- Capecitabine: twice daily (morning/evening) oral doses for 21 days followed by 7 days off treatment


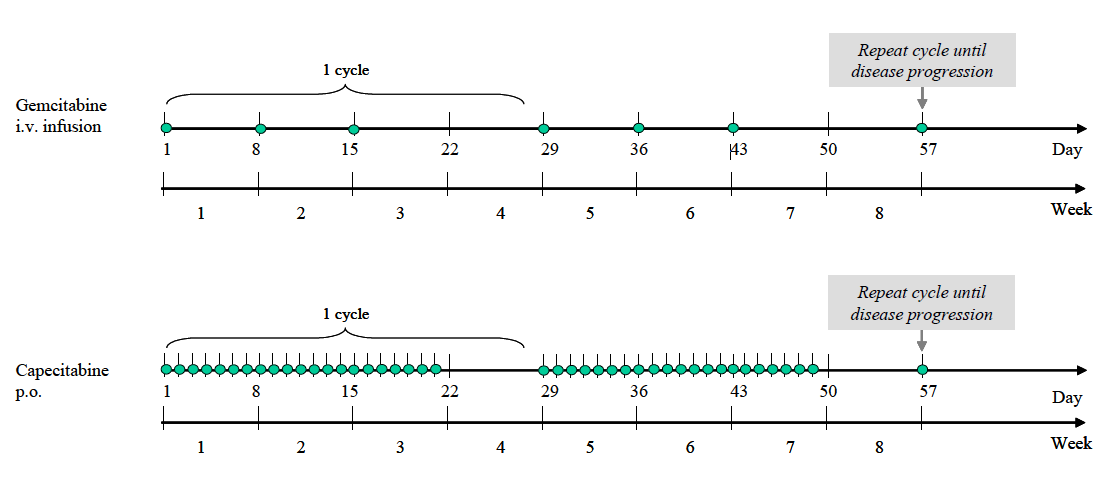


Gemcitabine/capecitabine therapy is repeated every 4 weeks until therapy is discontinued due to the subject’s request, occurrence of intolerable toxicity, or PD (see 11.3 ‘Dose, method, and duration of administration).

CT scanning (or MRI) for efficacy assessment is performed at the screening and Week 8 visits and every 12 weeks thereafter until the subject’s withdrawal. IP treatment is continued until the subject is withdrawn from the study. Upon withdrawal, conventional therapy can be initiated at the discretion of the investigator, and afterwards, the subject is to be followed up every 12 weeks until death or EOS.

SD or responding to treatment

Progressive disease

Progressive disease

SD or responding to treatment

Progressive disease

SD or responding to treatment

SD or responding to treatment

Progressive disease

*See below

* As a target lesion might temporarily appear to be bigger in the study group, additional treatment for 12 weeks was to be allowed at the discretion of the investigator.

Study group

(Gem/Cap + GV1001/GM-CSF)

Control group (Gem/Cap)

*[High:low eotaxin ratio=1:1]*

Week 8 Tumor assessment

Week 8 Tumor assessment

Gem/Cap +

GV1001/GM-CSF

Week 20 Tumor assessment

Additional 12 weeks of treatment

Tumor assessment

(Every 12 weeks)

Follow-up

**Screening (eotaxin testing)**

High eotaxin group

(>81.02 pg/mL)

Low eotaxin group

(≤81.02 pg/mL)

**Randomization (2:1)**

Gem/Cap

Week 20 Tumor assessment

Additional 12 weeks of treatment

Tumor assessment

(Every 12 weeks)

Follow-up

**Figure 11.1.1 Study flow**

## Treatment Breaks

### Simple Treatment Breaks

All subjects are allowed to take a break from treatment for a short period. This elective break can be for personal reasons (e.g., vacations) or simply as a respite from treatment. Those who had long-term anticancer chemotherapy and met the following criteria can have a treatment break of one full cycle of gemcitabine/capecitabine during the study period, regardless of withdrawal criteria. In this case, the treatment break is not included in the duration of missed treatment specified in No. 7 of 14.2 ‘WITHDRAWL CRITERIA’. Only those who met both of the below criteria can have an elective treatment break:

1. Continued treatment with gemcitabine/capecitabine on this study for at least 9 consecutive months
2. The treatment break must be seen as clinically appropriate by the Investigator.

Subjects in the study group continued to be treated with GV1001 every 4 weeks even when they had a break from gemcitabine/capecitabine.

* Upon the request of the subject, a treatment break is implemented after the investigator (or his/her designee) discussed with the responsible clinical research associate (CRA) and the sponsor and confirmed that both of the above criteria are met before the treatment break is allowed.

### Requiring Other Treatment

This was applicable to cases where it was difficult to administer GV1001/GM-CSF or anticancer agents for reasons unrelated to pancreatic cancer (e.g., stent, occlusion) (GV1001 was to be continued if possible even when it was difficult to administer gemcitabine/capecitabine). In this case, one week of delay in IP dosing was allowed. If treatment was stopped prior to the start of a treatment cycle, the start of the cycle was delayed for one week. If treatment was stopped after the start of a cycle, treatment interruption was to be implemented as follows.

- **Control group:**

|  | **1 cycle (=4 weeks)** | | | | **Remark** |
| --- | --- | --- | --- | --- | --- |
|  | **1^st^ Week** | **2^nd^ Week** | **3^rd^ Week** | **4^th^ Week** |  |
| 1. | Treated | Gem/Cap interrupted | Gem/Cap interrupted | Off treatment | Resume treatment at the next scheduled cycle. |
| 2. | Treated | Gem and/or Cap interrupted | Treatment resumed | Off treatment | Resume treatment at the 3^rd^ Week. |

*Gem/Cap: gemcitabine/capecitabine

- **Study group**: The time points of treatment re-start following drug holidays from gemcitabine and capecitabine were the same as in the control group. For treatment interruption with GV1001/GM-CSF, the following table was applied.

| 1. | Start of treatment ~ 4th week | Resume treatment at the next scheduled dosing time point. |
| --- | --- | --- |
| 2. | 5th week ~ | Resume treatment 1 week after the treatment interruption; in the event of treatment failure, resume treatment at the next scheduled dosing time point. |

## Dose, Method and Duration of Administration

### GV1001 and the Adjuvant GM-CSF

#### Administration Preparation, Dose and Method

GV1001 dose is 0.56 mg. It is diluted with 0.30 mL of saline for injection (0.9% w/v) supplied in vials (diluted to 2.8 mg/mL), and 0.2 mL of the diluted solution is administered as an ID injection to the lower abdomen. Once diluted, GV1001 is to be administered immediately but within 6 hours after dilution. Between 10 and 15 minutes prior to GV1001 dosing, the adjuvant GM-CSF (Leukine^®^) is administered as an ID injection (see figure 12.2.1).

GM-CSF dose is 75 mcg. It is diluted with 0.5 mL of sterile water for injection supplied in vials (diluted to 0.5 mg/mL), and 0.15 mL of the diluted solution is administered as an ID injection to the lower abdomen. It is administered immediately after dilution once, and it is administered within a maximum of 6 hours after dilution (see Figure 12.2.1). GM-CSF is used only once and is not re-used.

Dosing schedules of GV1001 and the adjuvant GM-CSF are not affected by dosing sequences and time intervals with gemcitabine/capecitabine therapy.

**Action for Overdose**

GV1001: No specific treatment is known for overdose. Treatment to symptom or supportive care should be given.

GM-CSF: Stop dosing GM-CSF, and monitor the increase of leucocyte number and respiratory symptom..

#### Duration of Administration

One ID dose of GV1001 and GM-CSF is given on Days 1, 3, and 5 of Week 1 followed by one ID dose at Weeks 2, 3, 4, and 6. Afterwards, therapy is repeated every 4 weeks until it is discontinued due to the subject’s request, occurrence of intolerable toxicity, or progressive disease (PD). One ID dose of GV1001 and GM-CSF is given on Days 1, 3, and 5 of Week 1 followed by one ID dose at Weeks 2, 3, 4, and 6. Afterwards, therapy is repeated every 4 weeks until it is discontinued due to the subject’s request, occurrence of intolerable toxicity, or progressive disease (PD).

#### Gemcitabine

#### Preparation, Dose and Method of Administration

Gemcitabine is prepared within 24 hours before dosing, and 1,000 mg/m^2^ was intravenously (IV) administered over ≥ 30 minutes.

If toxicity is observed, dose adjustment is permitted as below according to the label. Dose is adjusted at the discretion of the investigator depending on the subject’s condition.

**Dose adjustment for hematologic toxicity (see the label of gemcitabine)**

Based on absolute neutrophil count (ANC) and platelet count measured prior to gemcitabine dosing, dose adjustment is permitted as below.

**Table 11.3.1 Criteria for dose reduction by absolute neutrophil count and platelet count**

| **ANC (x 10^6^/L)** | **Platelet count (x 10^6^/L)** | **Percentage of total dose** |
| --- | --- | --- |
| ≥ 1,000 AND | ≥ 100,000 | 100% |
| 500 ～ 999 OR | 50,000 ～ 99,999 | 75% |
| < 500 OR | < 50,000 | One week off (omit)* |

* One week off (omit) - test was to be repeated. If ANC or platelet count did not recover over 2 consecutive cycles (8 consecutive weeks), therapy was to be permanently discontinued.

| **Item** | **Criteria** | **Dose adjustment** | |
| --- | --- | --- | --- |
| Hematologic toxicity | ANC and platelet | See Table 9.4.1. | |
|  | Febrile neutropenia  (Fever with ANC < 500 x10^6^/L) | The administration should be temporarily interrupted until recovery, and 75% should be administered during all cycles thereafter. | |
|  | Others except for the above | See criteria for non-hematologic toxicity. | |
| Non-hematologic toxicity | No criteria for dose adjustment. | There are no criteria for dose adjustment. The administration may be temporarily delayed until recovery from toxicity (recovery to National Cancer Institute Common Toxicity Criteria [NCI CTCAE] grade 0-1) at the discretion of the investigator. In this case, upon recovery, it is necessary to administer gemcitabine at 75% of the starting dose. | |
| * In the event of reoccurrence of the same toxicity despite dose reduction to 75% of the starting dose at the next scheduled visit due to any toxicity other than the above criteria based on ANC and platelet count, therapy was temporarily interrupted until recovery of the toxicity to Grade 0 or 1, and upon recovery, therapy was resumed at 50% of the starting dose. In the event of reoccurrence of the same toxicity despite dose reduction to 50% of the starting dose, the subject was withdrawn from the study. | | | |
| **Item** | **Criteria** | **Dose adjustment** |  |
| Hematologic toxicity | ANC and platelet | See Table 9.4.1. |  |
|  | Febrile neutropenia  (Fever with ANC < 500 x10^6^/L) | The administration should be temporarily interrupted until recovery, and 75% should be administered during all cycles thereafter. |  |
|  | Others except for the above | See criteria for non-hematologic toxicity. |  |
| Non-hematologic toxicity | No criteria for dose adjustment. | There are no criteria for dose adjustment. The administration may be temporarily delayed until recovery from toxicity (recovery to National Cancer Institute Common Toxicity Criteria [NCI CTCAE] grade 0-1) at the discretion of the investigator. In this case, upon recovery, it is necessary to administer gemcitabine at 75% of the starting dose. |  |
| * In the event of reoccurrence of the same toxicity despite dose reduction to 75% of the starting dose at the next scheduled visit due to any toxicity other than the above criteria based on ANC and platelet count, therapy was temporarily interrupted until recovery of the toxicity to Grade 0 or 1, and upon recovery, therapy was resumed at 50% of the starting dose. In the event of reoccurrence of the same toxicity despite dose reduction to 50% of the starting dose, the subject was withdrawn from the study. | | |  |

**Action for Overdose (see the label of Gemcitabine)**

There is no known antidote for gemcitabine. If an overdose is suspected, careful observation, blood count tests, and supportive care should be performed if necessary.

#### Duration of Administartion

One cycle of gemcitabine is comprised of once weekly IV doses for 3 consecutive weeks (Days 1, 8, and 15) followed by one week off treatment. Therapy is repeated until the subject’s request, occurrence of intolerable toxicity, or PD.

### Capecitabine

#### Preparation, Dose, and Method of Administration

Capecitabine 830 mg/m^2^ is orally administered twice daily (morning, evening) (1,660 mg/m^2^ per day). Capecitabine is administered with water within 30 minutes after a meal.

If toxicity is observed, dose adjustment is permitted as below according to the label. Dose is adjusted at the discretion of the investigator depending on the subject’s condition.

**Dose adjustment for toxicity (see the label of capecitabine)**

Subjects are carefully monitored for toxicity. Most common AEs with capecitabine were hand-foot syndrome and gastrointestinal disorders such as diarrhea, nausea, vomiting, and gastritis. NCI CTCAE grade 3 and 4 anemia and neutropenia were reported in approximately 2% of subjects. All subjects collected data of medications used for the treatment of diarrhea, gastritis, hand-foot syndrome, etc. Antinauseants (if possible, non-steroidal) could be administered according to site-specific standard of care. For non-hematologic toxicity, dose adjustment is permitted as below at the discretion of the investigator.

Table 9.4.2 Criteria for dose reduction for hematologic toxicity

| **Item** | **Criteria** | **Dose adjustment** |
| --- | --- | --- |
| Hematologic toxicity | Febrile neutropenia  *Or*,  Neutrophil count <1,000 x 10^6^/L  *Or*,  Platelet count <75,000x10^6^/L | The administration should be temporarily interrupted until recovery to NCI CTCAE Grade 0 or 1 and should be re-initiated at 75% of the starting dose.  * In the event of reoccurrence of the same toxicity despite dose reduction to 75% of the starting dose, the administration was temporarily interrupted until recovery of the toxicity to NCI CTCAE Grade 0 or 1, and upon recovery, the administration could be resumed at 50% of the starting dose. In the event of reoccurrence of the same toxicity despite dose reduction to 50% of the starting dose, the subject was withdrawn from the study. If the investigator determined that continued treatment was in the best interest of the subject, the subject could continue to be on study at the lowest dose (50% of the starting dose) upon recovery to NCI CTCAE Grade 0 or 1. |
|  | Others except for the above | See criteria for non-hematologic toxicity. |
| Non-hematologic toxicity | \| **Frequency of NCI CTCAE event** \| **Grade 2** \| **Grade 3** \| **Grade 4** \| \| --- \| --- \| --- \| --- \| \| 1st occurrence of toxicity \| Therapy should be temporarily interrupted until recovery to Grade 0-1 and then resumed at 100% of the starting dose. \| Therapy should be temporarily interrupted until recovery to Grade 0-1 and then resumed at 75% of the starting dose. \| Therapy should be permanently discontinued. If the investigator determines that continued treatment is in the best interest of the subject, therapy should be temporarily interrupted until recovery to Grade 0-1 and then resumed at 50% of the starting dose. \| \| 2nd occurrence of the same toxicity \| Therapy should be temporarily interrupted until recovery to Grade 0-1 and then resumed at 75% of the starting dose. \| Therapy should be temporarily interrupted until recovery to Grade 0-1 and then resumed at 50% of the starting dose. \| Therapy should be permanently discontinued. \| \| 3rd occurrence of the same toxicity \| Therapy should be temporarily interrupted until recovery to Grade 0-1 and then resumed at 50% of the starting dose. \| Therapy should be permanently discontinued. \| - \| \| 4th occurrence of the same toxicity \| Therapy should be permanently discontinued. \| - \| - \|  1. Cardiotoxicity, although uncommon, had been associated with capecitabine therapy. These toxicities include angina, myocardial infarction, cardiac failure, cardiac arrest, sudden death and ECG changes. In any patient developing classical symptoms of angina during capecitabine therapy should be given to discontinuation of capecitabine administration and also referral to a cardiologist. These adverse events may be more common in patients with a prior history of coronary artery disease and therefore caution had to be exercised in patients with a prior history of coronary heart disease, arrhythmia and angina. 2. Renal Impairment: During the study, creatinine clearance should be calculated using the Cockroft and Gault formula. For patients with calculated creatinine clearance reduced down to between 30-50ml/min during treatment, capecitabine was administered at 75% of the starting dose. According to the label, capecitabine is contraindicated for use in individuals with severe renal impairment. Accordingly, subjects with CCr 30 mL/min were withdrawn from the study. 3. Hepatic impairment. In the absence of safety and efficacy data in patients with hepatic impairment, capecitabine use should be carefully monitored in patients with mild to moderate liver dysfunction, regardless of the presence or absence of liver metastasis. Administration of capecitabine was temporarily interrupted if treatment-related elevations in bilirubin of>3.0 x ULN or treatment-related elevations in hepatic aminotransferases (ALT, AST) of >2.5 x ULN occur (>5 x ULN for patients with liver metastasis) and re-administration was started when recovered. If the elevations were unrelated to capecitabine (e.g., already elevated at the time of enrollment), the administration could be initiated as earlier, and dose adjustment was allowed at the discretion of the investigator. 4. In the event of Grade ≥4 toxicity that was unassessable by NCI-CTCAE, therapy was permanently discontinued. | |

**Action for Overdose**

Clinical symptoms for overdose include nausea, vomiting, mucositis, gastrointestinal irritation and bleeding, and myelosuppression. If an overdose is suspected, standard treatment with close observation is performed according to the symptoms.

#### Duration of Administration

One cycle of capecitabine is comprised of twice daily (morning/evening) oral doses for 21 days followed by 7 days off treatment. Therapy is repeated until the subject’s request, occurrence of intolerable toxicity, or PD.

## Selection of Doses in the Study

In general, doses for cytotoxic anticancer agents are determined by increasing doses until before dose limiting toxicities are observed. This method is not appropriate for anticancer immunotherapy as this type of therapy is generally associated with fewer intrinsic toxicities. Given that a dose-response curve after a GV1001 dose indicated an increase in immune response in the early phase of treatment followed by a long plateau eventually leading to a decrease in immune response, GV1001 dose was not selected based on the maximum tolerated dose.

The most important element taken into consideration in determining an appropriate dose was immune response rate. In a GV1001 clinical trial in patients with pancreatic cancer ^[24]^, immune response with GV1001 was 38% at a low dose (0.11 mg), 64% at a high dose (1.87 mg), and 75% at a moderate dose (0.56 mg), with the majority of AEs reported across all dose groups mild in intensity. Based on these data, GV1001 0.56 mg was selected for this study as this dose was associated with the highest immune response rate, higher survival rate, and favorable safety.

## Rational for the Duration of IP Treatment

Immune responses were usually induced between 3 and 6 weeks after the initiation of anticancer immunotherapy. In general, induction of immune responses is limited by short life expectancy and rapidly declining immune function in patients with advanced pancreatic cancer. To induce immune responses as rapidly and effectively as possible, multiple repeat treatment with anticancer immunotherapy is required. The treatment regimen of GV1001 with multiple repeat doses over the first 6 weeks of treatment was established based on a similar regimen used for another peptide anticancer immunotherapy that effectively induced immune responses in patients with advanced pancreatic cancer^[31]^.

## Concomitant Treatment

During the treatment period, concomitant therapy for any disease other than pancreatic cancer is allowed. For drugs allowed by the selection criteria, dose is not to be changed during the treatment period.

Outside of the treatment period, other treatment for pancreatic cancer is allowed at the discretion of the investigator. If surgery is inevitably needed, GV1001 treatment is temporarily interrupted until the outcome of the surgery is known.

### Precautions for Concomitant Use and Prohibited Drugs/Treatment

**Drugs/Treatment for Concomitant Use with Precautions**

- Anticoagulants (e.g., warfarin): requires regular monitoring for blood coagulation factors (prothrombin time or international normalized ratio).
- Phenytoin: requires regular monitoring for serum phenytoin concentration.
- Metronidazole (caution should be exercised for potential interactions with capecitabine.)

**Prohibited Drugs/Treatment**

- Drugs and treatment that may affect the efficacy of the IP such as anticancer treatment and radiotherapy (immunosuppressants, other IPs, other anticancer chemotherapy/radiotherapy)
- Dipyridamole, allopurinol
- Continuous systemic corticosteroids (short-term treatment with low-dose systemic corticosteroids may be allowed at the discretion of the investigator.)

## Method of Assigning Subjects to Treatment Groups

Among individuals who met all of the inclusion/exclusion criteria and provided informed consent to participation in this study, those who are determined eligible are stratified for randomization via Interactive Web Response System (IWRS).

At the screening visit, the subjects are given a screening number after they signed the ICF. Those who had a high eotaxin level at the time of the screening visit (within 7 days prior to IP dosing) are randomized while those who had a low eotaxin level are not randomized but are all included in the control group. Only those who are randomized are given a randomization number in the order of randomization.

Screening/randomization numbers assigned to each subject are used through to the end of the study and documented in eCRFs. Progression of pancreatic cancer (locally advanced, metastatic) is considered as a stratification factor for subject enrollment.

A screening/randomization number is comprised as follows:

- Site code assigned by Samsung Pharm. Ltd. (e.g., 01, 02, 03…)
- Screening number: “S”, randomization number: “R”
- Stratification code (1=locally advanced, 2=metastatic) and control group* code (“3”)

* Indicates the low eotaxin group within the control group.

- Sequentially assigned number in the order of screening or in the order of randomization by stratification factor

A screening number does not contain a stratification code which is used only for the generation of a randomization.

For instance, the screening number of the first subject who provided informed consent at a site whose site code was 01 is 01S-01, and the randomization number of the first randomized subject at this site with locally advanced disease was 01R-**1**01. In addition, the number is to be 01R-**3**01 for a subject in the control group. A randomization list is generated by stratification factor, i.e., progression of pancreatic cancer (locally advanced, metastatic), using block randomization method in a manner to randomize subjects to either the study group or the control group in a 2:1 ratio. The randomization list was prepared only for those in the high eotaxin group; those in the low eotaxin group were assigned to the control group without randomization.

The ultimate ratio of allocation to the study group (high eotaxin) and the control group (high + low eotaxin) is to be maintained at 1:1. Randomization codes will be owned by a CRO, LSK global PS. Access to the codes will be limited to the site pharmacy.

## Subject Enrollment Center

In this study, subject enrollment is managed by subject enrollment center.

## Blinding

This was an open-label study, and therefore, blinding was not applied.

# EFFICACY AND SAFETY VARIABLES

## Schedules of Study Procedures

Schedules for study procedures and the assessment of efficacy and safety variables are presented in “Table 12.1.1 Schedules of study procedures”.

Table 12.1.1 Schedules of study procedures

**1) Control group (Gemcitabine/capecitabine) (Screening Visit ~ Week 20 Visit)**

|  | | **Screening** | | **Treatment period** | | | | | | | | | | | | | | | | | | | |
| --- | --- | --- | --- | --- | --- | --- | --- | --- | --- | --- | --- | --- | --- | --- | --- | --- | --- | --- | --- | --- | --- | --- | --- |
| **Visit** | | **1** | **2** | **3** | **4** | **5** | **-** | **6** | **7** | **8** | **9** | **10** | **11** | **12** | **-** | **13** | **14** | **15** | **-** | **16** | **17** | **18** | **19** |
| **Week** | | **-** | **-** | **1** | **2** | **3** | **4** | **5** | **6** | **7** | **8** | **9** | **10** | **11** | **12** | **13** | **14** | **15** | **16** | **17** | **18** | **19** | **20** |
| **Day** | | **≤21** | **≤7** | **1** | **8** | **15** | **22** | **29** | **36** | **43** | **50** | **57** | **64** | **71** | **78** | **85** | **92** | **99** | **106** | **113** | **120** | **127** | **134** |
| **Window period (days)** | | **-** | **-** | **-** | **±1** | **±1** | **-** | **±1** | **±1** | **±1** | **±1** | **±1** | **±1** | **±1** | **-** | **±1** | **±1** | **±1** | **-** | **±1** | **±1** | **±1** | **±1** |
| Histopathological/cytological confirmation of pancreatic cancer^[1]^ | | X |  |  |  |  |  |  |  |  |  |  |  |  |  |  |  |  |  |  |  |  |  |
| ICF and screening number | | X |  |  |  |  |  |  |  |  |  |  |  |  |  |  |  |  |  |  |  |  |  |
| Inclusion/exclusion criteria | |  | X |  |  |  |  |  |  |  |  |  |  |  |  |  |  |  |  |  |  |  |  |
| Randomization^[2]^ | |  | X |  |  |  |  |  |  |  |  |  |  |  |  |  |  |  |  |  |  |  |  |
| Demographic information, medical history, and medication history ^[3]^ | | X |  |  |  |  |  |  |  |  |  |  |  |  |  |  |  |  |  |  |  |  |  |
| Height and weight^[4]^ | |  | X | X |  |  |  | X |  |  |  | X |  |  |  | X |  |  |  | X |  |  |  |
| Physical examination* | |  | X | X |  |  |  | X |  |  |  | X |  |  |  | X |  |  |  | X |  |  |  |
| Vital signs* ^[5]^ | |  | X | X |  |  |  | X |  |  |  | X |  |  |  | X |  |  |  | X |  |  |  |
| ECOG * | |  | X | X |  |  |  | X |  |  |  | X |  |  |  | X |  |  |  | X |  |  |  |
| ECG | | X |  |  |  |  |  |  |  |  |  |  |  |  |  |  |  |  |  |  |  |  |  |
| Clinical laboratory tests* ^[6]^ | |  | X^#^ | X | X | X |  | X | X | X |  | X | X | X |  | X | X | X |  | X | X | X |  |
| Pregnancy test^[7]^ | |  | X |  |  |  |  |  |  |  |  |  |  |  |  |  |  |  |  |  |  |  |  |
| Central laboratory tests | Immunological monitoring  (blood sampling for MDSC) ^[8]^ |  |  | X^￥^ |  |  |  |  |  |  |  |  | X |  |  |  | X |  |  |  | X |  |  |
|  | Eotaxin test ^[9]^ | X |  |  |  |  |  |  |  |  |  |  | X |  |  |  | X |  |  |  | X |  |  |
| CT (tumor assessment)^[10]^ | | X |  |  |  |  |  |  |  |  | X |  |  |  |  |  |  |  |  |  |  |  | X |
| CA19-9 test* | |  | X |  |  |  |  | X |  |  | X |  |  |  |  |  | X |  |  |  |  |  |  |
| DTH test ^[11]^ | |  |  |  |  |  |  |  |  |  |  |  |  |  |  |  |  |  |  |  |  |  |  |
| Quality of life assessment | |  | X^#^ |  |  | X |  |  |  |  | X |  |  |  |  |  |  |  |  |  |  |  | X |
| Pain assessment | |  | X^#^ | Daily (diary) | | | | | | | | | | | | | | | | | | | |
| Concomitant medication/treatment | |  | X | X | X | X |  | X | X | X | X | X | X | X |  | X | X | X |  | X | X | X | X |
| Adverse events | |  |  | X | X | X |  | X | X | X | X | X | X | X |  | X | X | X |  | X | X | X | X |
| Gemcitabine treatment | |  |  | X | X | X |  | X | X | X |  | X | X | X |  | X | X | X |  | X | X | X |  |
| Capecitabine treatment | |  |  | X | X | X |  | X | X | X |  | X | X | X |  | X | X | X |  | X | X | X |  |
| GV1001/GM-CSF treatment | |  |  |  |  |  |  |  |  |  |  |  |  |  |  |  |  |  |  |  |  |  |  |
| Treatment compliance (capecitabine) | |  |  |  |  |  |  | X |  |  |  | X |  |  |  | X |  |  |  | X |  |  |  |

* Physical examination, vital signs, ECOG assessment, and clinical laboratory tests (hematology/blood chemistry/CA19-9) were to be performed within 3 days prior to the post-randomization visit.

# Clinical laboratory tests (hematology/blood chemistry), quality of life assessment, and pain assessment for screening (Visit 2) were to be performed prior to randomization.

￥ For MDSC testing, a blood sample was collected before gemcitabine dosing and at 48 hours (± 6 hours) after gemcitabine dosing (a total of 2 blood samples collected during Week 1). Therefore, subjects in the control group had **an additional on-site visit for blood sampling** for MDSC analysis after gemcitabine dosing.

**2) Control group (gemcitabine/capecitabine) (Week 21 Visit to EOS)**

|  | | **Treatment period** | | | | | | | | | | | |  | **End of Treatment** | **Follow-up^12^** | **End of study** |
| --- | --- | --- | --- | --- | --- | --- | --- | --- | --- | --- | --- | --- | --- | --- | --- | --- | --- |
| **Visit** | | **20** | **21** | **22** | **-** | **23** | **24** | **25** | **-** | **26** | **27** | **28** | **29** | **-** | **-** | **-** | **-** |
| **Week** | | **21** | **22** | **23** | **24** | **25** | **26** | **27** | **28** | **29** | **30** | **31** | **32** | **-** | **-** | **Every 12 weeks** | **-** |
| **Day** | | **141** | **148** | **155** | **162** | **169** | **176** | **183** | **190** | **197** | **204** | **211** | **218** | **-** | **-** | **-** | **-** |
| **Window period (days)** | | **±1** | **±1** | **±1** | **-** | **±1** | **±1** | **±1** | **-** | **±1** | **±1** | **±1** | **±1** | **-** | **Within 6 days after**  **IP discontinuation** | **±6** | **-** |
| Histopathological/cytological confirmation of pancreatic cancer^[1]^ | |  |  |  |  |  |  |  |  |  |  |  |  | **Treatment continued until PD** |  |  |  |
| ICF and screening number | |  |  |  |  |  |  |  |  |  |  |  |  |  |  |  |  |
| Inclusion/exclusion criteria | |  |  |  |  |  |  |  |  |  |  |  |  |  |  |  |  |
| Randomization ^[2]^ | |  |  |  |  |  |  |  |  |  |  |  |  |  |  |  |  |
| Demographic information, medical history, and medication history ^[3]^ | |  |  |  |  |  |  |  |  |  |  |  |  |  |  |  |  |
| Height and weight^[4]^ | | X |  |  |  | X |  |  |  | X |  |  |  |  | X | X |  |
| Physical examination* | | X |  |  |  | X |  |  |  | X |  |  |  |  | X | X |  |
| Vital signs*^[5]^ | | X |  |  |  | X |  |  |  | X |  |  |  |  | X | X |  |
| ECOG * | | X |  |  |  | X |  |  |  | X |  |  | X |  | X | X |  |
| ECG | |  |  |  |  |  |  |  |  |  |  |  |  |  |  |  |  |
| Clinical laboratory tests* ^[6]^ | | X | X | X |  | X | X | X |  | X | X | X |  |  | X |  |  |
| Pregnancy test^[7]^ | |  |  |  |  |  |  |  |  |  |  |  |  |  |  |  |  |
| Central laboratory tests | Immunological monitoring  (blood sampling for MDSC) ^[8]^ |  |  |  |  |  |  |  |  |  |  |  |  |  |  |  |  |
|  | Eotaxin test ^[9]^ |  |  |  |  |  |  |  |  |  |  |  |  |  |  |  |  |
| CT (tumor assessment) ^[10]^ | |  |  |  |  |  |  |  |  |  |  |  | X |  |  | X¶ |  |
| CA19-9 test* | |  | X |  |  |  | X |  |  |  | X |  |  |  |  | X |  |
| DTH test ^[11]^ | |  |  |  |  |  |  |  |  |  |  |  |  |  |  |  |  |
| Quality of life assessment | |  |  |  |  |  |  |  |  |  |  |  | X |  |  | X |  |
| Pain assessment | | Daily (diary) | | | | | | | | | | | |  |  |  |  |
| Concomitant medication/treatment | | X | X | X |  | X | X | X |  | X | X | X | X |  | X | X |  |
| Adverse events | | X | X | X |  | X | X | X |  | X | X | X | X |  | X | X | X |
| Gemcitabine treatment | | X | X | X |  | X | X | X |  | X | X | X |  |  |  |  |  |
| Capecitabine treatment | | X | X | X |  | X | X | X |  | X | X | X |  |  |  |  |  |
| GV1001/GM-CSF treatment | |  |  |  |  |  |  |  |  |  |  |  |  |  |  |  |  |
| Treatment compliance (capecitabine) | | X |  |  |  | X |  |  |  | X |  |  |  |  |  |  |  |
| Reason for withdrawal checking | |  |  |  |  |  |  |  |  |  |  |  |  |  | X |  |  |
| Survival/death follow-up | |  |  |  |  |  |  |  |  |  |  |  |  |  |  | X | X |

* Physical examination, vital signs, ECOG assessment, and clinical laboratory tests (hematology/blood chemistry/CA19-9) were to be performed within 3 days prior to the post-randomization visit.

# Clinical laboratory tests (hematology/blood chemistry), quality of life assessment, and pain assessment for screening (Visit 2) were to be performed prior to randomization.

¶ CT scanning (or MRI) was performed only in subjects without PD at EOT.

**3) Study group (GV1001+gemcitabine/capecitabine) (Screening Visit ~ Week 20 Visit)**

|  | | **Screening** | | **Treatment period** | | | | | | | | | | | | | | | | | | | | | | | | | | | |
| --- | --- | --- | --- | --- | --- | --- | --- | --- | --- | --- | --- | --- | --- | --- | --- | --- | --- | --- | --- | --- | --- | --- | --- | --- | --- | --- | --- | --- | --- | --- | --- |
| **Visit** | | **1** | **2** | **3** | **4** | **5** | **6** | **7** | **8** | **9** | **10** | **11** | **12** | **13** | **14** | **15** | **-** | **16** | **17** | | **18** | | **-** | | **19** | | **20** | | **21** | | **22** |
| **Week** | | **-** | **-** | **1** | **1** | **1** | **2** | **3** | **4** | **5** | **6** | **7** | **8** | **9** | **10** | **11** | **12** | **13** | **14** | | **15** | | **16** | | **17** | | **18** | | **19** | | **20** |
| **Day** | | **≤21** | **≤7** | **1** | **3** | **5** | **8** | **15** | **22** | **29** | **36** | **43** | **50** | **57** | **64** | **71** | **78** | **85** | **92** | | **99** | | **106** | | **113** | | **120** | | **127** | | **134** |
| **Window period (days)** | | **-** | **-** | **-** | **±1** | **±1** | **±4** | **±4** | **±4** | **±4** | **±4** | **±4** | **±4** | **±4** | **±4** | **±4** | **-** | **±4** | **±4** | | **±4** | | **±4** | | **±4** | | **±4** | | **±4** | | **±4** |
| Histopathological/cytological confirmation of pancreatic cancer^[1]^ | | X |  |  |  |  |  |  |  |  |  |  |  |  |  |  |  |  |  | |  | |  | |  | |  | |  | |  |
| ICF and screening number | | X |  |  |  |  |  |  |  |  |  |  |  |  |  |  |  |  |  | |  | |  | |  | |  | |  | |  |
| Inclusion/exclusion criteria | |  | X |  |  |  |  |  |  |  |  |  |  |  |  |  |  |  |  | |  | |  | |  | |  | |  | |  |
| Randomization^[2]^ | |  | X |  |  |  |  |  |  |  |  |  |  |  |  |  |  |  |  | |  | |  | |  | |  | |  | |  |
| Demographic information, medical history, and medication history collected^[3]^ | | X |  |  |  |  |  |  |  |  |  |  |  |  |  |  |  |  |  | |  | |  | |  | |  | |  | |  |
| Height and weight^[4]^ | |  | X | X |  |  |  |  |  | X |  |  |  | X |  |  |  | X |  | |  | |  | | X | |  | |  | |  |
| Physical examination* | |  | X | X |  |  |  |  |  | X |  |  |  | X |  |  |  | X |  | |  | |  | | X | |  | |  | |  |
| Vital signs* ^[5]^ | |  | X | X |  |  |  |  |  | X |  |  |  | X |  |  |  | X |  | |  | |  | | X | |  | |  | |  |
| ECOG * | |  | X | X |  |  |  |  |  | X |  |  |  | X |  |  |  | X |  | |  | |  | | X | |  | |  | |  |
| ECG | | X |  |  |  |  |  |  |  |  |  |  |  |  |  |  |  |  |  | |  | |  | |  | |  | |  | |  |
| Clinical laboratory tests* ^[6]^ | |  | X | X |  |  | X | X |  | X | X | X |  | X | X | X |  | X | X | | X | |  | | X | | X | | X | |  |
| Pregnancy test^[7]^ | |  | X |  |  |  |  |  |  |  |  |  |  |  |  |  |  |  |  | |  | |  | |  | |  | |  | |  |
| Central laboratory tests | Immunological monitoring  (blood sampling for T-Cell and MDSC) ^[8]^ |  |  | X | X^￥^ |  |  |  |  |  |  |  |  |  | X |  |  |  | X | |  | |  | |  | | X | |  | |  |
|  | Eotaxin test ^[9]^ | X |  |  |  |  |  |  |  |  |  |  |  |  | X |  |  |  | X | |  | |  | |  | | X | |  | |  |
| CT (tumor assessment) ^[10]^ | | X |  |  |  |  |  |  |  |  |  |  | X |  |  |  |  |  |  | |  | |  | |  | |  | |  | | X |
| CA19-9 test* | |  | X |  |  |  |  |  |  | X |  |  | X |  |  |  |  |  | X | |  | |  | |  | |  | |  | |  |
| DTH test ^[11]^ | |  |  | X |  |  |  |  | X |  | X |  |  |  | X |  |  |  |  | |  | |  | |  | | X | |  | |  |
| Quality of life assessment | |  | X |  |  |  |  | X |  |  |  |  | X |  |  |  |  |  |  | |  | |  | |  | |  | |  | | X |
| Pain assessment | |  | X | Daily (diary) | | | | | | | | | | | | | | | | | | | | | | | | | | | |
| Concomitant medication/treatment | |  | X | X | X | X | X | X | X | X | X | X | X | X | X | X |  | X | X | X | |  | | X | | X | | X | | X | |
| Adverse events | |  |  | X | X | X | X | X | X | X | X | X | X | X | X | X |  | X | X | X | |  | | X | | X | | X | | X | |
| Gemcitabine treatment | |  |  | X |  |  | X | X |  | X | X | X |  | X | X | X |  | X | X | X | |  | | X | | X | | X | |  | |
| Capecitabine treatment | |  |  | X | X | X | X | X |  | X | X | X |  | X | X | X |  | X | X | X | |  | | X | | X | | X | |  | |
| GV1001/GM-CSF Treatment | |  |  | X | X | X | X | X | X |  | X |  |  |  | X |  |  |  | X |  | |  | |  | | X | |  | |  | |
| Treatment compliance (capecitabine) | |  |  |  |  |  |  |  |  | X |  |  |  | X |  |  |  | X |  |  | |  | | X | |  | |  | |  | |

* Physical examination, vital signs, ECOG assessment, and clinical laboratory tests (hematology/blood chemistry/CA19-9) were to be performed within 3 days prior to the post-randomization visit.

# Clinical laboratory tests (hematology/blood chemistry), quality of life assessment, and pain assessment for screening (Visit 2) were to be performed prior to randomization.

￥ For immunological monitoring (T-cell proliferation/MDSC), a blood sample was collected at 48 (± 6 hours) after gemcitabine dosing at Week 1.

**4) Study group (GV1001+gemcitabine/capecitabine) (Week 21 Visit to EOS)**

|  | | **Treatment period** | | | | | | | | | | | |  | **End of Treatment** | **Follow-up^12^** | **End of study** |
| --- | --- | --- | --- | --- | --- | --- | --- | --- | --- | --- | --- | --- | --- | --- | --- | --- | --- |
| **Visit** | | **23** | **24** | **25** | **-** | **26** | **27** | **28** | **-** | **29** | **30** | **31** | **32** | **-** | **-** | **-** | **-** |
| **Week** | | **21** | **22** | **23** | **24** | **25** | **26** | **27** | **28** | **29** | **30** | **31** | **32** | **-** | **-** | **Every 12 weeks** | **-** |
| **Day** | | **141** | **148** | **155** | **162** | **169** | **176** | **183** | **190** | **197** | **204** | **211** | **218** | **-** | **-** | **-** | **-** |
| **Window period (days)** | | **±4** | **±4** | **±4** | **-** | **±4** | **±4** | **±4** | **-** | **±4** | **±4** | **±4** | **±4** | **-** | **Within 6 days after IP discontinuation** | **±6** | **-** |
| Histopathological/cytological confirmation of pancreatic cancer^[1]^ | |  |  |  |  |  |  |  |  |  |  |  |  | **Treatment continued until PD** |  |  |  |
| ICF and screening number | |  |  |  |  |  |  |  |  |  |  |  |  |  |  |  |  |
| Inclusion/exclusion criteria | |  |  |  |  |  |  |  |  |  |  |  |  |  |  |  |  |
| Randomization^[2]^ | |  |  |  |  |  |  |  |  |  |  |  |  |  |  |  |  |
| Demographic information, medical history, and medication history ^[3]^ | |  |  |  |  |  |  |  |  |  |  |  |  |  |  |  |  |
| Height and weight^[4]^ | | X |  |  |  | X |  |  |  | X |  |  |  |  | X | X |  |
| Physical examination* | | X |  |  |  | X |  |  |  | X |  |  |  |  | X | X |  |
| Vital signs* ^[5]^ | | X |  |  |  | X |  |  |  | X |  |  |  |  | X | X |  |
| ECOG * | | X |  |  |  | X |  |  |  | X |  |  | X |  | X | X |  |
| ECG | |  |  |  |  |  |  |  |  |  |  |  |  |  |  |  |  |
| Clinical laboratory tests* ^[6]^ | | X | X | X |  | X | X | X |  | X | X | X |  |  | X |  |  |
| Pregnancy test^[7]^ | |  |  |  |  |  |  |  |  |  |  |  |  |  |  |  |  |
| Central laboratory tests | Immunological monitoring  (blood sampling for T-Cell and MDSC) ^[8]^ |  |  |  |  |  |  |  |  |  |  |  |  |  |  |  |  |
|  | Eotaxin test ^[9]^ |  |  |  |  |  |  |  |  |  |  |  |  |  |  |  |  |
| CT (tumor assessment) ^[10]^ | |  |  |  |  |  |  |  |  |  |  |  | X |  |  | X¶ |  |
| CA19-9 test* | |  | X |  |  |  | X |  |  |  | X |  |  |  |  | X |  |
| DTH test ^[11]^ | |  |  |  |  |  |  |  |  |  |  |  |  |  |  |  |  |
| Quality of life assessment | |  |  |  |  |  |  |  |  |  |  |  | X |  |  | X |  |
| Pain assessment | | Daily (diary) | | | | | | | | | | | |  |  |  |  |
| Concomitant medication/treatment | | X | X | X |  | X | X | X |  | X | X | X | X |  | X | X |  |
| Adverse events | | X | X | X |  | X | X | X |  | X | X | X | X |  | X | X | X |
| Gemcitabine treatment | | X | X | X |  | X | X | X |  | X | X | X |  |  |  |  |  |
| Capecitabine treatment | | X | X | X |  | X | X | X |  | X | X | X |  |  |  |  |  |
| GV1001/GM-CSF treatment | |  | X |  |  |  | X |  |  |  | X |  |  |  |  |  |  |
| Treatment compliance (capecitabine) | | X |  |  |  | X |  |  |  | X |  |  |  |  |  |  |  |
| Reason for withdrawal checking | |  |  |  |  |  |  |  |  |  |  |  |  |  | X |  |  |
| Survival/death follow-up | |  |  |  |  |  |  |  |  |  |  |  |  |  |  | X | X |

* Physical examination, vital signs, ECOG assessment, and clinical laboratory tests (hematology/blood chemistry/CA19-9) were to be performed within 3 days prior to the post-randomization visit.

# Clinical laboratory tests (hematology/blood chemistry), quality of life assessment, and pain assessment for screening (Visit 2) were to be performed prior to randomization.

¶ CT scanning (or MRI) was performed only in subjects without PD at EOT.

1. Histopathological/cytological confirmation of pancreatic cancer: confirmed before enrollment (no time limit for testing and determination)
2. Randomization: by IWRS (randomization could be performed on the same day as Visit 3.)
3. Demographic information, medical history, and medication history collected: 1) Demographic information: gender, age, pancreas related family history (pancreatitis, pancreatic cancer), 2) Medical history: medical history within 6 months prior to the screening visit (including surgical history; for malignant tumors, within 5 years prior to the screening visit), 3) Medication history: medication history within 3 months prior to the screening visit.
4. Height and weight: height was measured at screening only.
5. Vital signs: sitting systolic blood pressure (SBP)/diastolic blood pressure (DBP), pulse, respiratory rate, body temperature were measured.
6. Clinical laboratory tests:

- Hematology: RBC, Hb, Hct, Platelet, WBC, WBC differential count (Neutrophils, Lymphocytes, Monocytes, Eosinophils, Basophils)
- Blood chemistry: Albumin, Total Protein, Total Cholesterol, BUN, Creatinine, ALT, AST, Total Bilirubin, Alkaline Phosphatase, γ-GT, Na, K, Ca, P, CRP
- Urinalysis: urine microscopy (RBC, WBC), pH, Blood, Glucose, Urobilinogen, Ketone, Albumin(protein), Bacteria
- Immunoassay (at screening and EOT): HBsAg, Anti-HCV, HIV

* If a laboratory test value was out of the normal range, a re-test was permitted at the discretion of the investigator.

** For CCr at screening for the inclusion criterion checking, a value calculated by Cockcroft-gault using the plasma creatinine value at the site was used.

CCr={(140-age) X weight)/(72 X plasma creatinine)} (X 0.85 for women only)

1. Pregnancy test: at screening (by urinalysis; or serum testing if not feasible by urinalysis) and as necessary thereafter.
2. For immunological monitoring (T-cell proliferation/MDSC), 16 mL of blood was collected in 2 CPT tubes (8 mL/tube) and shipped to the central laboratory for the study group; and 8 mL of blood was collected in a CPT tube (8 mL/tube) and shipped to the central laboratory for the control group.
3. Eotaxin test: 4 mL of blood was collected and shipped to the central laboratory. The result was confirmed prior to randomization.
4. CT scan: The result was determined by the central reading center. Post-enrollment CT scanning was done within 7 days from the scheduled visit. An additional CT scanning was permitted at the discretion of the investigator provided it was essential to have another test. If CT scanning was unavailable, MRI could be performed.
5. DTH test: only in the GV1001+gemcitabine/capecitabine group (study group). The test was done at a site different from the IP dosing site. A red spot no smaller than 5 mm * 5 mm (mean ≥5 mm, 5+5/2) was determined positive. If it was difficult to determine the size, a spot with the smallest diameter of ≥3 mm was determined positive (e.g., positive for 3 mm * 7 mm; negative for 2 mm * 8 mm).
6. Follow-up: The purpose of follow-up visits was to check survival. If the subject did not return for an on-site visit, survival was checked by telephone monitoring. If the subject had an on-site visit, available data were collected.

## Observations

### Obtain Informed Consent and Assign a Screening Number

Before entering the clinical trial, after explaining the purpose and contents of this clinical trial in detail to the subject (or his/her representative), consent is obtained in writing. Written consent must be obtained prior to any procedure in the clinical trial, and the investigator must provide the subject with a copy of the signed ‘subject explanation and signed consent form’.

After obtaining written consent from the subject, the investigator assigns a screening number in the order in which consent is obtained. If a subject assigned a screening number is withdrawn from screening, the corresponding code will also be rejected and will not be reassigned to another subject.

### Demographic Data

Demographic information of patients selected as potential subjects for clinical trials is collected at the time of screening and includes the subject's initials, gender, age, and family history of pancreatic-related diseases (eg, pancreatitis, pancreatic cancer).

### Histopathological/Cytological Diagnostic Information

Histopathological information can be collected via laparotomy, laparoscopic or percutaneous biopsy at the primary cancer or secondary site (liver, lung, regional lymph node, mesentery, or peritoneum).

Cytological information can be collected by fine needle aspiration biopsy performed laparoscopically or ultrasound in the primary cancer or secondary site (liver, lung, regional lymph node, mesentery, or peritoneum with ascites). It may also be collected through endoscopic retrograde cholangiopancreatography (ERCP) or percutaneous procedures in the main pancreatic duct and/or main bile duct.

### Medical, Surgical, and Treatment History of Pancreatic Cancer

Information about the subject's medical history, surgical history, and treatment history of pancreatic cancer is collected at the screening visit. All relevant medical history within 6 months (5 years for malignant tumors) prior to screening should be reviewed. The treatment history of pancreatic cancer includes the date of diagnosis, diagnosis results (refer to Section 12.2.1.2, ‘Hitopathological or cytological diagnosis information’), metastasis (other organs, lymph nodes, etc.), and surgical history.

### Drug Administration History

Through the questionnaire and review of past medical records, the drugs taken within 3 months of the screening visit are investigated in detail and recorded in the eCRF.

For drug administration history, the drug name (ingredient name), administration purpose, daily dose, administration route, and administration period of the drug administered to the subject within 3 months prior to the screening visit are investigated.

### Chest, Abdomen and Pelvis CT scan (or MRI)

CT scanning (or MRI) on the chest, abdomen, and pelvis is performed in all treatment groups at screening, Week 8, and every 12 weeks thereafter until EOT. **However, additional CT scans (or MRI) can be performed if additional CT scans are clearly required at the discretion of the investigator.** For those without documented PD at EOT, CT scans are obtained at follow-up visits scheduled every 12 weeks. For the last subject at the final EOS, CT scanning (or MRI) is performed at Week 52 of treatment and/or follow-up for final tumor assessment.

CT scan (or MRI) are read by the central reading center. The CT scan (or MRI) data obtained at the site are sent to the central reading center, and the reading results are to be delivered to the site as soon as possible. If CT scanning is not possible, MRI can be performed.

If possible, the testing method performed at screening (CT scanning or MRI) is maintained throughout the study. For those who can not be administered with contrast for CT scanning (or MRI) due to allergy to contrast, non-contrast enhanced CT scanning (or MRI) is performed. Subjects for whom CT scanning is unavailable are allowed to be enrolled if it is possible to perform tumor assessment by MRI.

Baseline CT scanning (or MRI): Baseline CT scanning on the chest, abdomen, and pelvis is performed within 28 days (up to 32 days) prior to initiation of IP dosing.

All CT scanning (or MRI) after enrollment: performed within **7 days** from the scheduled visit.

As for the CT scan (or MRI) reading result, the objective response rate will be evaluated through RECIST and irRC. Refer to Appendix 4 for RECIST and irRC standards.

### Immune Response Assessment

1. T-cell proliferation

T-cell proliferation is tested only in the study group. Before gemcitabine dosing, approximately 16 mL of blood is collected (in 2 CPT tubes; 8 mL/tube) and shipped to the central laboratory. Time points for blood sampling are: before the first IP (gemcitabine) dosing (Visit 3), 48 hours after the first gemcitabine dosing at Week 1 (Visit 4), and before gemcitabine dosing at Weeks 10, 14, and 18.

Protocols for collecting, processing, shipping, and handling of samples are provided in separate laboratory manuals.

T-cell proliferation testing is performed as follows according to site-specific standard operating procedures (SOPs).

As Method 1, peripheral blood mononuclear cells (PBMCs) are stimulated with GV1001 and treated with [3H]-thymidine. The level of [3H]-thymidine bound to the DNA of T cells are measured by cell harvester, OR

As Method 2, T cells are isolated from PBMCs and treated with 5,6-carboxyfluoroscein diacetate succinimidyl ester (CFSE). T cell proliferation is measured by flow cytometer.

1. Delayed-type hypersensitivity (DTH) testing

To confirm the induction of immune response, DTH is tested only in the study group after IP dosing. The test is performed after the first dose of the IP (Visit 3) and Weeks 4, 6, 10, and 18. Once a positive outcome is obtained, DTH is no longer tested at the remaining scheduled time points. If DTH is tested positive at Visit 3 (first DTH test outcome), DTH testing is conducted until the next positive outcome is obtained to confirm activated immune response.

DTH testing is prepared by the pharmacist or a person in charge of preparation of GV1001. After GV1001 dose is prepared, approximately 0.08 mL of the remaining solution (Solution A) is diluted with 0.22 mL of the provided saline for IV injection (0.09% w/v) to obtain approximately 0.7 mg/mL GV1001 (Solution B).

After ID doses of GM-CSF and the IP (GV1001), 0.15 mL (0.7 mg/ mL) of Solution B is given as an ID injection for DTH testing (see Figure 12.2.1.). ‘Solution B’ is administered to lower abdomen, opposite to the site of GV1001 administration, within 6 hours after preparation. GV1001 dosing is not affected by dosing sequences and time intervals with DTH testing.


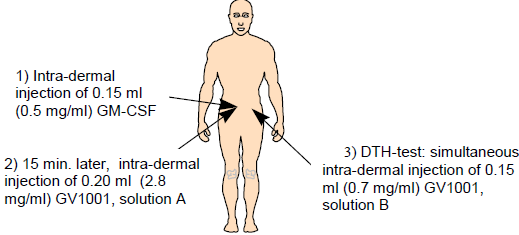


**Within 10-15 min., intra-dermal**

**Figure 12.2.1 Injection sites**

The subject will be instructed to measure with a ruler the size of a red spot appearing at the site of the ID injection for DTH testing approximately 48 hours after the test and to document the measurement in the subject diary. The value should be checked at the next visit and recorded in eCRF.

### QoL Assessment

Quality of life assessments are performed at the screening visit, at Weeks 3, 8, and every 12 weeks thereafter (see Appendix 4). EORTC QLQ-C30 and EQ-5D-3L, which are self-written questionnaires, are used as research tools, and scores are calculated and evaluated based on the subject's responses to the questionnaire. The investigator collects the questionnaire after checking that there is no omission of answers to all questionnaires.

1) EORTC QLQ-C30

The EORTC QLQ-C30 consists of 2 questions measuring overall quality of life, 15 questions measuring 5 functional scales (physical, role, cognitive, emotional, and social function), 7 questions measuring 3 symptom scales (Fatigue, pain and nausea/vomiting) and additionally, 1 question each for dyspnea, anorexia, sleep disturbance, constipation, diarrhea and economic difficulties, which are commonly reported symptoms in cancer patients. The questions measuring the overall quality of life consist of a 7-point scale, and all other questions except for this are composed of a 4-point scale. The higher the overall quality of life and functional scale score, and the lower the symptom scale score, the higher the quality of life.

2) EQ-5D-3L

EQ-5D-3L consists of five multiple-choice questions asking about current health status and one question (Visual Analogue Scale, VAS) indicating subjective health level (0-100 points). The five multiple-choice questions are composed of the areas of exercise ability, self-management, daily life, pain/discomfort, and anxiety/depression. The investigator collects the questionnaire after confirming that there are no omissions in answers to a total of 5 questions and VAS questions.

### Pain Intensity Assessment

All subjects are evaluated for pain level at the screening visit (Visit 2) and daily from the start date of investigational drug administration (Visit 3) to the end of treatment. Subjects evaluate and describe the pain level on a separately provided record sheet, and the investigator checks the pain level through the pain record sheet written by the subject every day after the screening visit (Visit 2). The survey tool for pain assessment is the VAS, which indicates the degree of pain during the day of the assessment from 0 (no pain) to 100 mm (unbearable pain) (see Appendix 8).

### Concomitant Drug

If the subject is taking or undergoing treatment at the time of trial participation (Visit 1), it should be recorded as a concomitant drug. In the event that concomitant drugs (including drugs in the event of other diseases or adverse reactions) and treatment regimens are changed or added thereafter, drug information (ingredient name, administration purpose, dose, administration period, etc.) and treatment regimen should be recorded in eCRF in detail.

Frequent concomitant administration of high-dose steroids as antiemetics is prohibitid because it may have a detrimental effect on the response to vaccine administration. Since nausea and vomiting are not severe when gemcitabine and capecitabine are combined, nonsteroidal administration is recommended.

### Adverse Events

Adverse event evaluation includes any signs, symptoms, disorders, or diseases that appear or worsen in a subject during the treatment phase and that may impair the patient's well-being. In addition, evaluation should include laboratory findings or results of other diagnostic procedures considered clinically significant (eg, resulting in unscheduled diagnostic procedures or treatment modalities or discontinuation of the study).

Adverse events are evaluated and collected at every visit from the start date of administration of the investigational drug (Day 1). Signs, symptoms or diseases collected before the start of administration of the investigational product are the criteria for evaluating all adverse events occuring after administration of the investigational product. However, diagnosis of progressive disease (PD) is not reported as an adverse event, and death due to disease progression is not reported as a serious adverse event.

For details on the evaluation criteria, methods, and reporting procedures for adverse reactions, refer to Section 16 “CRITERIA, METHODS AND REPORTING OF SAFETY ASSESSSMENT INCLUDING ADVERSE EVENTS”.

### Clinical Laboratory Tests

Samples for clinical laboratory tests are collected at each visit when gemcitabine/capecitabine are administered and are assessed at the laboratory of each site. Test parameters are as follows. If a laboratory test value is out of the normal range, a re-test can be performed at the discretion of the investigator only for the relevant test for which a re-visit is carried out.

- Hematology: RBC, Hb, Hct, Platelet, WBC, WBC differential count (Neutrophils, Lymphocytes, Monocytes, Eosinophils, Basophils)
- Blood chemistry: Albumin, Total Protein, Total Cholesterol, BUN, Creatinine (CCr*), ALT, AST, Total Bilirubin, Alkaline Phosphatase, γ-GT, Na, K, Ca, P, CRP

* For CCr, a value calculated by Cockcroft-gault using a serum creatinine value was used.

CCr={(140-age) X weight)/(72 X plasma creatinine)} (X 0.85 for women only)

- Urinalysis: urine microscopy (RBC, WBC), pH, Blood, Glucose, Urobilinogen, Ketone, Albumin(protein), Bacteria
- Immunoassay (at screening [Visit 2] and EOT): HBsAg, Anti-HCV, HIV
- Biomarkers: CA 19-9 (at screening [Visit 2], Weeks 5, 8, 14, 22, 26, 30, and all follow-up visits)

For immune response testing, separate samples are collected besides samples for clinical laboratory tests and shipped to the central laboratory.

- Eotaxin measurement (4 mL of blood collected in an SST tube): at the screening visit (Visit 1) and Weeks 10, 14, and 18
- T-cell proliferation and MDSC (16 mL of blood collected in 2 CPT tubes)*: at the baseline visit (Visit 3), 48 hours after gemcitabine dosing at Week 1 (Visit 4), and Weeks 10, 14, and 18

*For the control group, T-cell proliferation is not tested, and blood samples are collected only for MDSC testing (8 mL of blood collected in 1 CPT tube). For MDSC testing, as no regular visit is scheduled 48 hours after gemcitabine dosing at Week 1, a separate visit is carried out for blood sampling.

### Vital Signs

For vital signs, sitting SBP/DBP, pulse, respiratory rate, and body temperature are measured and documented at screening (Visit 2), Visit 3 (prior to IP dosing), Weeks 5, 9, 13, 17, 21, 25, and 29, EOT visit, and all follow-up visits.

Blood pressure (BP) and pulse are measured after the subject had a rest for 5 minutes in a sitting position. If possible, BP is measured on the same arm.

### Physical Examination

Physical examination is conducted at screening (Visit 2), Visit 3 (prior to IP dosing), Weeks 5, 9, 13, 17, 21, 25, and 29, EOT visit, and all follow-up visits. Physical examination included examination of overall appearance: eye, ears, nose, pharynx/larynx, abdomen, skin/mucosa, lymphatic system, cardiovascular system, respiratory system, nervous system, and musculoskeletal system.

Any post-dose findings of physical examination that meets the definition of AEs should be reported as AEs.

### Height and Weight

Height (in cm) is measured only at the screening visit. Body weight (in kg) is measured at screening (Visit 2), Visit 3 (prior to IP dosing), Weeks 5, 9, 13, 17, 21, 25, and 29, EOT visit, and all follow-up visits.

### ECOG Performance status

ECOG performance status is assessed in all subjects at screening (Visit 2), Visit 3 (prior to IP dosing), Weeks 5, 9, 13, 17, 21, 25, 29, and 32, EOT visit, and all follow-up visits (see Appendix 2).

### ECG

ECG is performed at the screening visit (Visit 1). Afterwards, additional ECG is performed during the course of the study as necessary at the discretion of the investigator.

### Pregnancy Test

Except for those who are confirmed postmenopausal (≥1 year since the last menstruation) or surgically sterile at the screening visit (Visit 2), women of childbearing potential are tested for pregnancy by urine beta HCG (if not feasible, by serum testing) at the screening visit (Visit 2). Afterward, additional testing is to be performed as necessary during the course of the study.

### Randomization

When the subject is finally judged to meet the inclusion/exclusion criteria at the screening visit (Visit 2), the subject in the eotaxin high group are randomly assigned to the study group (GV1001 plus gemcitabine/capecitabine) and control group (Gemcitabine/capecitabine) according to the randomization method. They are randomized in 2:1 ratio, and subjects in the eotaxin low group are assigned to the control group in the same ratio as the control group in the high group. Therefore, in this study, the ratio between the study group and the control group is maintained at 1:1.

### Investigational Product Prescription

Subjects enrolled in this study will be prescribed investigational products according to their assigned group. Investigational products are administered as specified in the trial schedule in Section 12.1, and refer to Section 11.3 for the dosage, administration method, and administration period of investigational products.

### Compliance Check

All subjects must return the unused capecitabine prescribed in the previous anticancer cycle on the first day of each anticancer cycle after capecitabine administration. Investigators (or a person authorized to do so) check the information of amount of capecitabine prescribed and returned, and record it in the eCRF.

### End of Treatment (EOT) and End of Study (EOS)

In the event of termination of treatment (EOT) of investigational products or termination of this study (EOS), the reason shall be specified in the eCRF.

### Unscheduled visit

If medical treatment is required due to abnormalities in the test results performed before the end of treatment (EOT), or if the subject visited on a date other than the scheduled date due to an adverse event, the relevant information should be recorded in the relevant form. Regular visits must not be rescheduled due to unscheduled visits. If the unscheduled visit is due to treatment discontinuation, the examination and investigation scheduled for the end-of-treatment visit (EOT) should be conducted whenever possible. Unscheduled visits include phone visits.

### Follow-up

Subjects who have ended treatment (EOT) until the end of this study will be followed up for survival every 12 weeks. When follow-up is terminated before death is confirmed, the last time the subject was observed is the censoring time point. If the subject does not visit the relevant institution for follow-up visits, at least make it possible to check whether the subject is alive or not through telephone monitoring or in writing (eg, medical records, etc.). Scheduled examinations and investigations are performed at the follow-up visit, and if the subject has not been diagnosed with progressive disease (PD) at the end of treatment (EOT), a CT scan (or MRI) is performed at the follow-up visit every 12 weeks. In addition, at the end of this study, adverse events and survival of all subjects who are undergoing follow-up are finally confirmed.

In addition to checking for survival, the following additional data should be collected.

- Information on chemotherapy conducted after the end of treatment (EOT) (e.g., type of chemotherapy, radiation, etc., administrated drug, treatment period)

All adverse events occurring within 30 days of the end of treatment (EOT) should be recorded immediately, and in case of serious adverse events, it has to be reported to the sponsor. In the case of an adverse event occurring more than 30 days after the end of treatment, if the investigator determines that the relationship with the investigational product cannot be excluded, the event should be reported according to the adverse events reporting procedure.

Adverse reactions occurring during the treatment period should be followed-up until the investigator decides that follow-up is unnecessary because there is no further symptom change or recovery or until other chemotherapy is received.

For the confirmation and follow-up of adverse events occurring within 30 days after the end of treatment (EOT), clinical pathology tests or electrocardiography may be performed if necessary according to the clinical judgment of the investigator.

## Detailed Schedule for the Trial

For the detailed schedule of clinical trial progress by visit, refer to Section 12.1, “Schedules of Study Procedures”.

# EXPECTED ADVERSER EVENTS AND CAUTION FOR USE

## GV1001

### Expected Adverse Events

1) Mild and transient injection site reactions (eg erythema, soreness and pain) that are common after vaccination may occur.

2) The table below shows all adverse events that occurred in more than 10% of 1,062 subjects of Phase III pancreatic cancer study (TeloVac) on the control group (gemcitabine/capecitabine administration group) (N=358) and study group (GV1001 administration group) (N=354). Frequency is defined as: very common (≥1/10); common (≥1/100 to <1/10); not common (≥1/1,000 to <1/100), rarely (≥10,000 to <1/1,000), very rarely (<1/10,000).

| Adverse events seen in more than 10% of subjects in the phase III clinical trial for pancreatic cancer  (number of patients (%)) | | | |
| --- | --- | --- | --- |
|  | Control group  (Gemcitabine/Capecitabine)  (N=358) | Study group  (Gemcitabine/Capecitabine/  Leukine/GV1001)  (N=354) | Note |
| **Blood and lymphatic system disorders** | | | |
| Hemoglobin decrease | 91(25.4) | 73(20.6) | Very common |
| Neutropenia/granulopenia | 98(27.4) | 104(29.4) | Very common |
| Thrombocytopenia | 80(22.3) | 89(25.1) | Very common |
| **General disorders and administration site conditions** | | | |
| Fatigue | 223(62.3) | 205(57.9) | Very common |
| Fever | 55(16.5) | 68(19.2) | Very common |
| **Skin and subcutaneous tissue disorders** | | | |
| Rash | 71(19.8) | 67(18.9) | Very common |
| Injection site reaction | 1(0.27) | 94(26.6) | Very common |
| Rash – Hand and foot | 107(29.9) | 71(20.1) | Very common |
| **Gastrointestinal disorders** | | | |
| Appetite decrease | 110(30.7) | 99(28.0) | Very common |
| Constipation | 133(37.2) | 125(35.3) | Very common |
| Diarrhea | 149(41.6) | 131(37.0) | Very common |
| Stomatitis | 88(24.6) | 79(22.3) | Very common |
| Nausea | 180(50.3) | 200(56.5) | Very common |
| Gastrointestinal obstruction | 28(7.8) | 36(10.2) | Very common |
| Vomiting | 122(34.1) | 149(42.1) | Very common |
| **Infections and infestations** | | | |
| Infection | 40(11.2) | 30(8.5) | Very common |
| Infection with unkn own neutrophil count | 75(20.9) | 69(19.5) | Very common |
| **Lymph node** | | | |
| Arm edema | 75(20.9) | 83(23.4) | Very common |
| **Metabolism and nutrition disorders** | | | |
| ALT increase | 36(10.1) | 25(7.1) | Very common |
| **Nervous system disorders** | | | |
| Dizziness | 27(7.5) | 39(11.0) | Very common |
| Mood swings | 40(11.2) | 38(10.7) | Very common |
| **Pain** | | | |
| Pain | 207(57.8) | 211(59.6) | Very common |
| Pain in other parts | 53(14.8) | 45(12.7) | Very common |
| **Respiratory, thoracic and mediastinal adisorders** | | | |
| Shortness of breath | 49(13.7) | 50(14.1) | Very common |
| **Vascular disorders** | | | |
| Thrombosis/embolism | 50(14.0) | 41(11.6) | Very common |

Adverse events seen in less than 10% of patients were febrile neutropenia, leukopenia, insomnia, chills, sweating, weight loss, itching, skin disease, dry skin, hair loss, dry mouth, flatulence, gastrointestinal abnormalities, heartburn/indigestion, changes in taste, dental abscess, dehydration, liver dysfunction, hepatobiliary/pancreatic abnormality, infection with grade 3/4 neutrophil count, infection with grade 1/2 neutrophil count, alkaline phosphatase abnormality, AST increase, bilirubin increase, γ-GT increase , hyperglycemia, hyponatremia, hypoglycemia, hypokalemia, diabetes mellitus, neuropathy, confusion, sensory neuropathy, cough, pleural effusion, flu-like symptoms, muscle weakness, blurred vision, vasculitis, hypertension, hypotension, gastrointestinal bleeding, mood swings, upper respiratory tract infection, renal failure, frequency, urine color change, neuropathy-motor, tremor, diplopia, CNS cerebrovascular ischemia, hemorrhoids, dysphagia, multiple spots, nail changes, tremor, weight gain, and hyperpigmentation.

3) The table below shows the adverse events of grade 3 or higher based on CTCAE v3.0 that occurred in more than 2% of 1,062 patients in the phase 3 clinical trial for pancreatic cancer in terms of the number of patients (%) for the control group (gemcitabine/capecitabine)(N=358) and the study group (GV1001)(N=354). Frequency is defined as: very common (≥1/10); common (≥1/100 to <1/10); not common (≥1/1,000 to <1/100), rarely (≥10,000 to <1/1,000), very rarely (<1/10,000).

| Grade 3* or higher adverse events (number of patients (%)) in more than 2% of patients in the phase 3 clinical trial for pancreatic cancer(N=1,062) | | | |
| --- | --- | --- | --- |
|  | Control group  (gemcitabine/capecitabine)  (N=358) | Study group  (gemcitabine/capecitabine/  leukine/GV1001)  (N=354) | Note |
| **Blood and lymphatic system disorders** | | | |
| Neutropenia | 68 (19.0) | 79 (22.3) | Very common |
| Thrombocytopenia | 15 (4.2) | 23 (6.5) | Common |
| Hemoglobin decrease | 11 (3.1) | 13 (3.7) | Common |
| Leukopenia | 8 (2.2) | 7 (2.0) | Common |
| **General disorders and administration site conditions** | | | |
| Pain | 34 (9.5) | 42 (11.9) | Very common |
| Fatigue | 27 (7.54) | 44 (12.4) | Very common |
| Fever | 9 (2.5) | 11 (3.1) | Common |
| **Gastrointestinal disorders** | | | |
| Gastrointestinal obstruction | 16 (4.5) | 24 (6.9) | Common |
| Vomiting | 17 (4.7) | 22 (6.2) | Common |
| Nausea | 13 (3.6) | 20 (5.6) | Common |
| Diahrrea | 17 (4.7) | 11 (3.1) | Common |
| Ascites | 15 (4.2) | 19 (5.4) | Common |
| Constipation | 6 (1.7) | 11 (3.1) | Common |
| **Skin and subcutaneous tissue disorders** | | | |
| Rash – Hand and Foot | 27 (7.5) | 7 (2.0) | Common |
| **Hepatobiliary/pancreatic disorders** | | | |
| Hepatobiliary abnormalities | 5 (1.4) | 10 (2.8) | Common |
| Liver dysfunction | 8 (2.2) | 5 (1.4) | Common |
| **Infections and infestations** | | | |
| Infection with unknown neutrophil count | 24 (6.7) | 24 (6.9) | Common |
| Infection with normal to grade 1-2 neutrophil count | 17 (4.37) | 10 (2.8) | Common |
| Infection | 15 (4.2) | 9 (2.5) | Common |
| Infection with grade 3-4 neutrophil count | 5 (1.4) | 9 (2.5) | Common |
| **Metabolism and nutrition disorders** | | | |
| Bilirubin increase | 19 (5.3) | 18 (5.1) | Common |
| ALT increase | 8 (2.2) | 12 (3.4) | Common |
| GGT increase | 10 (2.8) | 4 (1.1) | Common |
| **Respiratory, thoracic and mediastinal disorders** | | | |
| Respiratory failure | 13 (3.6) | 8 (2.3) | Common |
| **Vascular disorders** | | | |
| Thrombosis/embolism | 35 (9.8) | 21 (5.9) | Common |
| *CTCAE (common terminology criteria for adverse events) V. 3.0 | | | |

Grade 3 or higher adverse events in less than 2% of patients included AST abnormality, febrile neutropenia, arm edema, hypokalemia, rash, pleural effusion, myocardial ischemia, hypotension, diabetes, neurological abnormality, confusion, mucositis, CNS ischemia, hypoglycemia, mood swings, renal failure, allergic reactions, cardiac abnormalities, hypercalcemia, gastrointestinal perforation, pruritus, gastrointestinal stenosis, urine retention, hypoalbuminemia, adverse pulmonary reactions, chills, drowsiness, supraventricular arrhythmias, syncope, thromboembolism, arthritis, cough, skin disease, endocrine system abnormality, flatulence, flu-like symptoms, bleeding, heartburn, intestinal obstruction, metabolic/laboratory abnormality, muscle weakness, pancreatitis, pneumonia, vascular system abnormality, vasovagal abnormality, weight loss, hyperacidity, arterial disease Injury, bronchospasm, cardiac arrhythmias, cardiopulmonary arrest, CNS bleeding, diplopia, dizziness. Reproductive system edema, organ edema, enteritis, esophagitis, gastrointestinal cavity, fracture, gastritis, pulmonary hemorrhage, hypoxia, prothrombin INR abnormality, joint abnormality, left ventricle abnormality, lymphatic vessel abnormality, motor neuropathy, ocular abnormality, phlebitis, pneumothorax, mental illness, kidney abnormality, convulsions, speech disturbances, taste changes, gastrointestinal ulcers, and wound complications.

4) Among 1,062 patients in the phase 3 clinical trial for pancreatic cancer, 15 patients (4.19%) in the control group (Gemcitabine/Capecitabine)(N=358) and 13 patients (3.67%) in the study group (GV1001)(N=354) stopped taking the drug due to side effects.

### Contradictions to Administration

1. Those with hypersensitivity to this drug, Leukine® (sargramostim, rhu GM-CSF), gemcitabine, capecitabine, and components of this drug
2. Pregnant or lactating women, or women who may become pregnant (refer to ‘6. Administration to Pregnant Women and Nursing Women’)
3. Patients with moderate to severe hepatic and renal impairment (creatinine clearance less than 30 mL/min)
4. Intracerebral metastasis or meningioma
5. Uncontrolled angina
6. Patients who received radiation therapy within 4 weeks of treatment initiation
7. Known Malabsorption Syndrome
8. Patients taking drugs that affect the immune system (in the case of steroids, it is possible to temporarily use them to relieve symptoms related to tumors)

### Dosing with Caution

1. Patients with mild-to-moderate renal impairment (adverse events may easily occur due to decreased renal function)
2. Senior

## GM-CSF

The adverse events and precautions for use in this clinical trial predicted from the adverse events reflected in the label of GM-CSF (Leukine®) are as follows.

### Expected Adverse Events

1. Mental nervous system: Fever, headache, fatigue, chills, asthenia, confusion, and convulsions may appear.
2. Central nervous system: syncope, dizziness, increased intracranial pressure, and cerebrovascular disease may occur.
3. Systemic abnormalities: Chest pain may appear.
4. Digestive system: nausea, vomiting, abdominal pain, and poor neurological appetite may appear.
5. Respiratory system: dyspnea, pulmonary edema, and bronchial cramps may appear.
6. Circulation system: Vascular edema and low blood pressure may appear.
7. Musculoskeletal system: Musculoskeletal pain and muscle pain may appear.
8. Skin and accessory organs: Rashes may appear.
9. Other: itching, capillary leakage syndrome, injection site reaction (SC injection), stiffness, stomatitis, increased sweating, peripheral edema, sensory abnormalities, heart failure, heart rhythm abnormalities, etc. may appear.
10. Mild or moderate adverse reactions include stiffness, difficulty breathing, fever, nausea, vomiting, chest pain, helplessness, hypotension, and rash at the initial administration of the drug, and these symptoms were rarely managed by requiring discontinuation of drug administration.
11. Expression of pulmonary signs such as coughing, fever, and dyspnea associated with radiation signs of pulmonary infiltration and pulmonary functional devastation may be preliminary signs leading to respiratory failure or adult dyspnea syndrome (ARDS). Suspension of administration of this drug and appropriate treatment are considered.
12. Acute or severe or life-threatening hypersensitivity reactions, including anaphylaxis, vascular edema, and bronchial contraction, can occur in patients receiving this drug, so if such reactions occur, stop administration immediately and do not re-administrate.
13. If reactions such as pleural inflammation, pleural effusion, pericarditis, or pericardial effusion appear, the administration is stopped.

### Prohibition of Administration

1. Patients who overreacted to the components of GM-CSF or injections.
2. with a history of thrombocytopenia valvular disease.
3. Patients with myeloid tumors
4. Patients with increased Myeloid blast in bone marrow or peripheral blood (≥5%) after chemotherapy is completed.

### Careful Administration

1. Patients with bone marrow malignancy who have leukopenia and are receiving chemotherapy
2. Patients with a history of lung disease (which may worsen lung function or cause dyspnea) should be closely monitored when administering the drug.
3. During rhu GM-CSF treatment, autoimmune disease may be found or exacerbated, and a predisposition or history of autoimmune disease, including thrombocytopenia, should be considered when administering the drug.
4. Pregnant women and patients with a history of hypertension or epilepsy should be administered with caution.

## Gemcitabine

See Appendix 6.

## Capecitabine

See Appendix 7.

# CRITERIA FOR STUDY DISCONTINUATION AND SUBJECT WITHDRAWL

## Study Discontinuation

If the investigator determines that the clinical trial should be discontinued according to the results observed during the clinical trial, part or all of the trial may be discontinued in consultation with the sponsor. The sponsor may suspend part or all of the clinical trial for safety or management reasons.

In this trial, an interim analysis is performed on data up to the time of about 43 deaths, and IDMC suggests whether to proceed with this clinical trial based on the results. If the futility of the investigational product (GV1001) is shown according to the results of the interim analysis, the clinical trial may be terminated early, and the final judgment is made by the sponsor.

In case of premature termination or suspension of the clinical trial, the principal investigator should immediately notify the subject of this fact so that appropriate measures and follow-up can be made. The electronic case record for subjects who have progressed up to the time of suspension, the clinical trial progress status and results are organized and delivered to the clinical trial sponsor, and all test-related data (completed, incomplete or unlisted electronic case records and investigational products, etc.) must be returned to the clinical trial sponsor. If the trial is suspended, it may be reported to the IRB and the Ministry of Food and Drug Safety, and the entire clinical trial schedule may be suspended according to the decision of the IRB.

## Withdrawal Criteria

Subjects can stop participating in the study at any time during the study. Reasons for early discontinuation of treatment during the study included:

1. The subject’s decision to discontinue study participation
2. Confirmed PD according to RECIST and irRC
3. Any AE or toxicity due to which, in the opinion of the investigator, study participation is no longer possible (including severe renal impairment as evidenced by CCr <30 mL/min)
4. Confirmed pregnancy
5. Requiring long-term steroids for a concurrent disease other than pancreatic cancer (low-dose steroids for alleviation of symptoms were allowed at the discretion of the investigator.)
6. Treatment with protocol-defined prohibited drugs or therapy after randomization
7. At least one of the followings with regard to IP dosing:

- Subject miss 2 consecutive doses or 3 non-consecutive GV1001 administrations during the entire treatment course
- Subject miss 2 consecutive cycles of gemcitabine/capecitabine or 3 non-consecutive cycles during the entire treatment course

1. Worsening of any disease other than pancreatic cancer which, in the opinion of the investigator, interferes with further treatment
2. Violation of the inclusion/exclusion criteria
3. Lost to follow-up during the study
4. Other reasons for which the investigator determines that the subject must stop participating in the study.

## Handling of Premature Withdrawal

All premature withdrawals from the study are to be documented for cause and date of withdrawal in applicable electronic case report forms (eCRFs) and medical records. If possible, subjects are followed up for causes of withdrawals; if the cause was an AE, the outcome of the AE is to be reported according to criteria, methods, and reporting of safety assessment including AEs.

Even if the study is not completed according to the protocol, all subjects who administered the IP are required to complete all scheduled tests at EOS, if possible. Those who had at least one dose of the IP but were prematurely withdrawn without completing the study are also to have all scheduled tests of the last time point for safety assessment. Every effort should be made by the investigator to contact subjects who are lost to follow-up or obtain detailed information about the cause of loss to follow-up. Screening number and randomization number that were assigned once are not reassigned. A new subject is always assigned a new subject number (screening number and randomization number).

## Replacement of Subjects

Subjects who were prematurely discontinued from the study were not to be replaced.

## Post-EOT/EOS Treatment

After EOT, a subject is to receive treatment that, in the opinion of the investigator, is in the best interest of the subject. In addition, all subjects except for those who withdrew their informed consent to study participation are followed up after EOT every 12 weeks until death or EOS.

# STATISTICAL METHODS

## Criteria and Methods for Assessment

### Primary Efficacy Endpoint

Overall Survival, OS: OS is defined as time from date of randomization to date of death from any cause. OS was assessed at EOS. OS iscalculated as follows:

Overall Survival (number of days) = date of death by any cause – date of randomization + 1 day

If the subject did not die until EOS, they were censored at the date last known alive.

### Secondary Efficacy Endpoint

1. Time to tumor progression; TTP

TTP is defined as the time from the date of randomization to the date of progressive disease as measured by CT and calculated as follows:

Time to Tumor Progression (number of days) = date of first documented PD – date of randomization + 1 day

Subjects without PD (Progressive Disease) are censored on the date of last evaluable diagnostic imaging (CT scan). Deaths without PD were censored on the date of death. Those without evaluable diagnostic imaging data are censored on the last day their survival was confirmed. Those who started receiving another treatment for pancreatic cancer prior to the first PD are censored on the date of last evaluable diagnostic imaging performed prior to initiation of another treatment.

1. Objective response rate (by RECIST and irRC)

ORR was defined as a proportion of subjects confirmed as ‘complete’ or ‘partial’ by CT (or MRI) based on RECIST and irRC.

1. Clinical benefit response (CBR)

CBR was defined as a proportion of subjects confirmed as ‘responders’ based on the following criteria:

Pain intensity (VAS)

: ≥50% decrease score from baseline in VAS (=improved)

ECOG

: 1-stage decrease from baseline in ECOG (=improved)

**‘Responder’**

: Improved in both pain intensity and ECOG (improved)

**OR,**

Stable for either variable and improved for the other variable (improved)

**‘Non-responder’**

: Worsening of either variable (worsening)

**‘Stable’**

: Stable for both variables (stable)

Weight change

**‘Responder’**

: ≥7% increase from baseline in weight

**‘Non-responder’**

: Weight decrease or no change (<7% increase from baseline in weight)

* CBR assessment at the time of VAS/ECOG assessment: baseline, Weeks 5, 9, 13, 17, 21, 25, and 29, and every 12 weeks thereafter

1. Assessment of correlation between eotaxin level and treatment response
2. Change from baseline in QoL (EORTC QLQ-C30, EQ-5D) scores
3. Change in CA19-9 over time

### Exploratory Efficacy Endpoints

(1) Assessment of immune response:

- T-cell proliferation

- Determination of positive T-cell proliferation

Positive T-cell proliferation was to be determined when tested positive by Method 1 or 2.

- (Method 1) 3H-thymidine uptake assay

Using the measured T-cell (count per minutes [c.p.m.]) value, *Stimulatory Index (SI) was calculated; the test was determined positive if SI was ≥2.

*Stimulatory Index (SI) = c.p.m. (test result at each testing time point after initiation of GV1001 dosing) / c.p.m. (test result before GV1001 dosing at the baseline visit)

- (Method 2) CFSE assay

In comparison with the value obtained before the first dose of GV1001 at the baseline visit (Visit 3), T cell proliferation measured at pre-defined post-dose time points was calculated as *SI; The test was determined positive if SI was ≥2 OR ** difference in the number of T cell divisions was ≥1.

*Stimulatory Index (SI) = ***T cell population at the testing time point after initiation of GV1001 dosing/***T cell population before GV1001 dosing at the baseline visit

** Difference in the number of T cell divisions = number of T cell divisions at the testing time point after initiation of GV1001 dosing - number of T cell divisions before GV1001 dosing at the baseline visit

***T cell population refers to the number of T cells left-shifted from the undifferentiated parent peak on the CFSE content analysis by flow cytometer; cell number can be represented as % by gating.

-Delayed-type hypersensitivity (DTH*)

* Positive DTH: The test was determined “positive” if a red spot occurring 48 hours post-dose was no smaller than 5 mm * 5 mm (mean ≥5 mm, 5+5/2).

If it was difficult to determine the size, a spot with the smallest diameter of ≥3 mm was determined positive (e.g., positive for 3 mm * 7 mm; negative for 2 mm * 8 mm).

**Subgroup analysis**

1. Survival and objective response rate by DTH or T-cell proliferation

Subjects were categorized as ‘responders’ for those with positive DTH or T-cell proliferation outcomes versus ‘non-responders’ for the rest of the subjects; by category, OS and ORR were assessed in the same manner as above.

1. Change in CA19-9 by tumor response

Subjects with complete response (CR) or partial response (PR) were classified as ‘responders’ and assessed for change in CA19-9.

## Statistical Methods

### General Principles for Analysis of Results

Statistical analysis will be performed using SAS^®^ 9.4 (SAS Institute, SAS Circle, Cary, NC, USA). Except for the primary efficacy endpoint, and unless otherwise specified, two-sided tests are to be performed at a significance level of 0.05.

Efficacy will be determined primarily in the FAS while safety will be analyzed in the safety set.

### Efficacy Set

**FAS (Full Analysis Set)**

The FAS includes all randomized subjects who met all inclusion/exclusion criteria and had at least one efficacy assessment data (survival data).

All subjects will be analyzed according to randomized treatment assignment.

**PPS (Per Protocol Set)**

The PPS includes all subjects in the FAS who had no major protocol deviations. Major protocol deviations are as follows. Inclusion of individual subjects in the PPS is determined in blind meeting according to protocol deviations.

- Violation of the inclusion/exclusion criteria
- Randomization error: assigned to a different treatment group instead of the assigned treatment group
- Treated with prohibited drugs that might affect efficacy
- Treatment initiated prior to randomization

#### Safety Set

The safety set includes all subjects who had at least one dose of the IP. All subjects will be analyzed according to treatment they actually received during the study period regardless of their randomized treatment group.

### Demographic Information and Other Baseline Characteristics

For all subjects enrolled in this study, demographic information (gender, age, body weight, etc.) and baseline characteristics are analyzed and presented by group. For continuous variables (e.g., age, body weight, etc.), the number of subjects, mean, standard deviation (SD), median, and range are described. For categorical variables (e.g., gender, cytological diagnosis, etc.), frequency (N) and percentage (%) were presented.

Past medical history and current disease are classified by system organ class (SOC) and preferred term (PT) of MedDRA 23.0 and summarized by treatment group. Drugs administered before and during the study are coded by ATC code 2020 and summarized as anatomical category and therapeutic category by treatment group.

### Efficacy Analysis

#### Primary Efficacy Endpoint

The primary efficacy endpoint is OS. To compare the study group and the control group, satisfaction of the proportional hazard assumption is examined: the stratified log-rank test is used if the model satisfied the assumption; and the stratified generalized Wilcoxon test (Gehan test) is used if not. In each analysis, progression of pancreatic cancer (locally advanced, metastatic) is considered as a stratification factor, and the proportional hazard assumption is examined using log(-log S(t)) by log(time) graphs. By group, 25%, 50%, and 75% quantile estimates and their 95% confidence intervals (CIs) as well as Kaplan-Meier curves are presented. An analysis is performed using the Cox PH model with progression of pancreatic cancer as a covariate, and the estimated hazard ratio and its 95% CI are presented.

In this study, one interim analysis is performed. To maintain overall one-sided type 1 error at 2.5%, one-sided significance level for the final analysis is set at 0.0245.

#### Secondary Efficacy Endpoints

1. Time to tumor progression; TTP

TTP is analyzed in the same manner as the primary efficacy endpoint.

1. Objective response rate (by RECIST and irRC)

The number of subjects with CR or PR as well as percentage and its 95% CI are presented for each group, and Chi-square test or Fisher’s exact test is performed for the difference between treatment groups.

1. Clinical benefit response

The number of subjects assessed as clinical benefit responders as well as percentage and its 95% CI are presented for each group, and Chi-square test or Fisher’s exact test is performed for the difference between treatment groups.

1. Assessment of correlation between eotaxin level and treatment response

Comparison of OS by eotaxin level (high, low) within the control group is analyzed using the same statistical methods as in the primary efficacy assessment. Additionally, comparison of OS between the study group and the control group among those with high eotaxin level is analyzed using the same statistical methods as in the primary efficacy assessment.

1. Change from baseline in QoL (EORTC QLQ-C30, EQ-5D) scores

Descriptive statistics will be provided for scores for each group. Two sample t-test or Wilcoxon rank sum test will be performed for change from baseline in scores between treatment groups.

1. Change in CA19-9 over time

For change from baseline in CA19-9 between treatment groups is analyzed using Mixed model repeated measures (MMRM).

#### Exploratory Efficacy Endpoints

(1) Assessment of immune response

- T-cell proliferation
- Delayed-type hypersensitivity (DTH)

The number of subjects with positive T-cell proliferation or DTH outcomes, percentage and its 95% CI were presented.

Subgroup analysis

- - - 1. Survival and objective response rate by DTH or T-cell proliferation

A subgroup analysis will be performed on OS and proportion of CR and PR only amongst those with positive T-cell proliferation or DTH outcomes. The same analytical method used for the relevant variable is applied.

- - - 1. Change in CA19-9 by tumor response

Amongst those who had CR or PR in tumor assessment, change in CA19-9 since they had a response is compared between the study group and the control group using MMRM.

### Safety Analysis

Safety is analyzed in the safety set based on AEs, abnormal findings of clinical laboratory tests, abnormal changes in body weight and pulse, etc.

In addition, the difference among groups in the incidence of AEs and abnormal findings of clinical laboratory tests is analyzed by appropriate statistical methods depending on the characteristics of relevant variables.

### Adverse Events

The analysis of adverse events is performed using treatment-emergent adverse events (TEAEs). AEs are coded by MedDRA 23.0, and severity of AEs is classified according to NCI CTCAE 4.03.

By treatment group, percentage of subjects with AEs and its 95% CI are presented. All AEs will be summarized by severity. In addition, AEs related to the IP and SAEs will be separately summarized.

The incidence of AEs and ADRs between treatment groups is analyzed by Chi-square test or Fisher’s exact test.

In addition, the percentage of subjects with Grade 3 or 4 AEs based on NCI CTCAE 4.03 and its 95% CI are presented, and the difference between treatment groups will be analyzed by Chi-square test or Fisher’s exact test.

### Other Safety Endpoints

Continuous data such as hematology and blood biochemical test results, pulse rate, weight, etc. provide descriptive statistics (mean, standard deviation, minimum, maximum, median, etc.) for each group and visit, and categorical variables presents frequency and ratio.

All clinical laboratory test items are classified according to the NCI CTCAE criteria, and items with toxicity corresponding to CTCAE grade 3 or 4 are listed separately.

Categorical data such as urinalysis presents the frequency and ratio for each category.

Subjects who showed abnormal results in laboratory tests are listed and presented by measurement time and group, and a shift table is presented for changes from baseline.

### Handling Missing Data

Unobserved events (patients who failed to follow-up) are treated as censored in the survival analysis. In the case of response evaluation, the NRI (Non responder imputation) method is applied, and CA19-9 analyzes as observed without replacing missing values.

## Independent Data Monitoring Committee (IDMC) and Interim Analysis

### Independent Data Monitoring Committee

#### Composition

It is composed of three independent members (two clinical experts and one statistician) not related to this clinical trial, and the meeting is held in a closed format without participating investigators. The IDMC committee list will be reported to each sites’ IRB.

#### Meeting Schedule

After the initiation of the clinical trial, an interim analysis (at the time of 43 expected deaths) is conducted, the interim analysis results are reviewed and meetings are held, and all accumulated safety data are reviewed regularly.

### Interim Analysis

Interim analysis analyzes data up to the time of about 43 death events, and all planned procedures, such as subject registration and follow-up, proceed as planned. Interim analysis targets the primary and secondary efficacy variables. These results will be reviewed by IDMC, and IDMC will deliver a recommendation on whether to continue or discontinue the trial to the sponsor in consideration of the efficacy results of the interim analysis, drug safety information, and clinical aspects. IDMC members and interim analysis statisticians must maintain the security of the results, and investigators involved in this study cannot participate in the review of the results. The final decision on whether to proceed with the clinical trial is made by the sponsor.

IDMC must comply with IDMC charter according to ICH GCP guideline.

#### Stopping guidelines

The criteria for early termination of clinical trials suggest two things: Significance and Futility.

To set the reference value for the above two criteria and to maintain the overall unilateral type-error rate of 2.5%, the group sequential method with the O'Brien-Fleming type-alpha spending function was used, and the log rank test was applied as a statistical test method.

The table below presents the results for this.

| Boundary Information (Standardized Z/p-value) | | | | | | | | |
| --- | --- | --- | --- | --- | --- | --- | --- | --- |
| Non binding Beta Boundary | | | | | | | | |
| Analysis Stage | Information  Level | Number  of Events | Boundary Values | | | | Cumulative Error Spending | |
|  |  |  | Standardized  Z - Upper | | Nominal  alpha - Upper | |  |  |
|  | Proportion | D | Beta † | Alpha ‡ | Beta | Alpha | Beta | Alpha |
| Interim  Analysis | 0.5 | 42.06 | 0.55939 | 2.96259 | 0.28795 | 0.00153 | 0.06993 | 0.00153 |
| Final Analysis | 1 | 84.13 | 1.9686 | 1.9686 | 0.0245 | 0.0245 | 0.2 | 0.025 |

† Beta: Futility criteria

‡ Alpha: Significance criteria

SAS proc seq design is used


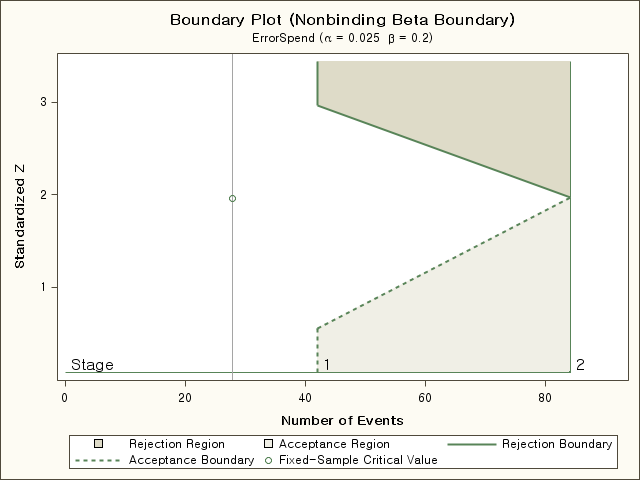

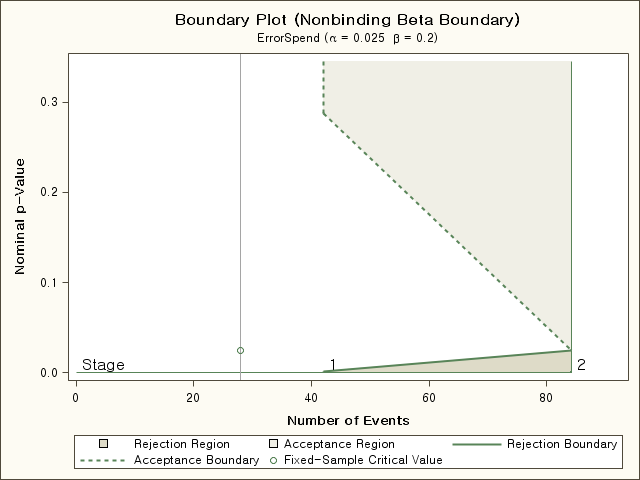


Based on the above results, the criteria for early termination through interim analysis are set as follows (when performed as a one-sided test for log rank test)

(1) GV1001 shows futility (Z<0.55939)

: Early terminate the trial.

(2) GV1001 does not show significance (0.55939≤Z≤2.96259)

: Keep doing the trial and complete it.

(3) GV1001 shows significance (Z>2.96259)

:Submit the significance result of the interim analysis to the Ministry of Food and Drug Safety and cancel the treatment code.

# CRITEERIA, METHODS AND REPORTING OF SAFETY ASSESSMENT INCLUDING ADVERSE EVENTS

## Definition of Adverse Events

An adverse event (AE) is any untoward medical occurrence in a patient or clinical investigation subject administered a pharmaceutical product and which does not necessarily have a causal relationship with this treatment. An adverse event (AE) can therefore be any unfavourable and unintended sign (including an abnormal laboratory finding), symptom, or disease temporally associated with the use of an investigational product, whether or not related to the investigational product. However, any change derived from the normal course of growth and development is not considered an AE if the frequency and seriousness of the change is not substantially different from the expected level.

In this study, determination of PD was not reported as an AE.

## Definition of Serious Adverse Event, SAE

A serious AE (SAE) is any AE that:

1. Results in death or is life-threatening;
2. Requires hospitalization or prolonged hospitalization;
3. Results in persistent or significant disability and incapacity;
4. May have caused a congenital anomaly/birth defect; or
5. Is other medically important circumstances.

* If the subject’s condition was applicable to any of the followings listed, the event was not considered an SAE:

- Planned hospitalization unrelated to the study.
- Hospitalization as a routine procedure for the treatment of another disease
- General and elective treatment unrelated to the subject’s condition (e.g., hemorrhoid surgery, hospitalization for IP dosing)
- Emergency treatment not requiring hospitalization (not life-threatening)

In this study, a death caused by PD is not reported as an SAE unless the investigator considered the death to be potentially related to the IP.

## Assessment Criteria of Adverse Events

**Adverse Event**

All adverse events that have occurred since the time of registration should be listed by body organ with detailed descriptions. The relevance of the expressed adverse reactions is evaluated according to the criteria presented in Section 16.3.2, “CASUAL RELATIONSHIP TO THE INVESTIGATIONAL PRODUCT”, and the adverse events evaluated as likely to be related and those evaluated as unknown are regarded as the adverse reactions. The severity of the adverse events depends on the CTCAE.

**Clinical Laboratory Test Values**

The degree of toxicity is assessed according to the CTCAE.

### Severity of Adverse Events

The severity of adverse events is evaluated on a scale of 1 to 5 according to CTCAE.

| **Severity** | **Grade** | **Description** |
| --- | --- | --- |
| Mild | 1 | Symptoms are hardly recognizable to the subject and do not interfere with daily activities; generally, prescribed drugs are not required to alleviate symptoms. |
| Moderate | 2 | Symptoms are severe enough to make the subject uncomfortable; the subject may continue to be on the study, but may require treatment for symptoms. |
| Severe | 3 | Symptoms are severe enough to make the subject severely uncomfortable so that he/she can no longer participate in the study; seriousness of the symptoms may lead to discontinuation of the IP; treatment may be provided and/or the subject may be hospitalized for symptoms. |
| Life-threatening | 4 | Symptoms of the event may lead to an immediately life-threatening condition; this does not include an event that had occurred in a more severe form that might have caused death. |
| Death | 5 | The subject is dead due to an AE. |

### Causal Relationship to the investigational product

The extent of a relationship between IP dosing and an AE is determined by the investigator based on the following criteria:

- - 1. Definitely related
- Evidence of the IP administration exists.
- The temporal relationship between administration of the IP and occurrence of the AE is plausible.
- AE can be explained by the administration of the IP with higher probability than any other reasons.
- AE disappears by discontinuing IP administration.
- The outcome of re-challenge (only if feasible) is positive.
- AE shows a pattern consistent with the information already known about the IP or products of the same class.
  - 1. Probably related
- Evidence of the IP administration exists.
- The temporal relationship between administration of the IP and occurrence of the AE is plausible.
- AE can be explained by the administration of the IP with higher probability than any other reasons.
- AE disappears when administration is discontinued.
  - 1. Possibly related
- Evidence of the IP administration exists.
- The temporal relationship between administration of the IP and occurrence of the AE is plausible.
- AE is considered to have been caused by the IP administration with the same level of probability as the other possible reasons.
- AE disappears (if conducted) by discontinuing the administration.
  - 1. Probably not related, remote
- Evidence of the IP administration exists.
- Other causes provide plausible explanations.
- The result of discontinuation of administration (if conducted) is negative or ambiguous.
- The outcome of re-challenge (only if feasible) is negative or ambiguous.
  - 1. Definitely not related, none
- Investigational products not dosed to the subject.
- Or, the periodic sequence between the administration of the investigational products and the occurrence of an AE is not valid.
- Or, there is another apparent cause for the AE.
  - 1. Unknown, unassessable
- Insufficient evidence for appropriate judgment of a relationship.
- Low quality of supporting data or inconsistency across data.

## Reporting Adverse Events

**Serious Adverse Events (SAE)**

All SAEs occurring during the study period are reported by the PI and the sub-investigator to the sponsor within 24 hours after the event was known, regardless of its relationship to the IP, by telephone, fax, or email, and to the IRB according to the site-specific regulations. If possible, the initial report is to contain all information required by the SAE form; the completed form is to be faxed to the IRB. In addition, the information is also documented on the AE page of the eCRF.

Upon receipt of the initial report, the IRB reviews the information and if necessary contacts the sub-investigator for further information. Based on the information, the IRB investigated causal relationship of the event to the IP. If necessary, a follow-up report containing all new information about SAE is prepared; the follow-up report is sent to the IBR or is separately retained.

All suspected unexpected serious adverse reactions (SUSARs) are reported by the sub-investigator to the sponsor (or a designee) who, in turn, immediately reported to the Minister of Food and Drug Safety in compliance with KGCP. “SUSARs” are serious adverse reactions that differ from the information described in the label (including product report) in terms of profile, severity, specificity, and outcome. SUSARs that resulted in death or were life-threatening are reported within 7 days from when the event was reported or known to the sponsor (or a designee), with an additional report containing detailed information submitted within 8 days from the initial report.

All other SUSARs are reported within 15 days from when the event was reported or known to the sponsor (or a designee). Attachment No. 77 ADR report form completed by the sponsor, together with Attachment No. 76 Expedited report form completed and submitted by the sub-investigator, is submitted by the sponsor to the Minister of Food and Drug Safety within a set period of time. Any additional information continued to be reported until the event is ended.

**Non-serious AEs**

Non-serious AEs are reported on the AE pages of eCRFs. Upon the end of study, the PI reviewed and assessed for the CSR all non-serious AEs that are reported during the study.

**AEs occurring after EOT**

AEs that occurred after EOT are reported according to the above described procedures if they are considered to be attributable to the IP.

**Pregnancy**

For subjects who were found to be pregnant during the treatment period in this study, IP dosing is discontinued, and EOT is implemented.

## Follow-up of AEs

All AEs occurring during the study period are followed up by the PI or the sub-investigator if possible until the event is returned to pre-dose or baseline level or the event is considered by PI or the sub-investigator to be stabilized or a follow-up is considered to be no longer necessary.

# INFORMED CONSENT FORM, COMPENSATION AGREEMENT AND TREATMENT FOR DROPOUTS

## Subject Statement and Consent Form

Subjects should give written consent after hearing and understanding the purpose and method of the study and the expected benefits and risks from the principal investigator or the person in charge. The principal investigator or the person in charge should ensure that the signature and date of the subject consent form are properly recorded before performing the protocol procedure and keep it.

Refer to the Attachment 1. subject statement and consent form

## Reimbursement for Patients

Refer to the Appendix 2. Reimbursement for patients

## Treatment Criteria for Subjects After Clinical Trial

For the treatment and treatment of subjects who have been dropped out of this clinical trial and who have been treated with investigational drugs, the best treatment for the patient is performed according to the judgment of the investigator.

# MEASURES FOR THE SAFETY PROTECTION OF SUBJECTS

This clinical trial will be conducted scientifically and ethically in accordance with Good Clinical Practice and applicable regulations. In addition, this clinical trial will be conducted in accordance with the Declaration of Helsinki, respecting human dignity and rights and not causing any disadvantage to the subject. The clinical trial institution is equipped with the facilities and professional manpower necessary for the clinical trial so that the clinical trial can proceed properly as stipulated in this trial plan, and it does its utmost to protect the safety of subjects. The principal investigator or person in charge should be fully aware of the adverse events and precautions specified in this protocol in advance, and if necessary, if a serious adverse event occurs during the trial, stop the clinical trial of the subject and take appropriate measures before reporting the clinical trial Notify the Board (IRB).

## Record

The principal investigator records the appropriate actions and progress for the adverse events in the case record.

## Tumor Progression during the Trial

The subject may end treatment (EOT) and proceed with the treatment deemed best for the subject. After tumor progression is confirmed and treatment is terminated (EOT), follow-up is performed every 12 weeks to confirm survival.

# OTHER MATTERS FOR SAFE AND SCIENTIFIC TRIAL

Good Clinical Practice (KGCP, ICH E6)

The procedures stipulated in this protocol are designed to ensure that Samsung Pharma Co., Ltd. and the principal investigator or person in charge conduct, evaluate, and record the results of this study in compliance with the fundamental spirit of the Good Clinical Practice (KGCP), ICH E6 and the Declaration of Helsinki (Appendix 1. Helsinki Declaration).

## Institutional Review Board (IRB)

Before starting the trial, the investigator must obtain written approval from the IRB for the clinical trial protocol, consent form, data and procedures related to subject recruitment, and subject description to be provided to subjects. The IRB's decision to conduct the trial is communicated in writing to the Investigator and Sponsor prior to the start of the trial.

The principal investigator will report to the IRB about the progress of the trial, serious adverse events, life-threatening problems, or deaths, and must notify the IRB at the end of trials.

## Obligations and Delegation of the Principal Investigator

The principal investigator confirms that all persons participating in the clinical trial are properly familiar with the protocol and its revisions, the drugs used in the clinical trial, and the duties and functions entrusted to them. The Principal Investigator maintains a list of investigators participating in this clinical trial and appropriately qualified persons who have been delegated responsibilities related to the trial.

## Informed Consent

The principal investigator should ensure that information on the nature, purpose, and expected risks and benefits of the clinical trial is provided to subjects in writing and verbally, sufficiently and appropriately. In addition, subjects should be informed that they are free to stop participating in the trial at any time. Subjects should be given time to think about participating in the trial and an opportunity to ask questions about the trial. The informed consent signed by the subject must be obtained before any detailed procedures for the study are performed. The original signed written consent must be kept by the investigator and a copy must be delivered to the subject. (Attachment 1. Subject Information and Informed Consent)

## Approval of Protocol

Before starting the trial, submit the clinical trial protocol and related documents to the IRB and the Ministry of Food and Drug Safety. Samsung Pharma Co., Ltd. and the investigator or the person in charge will notify each other in writing that all ethical/legal requirements have been met before the first subject is allowed to participate in the trial. Drugs used in clinical trials will be delivered to the principal investigator (or designee) after all ethical/legal requirements for initiating the trial have been met.

## Revision of Protocol

If the contents of the protocol are changed, the principal investigator reviews, signs, and obtains written consent. The revised contents will be implemented after obtaining approval from the IRB of the site and/or the Ministry of Food and Drug Safety in accordance with the applicable regulations.

## Confidentiality

All subject names are kept confidential and recorded and evaluated by numbers assigned during the trial. The subject number and initials of the subject will be written in the case record, and if there is a subject name in other documents (pathological report), etc., the subject name will be deleted and provided to the sponsor. Inform the subject that all test data will be treated in strict confidentiality. After the clinical trial is completed, the signed consent form is kept by the person in charge of storage of the institution. By signing this protocol, the principal Investigator agrees to obtain informed consent from the subjects participating in the trial and to undergo inspection upon request. Accordingly, it will inform the subject that the subject's medical record can be audited for the purpose of verifying the information collected by the representative of the sponsor, the IRB, and the Ministry of Food and Drug Safety, and that the information exposed during the audit will be handled under strict confidentiality.

The principal investigator keeps a list of subject numbers and subject names so that the records can be checked later. The clinical trial institution and sponsor shall keep the subject consent form and related data for 3 years after approval of the Ministry of Food and Drug. It will then be discarded according to the institution's SOP or stored by the sponsor if necessary.

## Monitoring and Audit for Protocol Compliance

Monitoring will be conducted by the sponsor Samsung Pharma Co., Ltd. or its agent to confirm whether the clinical trial is conducted according to the KGCP and according to the protocol. During the monitoring of this study, the completeness and clarity of the electronic case record form entry will be checked, comparing with source data, and management tasks will be checked, and the principal investigator or person in charge of the study must cooperate with this.

In addition, the Ministry of Food and Drug Safety, the IRB, and the sponsor may conduct inspections or audit of the site separately from monitoring, and may request to see source documents, case records, and other trial-related documents. In this case, the investigator allows this and must always cooperate with all these processes.

## Collection of Data, Recording and Use of Results

### Collection of Data

The investigator enters the information required in the protocol into the Electronic Data Capture (EDC) system according to the guidelines for preparing the eCRF provided by the sponsor. The monitor will review and verify the data collected in the eCRF against the source document during the monitoring visit. The Monitor (CRA) handles any discrepancies identified in the data, ensures that the data can be corrected promptly, and is recorded by the primcipal Investigator or a person authorized by the Investigator.

The investigator or the person authorized by the principal investigator performs data entry and data correction in the eCRF through the EDC system. All data records and corrections are recorded in the EDC system through the electronic audit trail, and the change history such as user identification of the person who made the correction and and those corrections. The requirements of the EDC system are in accordance with the EDC guidelines stipulated in Title 21, Part 11 of the Code of Federal Regulations (CFR) and the guidelines for processing and managing clinical trial electronic data, which are local regulations.

### Record of the Results

Data entered into the EDC system must be accurate and complete. In accordance with the guidelines for preparing eCRFs provided by the sponsor, the investigator or the person authorized by the principal investigator enters the data required in the protocol into the eCRFs. All eCRF data collected in the EDC system are submitted to the sponsor at the end of the clinical trial through compact disks (CDs) or removable storage media by creating an accurate and complete copy of the input original data.

Any corrections made to the eCRF input require a reason for the change. Investigators review all eCRF data entered into the EDC system and terminate with an electronic signature to certify that they have been reviewed. Reports from the eCRF derived from the source documents should be consistent with the source documents, otherwise discrepancies should be accounted for.

### Submission and Publication of Report

By signing the clinical trial contract, the principal investigator or the person in charge agrees to use the results of this trial for the purposes of registration, presentation, and provision of information for pharmaceutical experts. When the results of this clinical trial are published in academic journals or conferences, the sponsor has the right to review the presentation prior to the presentation or submission of the clinical trial results.

# REFERENCES

1. Saif MW, Advancements in the management of pancreatic cancer. JOP 2013;14(2):112-8
2. 통계로 본 암 현황, 보건복지부·국립암센터, 2014
3. 2012 국가암등록통계, 중앙암등록본부 (2014 발표)
4. Kim, JW, Recent treatment of pancreatic cancer, Korean J Med 2009;77:695-702
5. Cho JH, Recent update of molecular targeted therapy in pancreatic cancer, Korean J Gastroenterol 2013;61:147-54
6. Chari ST, Detecting early pancreatic cancer: problems and prospects, Semin Oncol 2007;34:284-94
7. Ferrone CR, Brennan MF, Gonen M, et al., Pancreatic adenocarcinoma: the actual 5-year survivors. J Gastrointest Surg 2008;12:701-6
8. Kim YT, Chemotherapy for pancreatic cancer, Korean J Gastroenterol 2008;51:111-8
9. Song, SY, Non-operative treatment, Korean Pancreatobiliary association conference meeting session II 2006:155-65
10. Burris HA 3rd, Moore MJ, Andersen J, et al., Improvements in survival and clinical benefit with gemcitabine as first-line therapy for patients with advanced pancreas cancer: a randomized trial, J Clin Oncol 1997;15:2403-13
11. Berlin JD, Catalano P, Thomas JP, et al., Phase III study of gemcitabine in combination with fluorouracil versus gemcitabine alone in patients with advanced pancreatic carcinoma: Eastern Cooperative Oncology Group Trial E2297,J Clin Oncol 2002;20:3270-5
12. Heinemann V, Quietzsch D, Gieseler F, et al., Randomized phase III trial of gemcitabine plus cisplatin compared with gemcitabine alone in advanced pancreatic cancer, J Clin Oncol 2006;24:3946-52
13. Saif MW, New developments in the treatment of pancreatic cancer: highlights from the 44th ASCO annual meeting, Chicago, IL, USA., JOP 2008;9:391-7
14. Moore MJ, Goldstein D, Hamm J, et al., Erlotinib plus gemcitabine compared with gemcitabine alone in patients with advanced pancreatic cancer: a phase III trial of the National Cancer Institute of Canada Clinical Trials Group, J Clin Oncol 2007;25:1960-6
15. Saif MW, Pancreatic cancer: are we moving forward yet?: highlights from the gastrointestinal cancers symposium, Orlando, FL, USA. January 20th, JOP 2007;8:166-76
16. Herrmann R, Bodoky G, Ruhstaller T, et al., Gemcitabine plus capecitabine compared with gemcitabine alone in advanced pancreatic cancer: A randomized, multicenter, phase III trial of the Swiss Group for clinical cancer research and the central European Cooperative Oncology Group, J Clin Oncol 2007;25(16):2212-17
17. Hiyama E, Hiyama K, Clinical utility of telomerase in cancer, Oncogene 2002, 21:643-649
18. Middleton G, Silcocks P, Cox T, et al., Gemcitabine and capecitabine with or without telomerase peptide vaccine GV1001 in patients with locally advanced or metastatic pancreatic cancer (TeloVac): an open-label, randomized, phase 3 trial, Lancet Oncol 2014;15:829-40
19. J. P. Neoptolemos, Greenhalf W, Cox T, et al. Predictive cytokine biomakrers for survival in patients with advanced pancreatic cancer randomized to sequential chemotheraphy comprising gemcitabine and capecitabine (GemCap) followed by the telomerase vaccine GV1001 compared to concurrent chemoimmunotherapy in the TeloVac phase III trial, J Clin Oncol 2014 (TeloVac-eotaxin ASCO Poster 22-05-14)
20. 리아백스주(테르토모타이드염산염) 허가사항
21. Shaw VE, Naisbitt DJ, Costello E, et al., Current status of GV1001 and other telomerease vaccination strategies in the treatment of cancer. Expert Rev. Vaccines 2010;9(9):1007-16
22. Gupta RK, Griffin P, Chang AC, et al., The role of adjuvants and delivery systems in modulation of immue response to vaccines. Adv Exp Med Biol 1996;397:105-13
23. Gupta RK, Siber GR, Adjuvants for human vaccines-current status, probles and future prospects. Vaccine 1995 Oct;13(14):1263-76
24. Singh M, O’Hagan D, Advances in vaccine adjuvants, Nat Biotechnol 1999 Nov;17(11):1075-81
25. Gupta RK, Rost BE, Relyveld E, et al., Adjuvant properties of aluminum and calcium compounds, Pharm.Biotechnol 1995;6:229-48
26. Disis ML, Bernhard H, Shiota FM, et al., Granulocyte-macrophage colony-stimulating factor: an effective adjuvant for protein and peptide-based vaccines, Blood 1996 Jul 1;88(1): 202-10
27. Hung K, H, Hayashi R, Lafond-Walker A, et al., The central role of CD4(+) T cells in the antitumor immune response, J Exp Med 1998 Dec 21;188(12):2357-68
28. Dranoff G, Jaffee E, Lazenby A, et al., Vaccination with irradiated tumor cells engineered to secret murine granulocyte-macrophage colony-stimulating factor stimulated potent, specific, and long-lasting anti-tumor immunity, Proc. Natl. Acad.Sci.U.S.A 1993 Apr 15;90(8):3539-43
29. Gjertsen MK, Bakka A, Breivik J, et al., Graudernack G. Vaccination with mutant ras peptides and induction of T-cell responsiveness in pancreatic carcinoma patients carrying the corresponding RAS mutation, Lancet 1995 Nov 25;346(8987):1399-400
30. Jaffee EM, Schuttee M, Gossett J, et al., Deverlopment and characterization of a cytokine-secreting pancreatic adenocarcinoma vaccine from primary tumors for use in clinical trials, Cancer J. Sci.Am. 1998 May;4(3):194-203
31. Gjertsen MK, Buanes T, Rosseland AR, et al., Intradermal ras peptide vaccination with granulocyte-macrophage colony-stimulating factor as adjuvant: clinical and immunological responses in patients with pancreatic adenocarcinoma, Int.J.Cancer 2001 May 1;92(3):441-50
32. Chianes-Bullock KA, Pressley J, Garbee C, et al., MAGE-A1-, MAGE-A10-, and gp100-derived peptides are immunogenic when combined with granulocyte-macrophase colony-stimulating factor and montanide ISA-51 adjuvant and administered as part of a multipeptide vaccine for melanoma, J Immunol. 2005 Mar 1;174(5):3080-6
33. Tsujino K, Kawaguchi T, Kubo A, et al. Is response rate or stable disease rate a surrogate endpoint for survival in the treatment of advanced non-small cell lung cancer using epidermal-growth factor receptor tyrosine kinase inhibitors?, J Clin Oncol 2008 May 20 Suppl;26:15S:14600
34. Ratain MJ, Eckhardt GS, Phase II studies of modern drugs directed against new targets: If you are fazed, too, then resist RECIST. J Clin Oncol 2004;22:4442-5
35. Dougan M, Dranoff G, Immune therapy for cancer, Annu Rev Immunol 2009;27:83-117
36. Little RF, Pluda JM, Wyvill KM, et al. Activity of interleukin 12 in AIDS-related Kaposi sarcoma. Blood 2006;107:4650-7
37. van Baren N, Bonnet M-C, Dreno B, et al., Tumoral and immunologic response after vaccination of melanoma patients with an ALVAC virus encoding MAGE antigens recognized by T cells, J Clin Oncol 2005;23:9008-9021
38. Kruit WHJ, van Ojik HH, Brichard VG, et al., Phase 1/2 study of subcutaneous and intradermal immunization with a recombinant MAGE-3 protein in patients with detectable metastatic melanoma, Int J Cancer 2005;117:596-604
39. Di Giacomo AM, Danielli R, Guidoboni M, et al., Therapeutic efficacy of ipilimumab, an anti-CTLA-4 monoclonal antibody, in patients with metastatic melanoma unresponsive to prior systemic treatments: clinical and immunological evidence from three patient cases, Cancer Immuno Immunother 2009;58:1297-1306
40. Wolchok JD, Hoos A, O’Day S, et al., Guidelines for the evaluation of immune therapy activity in solid tumors: Immune-related response criteria, Clin Cancer Res 2009;15:7412-7420
41. Eisenhauera EA, Therasseb P, Bogaerts J, et al., New response evaluation criteria in solid tumours: Revised RECIST guideline (version 1.1), EJC 2009;45:228-247
42. Cunningham D, Chau I, Stocken DD, et al., Phase III randomized comparison of gemcitabine versus gemcitabine plus capecitabine in patients with advanced pancreatic cancer, J Clin Oncol 2009;27(33):5513-8
43. Choi JG, Seo JH, Oh SC, et al., A phase II trial of gemcitabine plus capecitabine for patients with advanced pancreatic cancer, Cancer Res Treat 2012;44(2):127-32
44. Bernhardt SL, Gjertsen MK, Trachsel S, et al., Telomerase peptide vaccination of patients with non-resectable pancreatic cancer: a dose escalating phase I/II study, British Journal of Cancer 2006;95:1474-82
